# Insights into Bioactive Constituents from Pericarp of *Garcinia mangostana*: Anti-Inflammatory Effects via NF-κB/MAPK Modulation and M1/M2 Macrophage Polarization

**DOI:** 10.3390/antiox15010128

**Published:** 2026-01-19

**Authors:** Cheng-Shin Yang, Sin-Min Li, Jih-Jung Chen

**Affiliations:** 1 Institute of Biopharmaceutical Sciences, College of Pharmaceutical Sciences, National Yang Ming Chiao Tung University, Taipei 112304, Taiwan; 2 Department of Pharmacy, School of Pharmaceutical Sciences, National Yang Ming Chiao Tung University, Taipei 112304, Taiwan; samuel147samuel147@gmail.com; 3 Department of Medical Research, China Medical University Hospital, China Medical University, Taichung 404333, Taiwan; 4 Traditional Herbal Medicine Research Center, Taipei Medical University Hospital, Taipei 110301, Taiwan

**Keywords:** *Garcinia mangostana*, anti-inflammation, bioactive component, macrophage polarization

## Abstract

Mangosteen (*Garcinia mangostana* L.) has long been used in traditional Southeast Asian medicine to treat inflammatory-related conditions. In this study, three new compounds, including garcimangone A (**1**), garcimangone B (**2**), and the *S*-form of garcimangone C (**3**), and 18 known compounds were isolated and investigated for their anti-inflammatory properties and effects on M1- and M2-associated markers. Among the isolated components, γ-mangostin (**5**), garcinone D (**6**), morusignin J (**15**), and fuscaxanthone C (**16**) showed the most potent NO-inhibitory effects in LPS-stimulated RAW264.7 cells. SAR study revealed that chromeno moiety at C-3,4, oxygen substituents at C-1,3,6,7, and isoprenyl groups at C-2,8 are key structural features that promoted anti-inflammatory activity. Cytokine analysis results indicated that morusignin J (**15**) and fuscaxanthone C (**16**) could modulate the production of pro-inflammatory cytokines, such as TNF-α and IL-6, while modulating the anti-inflammatory cytokine IL-10. Western blot results demonstrated that morusignin J (**15**) modulated the inflammatory response through NF-κB and MAPK signaling and increased the expression of M2-associated markers KLF4 and arginase-1 in LPS-induced RAW264.7 macrophages. Further molecular docking analysis confirmed the high binding affinity of morusignin J (**15**) with key iNOS residues, such as Gln257, Pro344, Glu371, and Hem901, and the in silico prediction supported its potent oral bioavailability and drug-likeness. These in vitro and in silico findings highlight that pericarps of *G. mangostana* possess potential as promising natural sources for functional extracts and bioactive constituents for the development of antioxidative and anti-inflammatory candidates, and warrant further in vivo investigation in the future.

## 1. Introduction

Inflammatory response is a crucial biological process initiated by the immune system to defend from harmful damage from the environment, such as injuries or infections [1]. Upon the activation of an inflammatory response, helper T-cells release cytokines, including ILs, interferon-γ, TNF-α, and GM-CSF, which activate plasma cells, leukocytes, and macrophages to initiate humoral immunity [2]. These cytokines further activate cyclooxygenase (COX) and iNOS, producing prostaglandins and nitric oxide and contributing to inflammatory responses, such as redness, swelling, heat, and pain [3]. Though inflammation is aimed at clearing pathogens or injuries, chronic inflammation arises when pathogens or other stimuli persist, leading to prolonged immune activation and tissue damage over months or even years [4].

Macrophages serve a crucial function in the defense mechanism and maintenance of homeostasis during inflammatory responses, including the phagocytosis of necrotic cells and pathogens, the activation of immune cells, and the facilitation of pathogen clearance [5]. Additionally, macrophages can undergo polarization into distinct phenotypes, including M1 (classically activated) and M2 (alternatively activated), depending on the tissue and microenvironment, referred to as macrophage polarization [6]. As the macrophages are induced to M1-type during inflammation, they release TNF-α, interleukins, iNOS, and COX-2 to activate immune cells and clear pathogens [7]. In contrast, cytokines including IL-4 and IL-13 are involved in the induction of M2 macrophages, contributing to tissue repair and the resolution of inflammation through mediators, such as IL-10 and TGF-β, Arg1, and KLF4 [8].

Clinically, corticosteroids and cyclooxygenase (COX)-targeting nonsteroidal anti-inflammatory drugs (NSAIDs) are primarily applied to manage inflammatory responses [9]. However, their long-term use is often limited by adverse effects and incomplete control of complex inflammatory pathways [10,11,12]. Moreover, these agents primarily modulate the COX-prostaglandin axis and cannot directly address dysregulated reactive oxygen and nitrogen species, which also contribute to chronic tissue injury. Recently, the isolation and evaluation of components from plants offer opportunities for the development of novel therapeutics for inflammatory disease with reduced adverse effects [13]. Thus, developing safer and more effective therapeutic agents for inflammatory diseases from natural sources has become a critical focus of research in addressing the limitations of current therapies while mitigating associated risks.

Oxidative stress plays a central role in the pathogenesis of various diseases, including diabetes, neurodegenerative disorders, and chronic-inflammation-related conditions [14]. Key antioxidant enzymes, such as superoxide dismutase (SOD), glutathione peroxidase (GPx), and nitric oxide synthase (NOS), are crucial in maintaining redox homeostasis and mitigating oxidative damage [15]. Among them, inducible nitric oxide synthase (iNOS) can be rapidly upregulated in activated macrophages under inflammatory conditions, leading to the excessive production of nitric oxide (NO) [16]. Although NO acts as an essential signaling molecule involved in vasodilation and neurotransmission under physiological conditions [17], it can react with superoxide anions (O_2_^−^) to form peroxynitrite (ONOO^−^), a highly reactive nitrogen species. Excessive NO and ONOO^−^ contribute to the increased oxidative stress and cellular damage, including lipid peroxidation, protein dysfunction, and DNA fragmentation [18]. Therefore, the inhibition of iNOS expression and NO overproduction, thereby decreasing ONOO^−^, has emerged as a promising strategy to control both inflammation and oxidative-stress-associated disorders.

*Garcinia mangostana* L. is a Guttiferae plant known as mangosteen, which comprises approximately 40 genera and over 900 species that are predominantly distributed in tropical and subtropical regions [19]. With a sweet and tangy flavor, the fruit’s white aril is commonly consumed fresh, whereas the bitter pericarp is applied in traditional Southeast Asian remedies for ailments ranging from gastrointestinal issues, like abdominal pain and diarrhea, to skin conditions, such as infected wounds and chronic ulcers [20]. In particular, the traditional use of the mangosteen pericarp in treating infected wounds and ulcers implies a role in modulating local immune responses and inflammatory processes, thereby suggesting its potential anti-inflammatory properties [20]. The medicinal value of mangosteen pericarp is primarily attributed to its abundance of xanthones, especially prenylated derivatives, which exhibit diverse pharmacological applications such as anti-inflammatory, antioxidant, anticancer, antimicrobial, antifungal, and antiviral activities [21]. Compared with single-target synthetic drugs, many natural products exert pleiotropic effects by simultaneously modulating inflammatory mediators, oxidative/nitrosative stress, and immune cell phenotypes. Nevertheless, despite the identification of numerous potent bioactive constituents derived from various plant sources [12,13,14,15,16,17,18,19,20,21,22,23,24,25,26,27], numerous bioactive components in various plants remain underexplored. Given the longstanding traditional use of mangosteen pericarp in the treatment of infected wounds, chronic ulcers, and other inflammatory-related skin conditions, this study was conducted to isolate and characterize the bioactive compounds from the pericarp of *G. mangostana* and to evaluate their anti-inflammatory effects.

**Figure 1:**
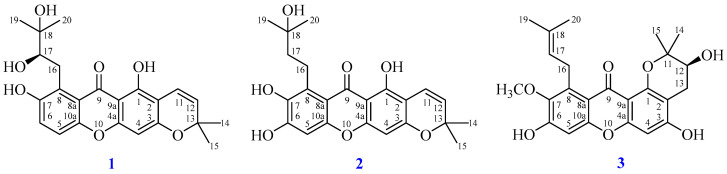
Structures of isolated novel compounds **1**–**3**.

**Figure 2:**
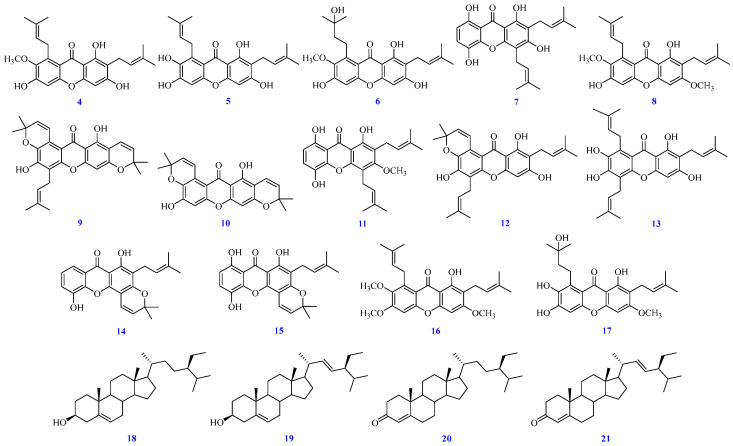
Structures of isolated known compounds **4**–**21**.

In this work, we successfully isolated three new compounds, including garcimangone A (**1**), garcimangone B (**2**), and garcimangone C (**3**) (Figure 1), and 18 known compounds (**4**–**21**) (Figure 2). Through the NMR, UV, IR, and MS, the structures of isolated components were determined. Additionally, brasilixanthone B (**10**), ananixanthone (**14**), morusignin J (**15**), and pruniflorone R (**17**) were first obtained from *G. mangostana* pericarp. Although compound **2** (Reaxys ID: 58432147) appeared in a subsequent publication in 2024 [28], it had already been characterized in our earlier work, which was completed in 2019 and recorded in our previous academic thesis [29]. Furthermore, we evaluated the anti-inflammatory properties of the bioactive extracts and isolated components by examining their effects on M1-associated markers, such as TNF-α, IL-6, and iNOS, and M2-associated markers, such as IL-10, arginase 1, and KLF4, in LPS-induced RAW264.7 macrophages, based on their established roles in pro-inflammatory and pro-resolving macrophage responses.

## 2. Materials and Methods

### 2.1. General Procedures

NMR analyses were conducted using Bruker spectrometers at different field strengths and institutions, including Avance III 400 and 500 MHz, DRX 500 MHz, and Avance III 600-HD 600 MHz (Bruker Corporation, Billerica, MA, USA). The abbreviations, including s, d, dd, t, td, q, m, br, and sept, were used to describe signal multiplicities. An FT-IR spectrophotometer (SHIMADZU, IRAffinity-1S) (Shimadzu Corporation, Kyoto, Japan) was used to obtain the infrared spectra. UV absorption measurements were carried out using a Hitachi U-200 double beam spectrophotometer (Hitachi, Ltd., Tokyo, Japan). A Shimadzu LCMS-2020 instrument (Shimadzu Corporation, Kyoto, Japan) was used to perform ESI-MS, and HR-ESI-MS was collected using a VARIAN 901-MS system at National Tsing Hua University, Taiwan. A micro-melting point apparatus (Yanaco MP-500D, Yanaco Technical Science Co., Ltd., Kyoto, Japan) was conducted to measure the melting points. Silica gels (mesh sizes 70–230 and 230–400, Merck KGaA, Darmstadt, Germany) were used for column chromatography. Analytical (0.2 mm) and preparative (0.5 mm) TLC employed silica gel 60 F254 plates.

### 2.2. Plant

The *Garcinia mangostana* L. fruits were purchased in September 2016 from the Shang High-Grade Fruit Shop, located on Jianxing Road, Kaohsiung, Taiwan, and were previously imported as frozen produce from Thailand by Wujia Mu Agricultural Products Co., Ltd. The *G. mangostana* fruits were carefully sliced and air-dried under shade conditions to obtain the dried pericarp, and were identified by Prof. J.-J. Chen, based on the voucher specimen TAI 211661 deposited at the National Taiwan University Herbarium (TAI), Taipei, Taiwan.

### 2.3. Extraction and Isolation

A total of 1.5 kg of dried pericarp of *Garcinia mangostana* L. was extracted 2 times using MeOH. The extracts were concentrated to yield 155 g of crude residue. This crude MeOH extract was subsequently partitioned between an equal volume of ethyl acetate and water, resulting in an EtOAc-soluble fraction (Fraction A, 65 g) and an aqueous layer. The aqueous layer was further subjected to partitioning with an equal volume of *n*-butanol and water, affording a *n*-butanol fraction (Fraction B, 51 g) and a remaining aqueous fraction (Fraction C, 38 g). Fraction A (EtOAc-soluble, 65 g) was subjected to column chromatography, using an elution of *n*-hexane/EtOAc (from 100:0 to 0:100), followed by methanol. This process yielded ten subfractions, labeled fractions A1 to A10. Subsequent purification steps, including preparative thin-layer chromatography (prep. TLC) and MPLC, were applied to individual subfractions to isolate the target compounds.

Fraction A1 (3.2 g) was subjected to purification through column chromatography (CC) with an elution of *n*-hexane/EtOAc (40:1–0:1), yielding 6 subfractions (Fraction A1-1–Fraction A1-6). Part (128 mg) of fraction A1-5 was further purified by prep. TLC (*n*-hexane/CH_2_Cl_2_, 5:1) to obtain 13.2 mg of mixture of β-sitostenone (**20**) and stigmasta-4,22-dien-3-one (**21**) (R*_f_* = 0.39).

Fraction A2 (8.3 g) was subjected to purification through CC with an elution of *n*-hexane/EtOAc (30:1–0:1), yielding 12 subfractions (Fraction A2-1–Fraction A2-12). Fraction A2-5 (125 mg) was further purified by prep. TLC (*n*-hexane/CH_2_Cl_2_, 15:1) to obtain 2.1 mg of garcimangone A (**1**) (R*_f_* = 0.32), 5.3 mg of dulcisxanthone D (**9**) (R*_f_* = 0.53), and 4.3 mg of fuscaxanthone C (**16**) (R*_f_* = 0.39). Fraction A2-8 (485 mg) was purified by MPLC with an elution of *n*-hexane/acetone (25:1–0:1), yielding 9 subfractions (Fraction A2-8-1–Fraction A2-8-9). Fraction A2-8-2 (42 mg) was further purified by prep. TLC (*n*-hexane/EtOAc, 20:1) to obtain 3.2 mg of pruniflorone R (**17**) (R*_f_* = 0.31). Fraction A2-8-4 (57 mg) was further purified by prep. TLC (*n*-hexane/CH_2_Cl_2_, 1:1) to obtain 2.8 mg of garcimangone B (**2**) (R*_f_* = 0.58). Part (158 mg) of fraction A2-10 was further purified by prep. TLC (*n*-hexane/CH_2_Cl_2_, 1:1) to obtain 3.3 mg of brasilixanthone B (**10**) (R*_f_* = 0.52) and 4.1 mg of 8-hydroxycudraxanthone G (**11**) (R*_f_* = 0.48).

Fraction A3 (4.3 g) was purified by CC with a elution of *n*-hexane/acetone (25:1–0:1), yielding 7 subfractions (Fraction A3-1–Fraction A3-7). Fraction A3-2 (274 mg) was purified by prep. TLC (*n*-hexane/acetone, 20:1) to obtain a 10.4 mg mixture of β-sitosterol (**18**) and stigmasterol (**19**) (R*_f_* = 0.21).

Fraction A4 (6.3 g) was purified by CC with an elution of *n*-hexane/acetone (20:1–0:1), yielding 10 subfractions (Fraction A4-1–Fraction A4-10). Fraction A4-3 (553 mg) was further purified by MPLC with an *n*-hexane/EtOAc gradient (15:1–0:1), yielding 10 subfractions (Fraction A4-3-1–Fraction A4-3-10). Fraction A4-3-5 (130 mg) was purified by prep. TLC (*n*-hexane/acetone, 10:1) to yield 4 subfractions (Fraction A4-3-5-1–Fraction A4-3-5-4). Fraction A4-3-5-3 (15 mg) was further purified by HPLC (COSMOSIL 5SL-II packed column (5 mm, 10 mm i.d. × 250 mm); *n*-hexane/CH_2_Cl_2_, 3:7, 2 mL/min) to obtain 5.2 mg of ananixanthone (**14**) (*t*_R_ = 18.63 min) and 6.3 mg of morusignin J (**15**) (*t*_R_ = 20.21 min). Part (189 mg) of fraction 4-7 was purified by prep. TLC (*n*-hexane/acetone, 7:3) to obtain 28.6 mg of α-mangostin (**4**) (R*_f_* = 0.48).

Fraction A5 (4.2 g) was purified by CC with an elution of *n*-hexane/EtOAc (8:1–0:1), yielding 9 subfractions (Fraction A5-1–Fraction A5-9). Fraction A5-3 (387 mg) was further purified by MPLC with an elution of *n*-hexane/acetone (6:1–0:1), yielding 6 subfractions (Fraction A5-3-1–Fraction A5-3-6). Fraction A5-3-3 (28 mg) was purified by prep. TLC (CH_2_Cl_2_/EtOAc, 9:1) to obtain 3.3 mg of tovophyllin A (**12**) (R*_f_* = 0.62). Part (146 mg) of fraction A5-5 was purified by prep. TLC (*n*-hexane/EtOAc, 4:1) to obtain 12.9 mg of β-mangostin (**8**) (R*_f_* = 0.63). Fraction A5-8 (437 mg) was further isolated by MPLC with an elution of *n*-hexane/EtOAc (5:1–0:1), yielding 5 subfractions (Fraction A5-8-1–Fraction A5-8-5). Fraction A5-8-2 (125 mg) was purified by prep. TLC (CH_2_Cl_2_/acetone, 9:1) to obtain 14.8 mg of gartanin (**7**) (R*_f_* = 0.56).

Fraction A6 (3.8 g) was purified by CC with an elution of *n*-hexane/acetone (4:1–0:1), yielding 12 subfractions (Fraction A6-1–Fraction A6-12). Fraction A6-7 (137 mg) was purified by prep. TLC (*n*-hexane/EtOAc, 4:1) to obtain 8.7 mg of garcinone E (**13**) (R*_f_* = 0.55).

Fraction A7 (4.8 g) was purified by CC with an elution of *n*-hexane/acetone (3:1–0:1), yielding 11 subfractions (Fraction A7-1–Fraction A7-11). Part (145 mg) of fraction A7-4 was purified by prep. TLC (CH_2_Cl_2_/methanol, 15:1) to obtain 6.2 mg of γ-mangostin (**5**) (R*_f_* = 0.47). Fraction A7-7 was further isolated by MPLC (*n*-hexane/EtOAc = 2:1) to yield 5 subfractions (Fraction A7-7-1–Fraction A7-7-5). Fraction A7-7-2 (105 mg) was purified by prep. TLC (CH_2_Cl_2_/methanol, 12:1) to obtain 24.3 mg of garcinone D (**6**) (R*_f_* = 0.31). Fraction A7-9 (183 mg) was purified by prep. TLC (CH_2_Cl_2_/methanol, 10:1) to obtain 10.4 mg of garcimangone C (**3**) (R*_f_* = 0.64).

### 2.4. Cell Culture and Reagents

LPS, DMSO, the MTT reagent (3-(4,5-dimethylthiazol-2-yl)-2,5-diphenyltetrazolium bromide), NaNO_2_, andrographolide, curcumin, and sulfanilamide were sourced by Sigma-Aldrich (St. Louis, MO, USA). N-(1-naphthyl)ethylenediamine dihydrochloride (NED) was obtained from both Sigma-Aldrich and ACROS Organics (Geel, Belgium). Phosphoric acid was supplied by Honeywell Fluka (Charlotte, NC, USA). The RAW264.7 macrophage was acquired from the BCRC (Hsinchu, Taiwan). DPBS was obtained from Biological Industries (Foreston, MN, USA), and the Cellbanker 1 cryopreservation solution was sourced from ZENOAQ (Fukushima, Japan). The RAW264.7 cells were cultured in a DMEM medium (#12800-058; Gibco, Carlsbad, CA, USA), supplemented with 10% FBS (Gibco, Carlsbad, CA, USA), 1% PSG (#10378-016; Gibco, Carlsbad, CA, USA) and 1% sodium pyruvate (Gibco #11360-070, Carlsbad, CA, USA). Cells were subcultured in an incubator at 37 °C with a humidified atmosphere and 5% CO_2_.

### 2.5. MTT Assay

Following the reported MTT assay procedure [30], cells (400,000 cells/mL) were cultured in a 96-well plate for 24 h. The cells were then pretreated with the test samples for 1 h, followed by treatment with 100 ng/mL LPS. After 20 h of incubation, the MTT (0.5 mg/mL) reagent was added and incubated for 3 h. Subsequently, DMSO was used to dissolve the formazan crystal, and a microplate reader was used to record the absorbance at 570 nm.

### 2.6. In Vitro Anti-Inflammatory Analysis

Following the reported procedures of Griess assay [31], cells (400,000 cells/mL) were cultured in a 96-well plate for 24 h. Then, the test samples were pretreated for 1 h, followed by 100 ng/mL LPS stimulation for 20 h. Then, the supernatant was reacted with the Griess reagent (composed of 1% sulfanilamide, 0.1% NED, and 5% phosphoric acid) for 15 min. A microplate reader was used to record the absorbance at 550 nm. The NO production levels were quantified through the standard curve established by NaNO_2_.

### 2.7. Evaluation of Inflammatory Cytokine

The production of IL-6 (R&D #DY406-05), TNF-α (R&D #DY410-05), and IL-10 (R&D #DY417-05) was quantified using the commercial ELISA kits (R&D Systems, Minneapolis, MN, USA). Initially, the captured antibody was coated to each well overnight. Subsequently, PBST (PBS with 0.05% Tween-20) was used to wash each well and a 5% non-fat dry milk in PBS was used for blocking for 1 h. After washing again with PBST, either the standard solution or sample was added and allowed to incubate for 2 h. After washing with PBST, the detection antibody was added and incubated for 2 h. After a further wash, the TMB solution was added and incubated for 20 min in the dark. Then, 2 N sulfuric acid was used to terminate the enzymatic reaction. A microplate reader was used to record the absorbance at 450 nm, with 570 nm used as the reference wavelength to correct background noise.

### 2.8. Western Blot Analysis

Following the published procedures [32], cells (1 × 10^6^ cells/plate) were cultured in 60 mm culture dishes for 24 h. The samples were pretreated and incubated with cells for 1 h, and 100 ng/mL of LPS was added for 20 h. After incubation, the cells were rinsed with DPBS and collected by the RIPA lysis buffer for protein extraction. The lysates were centrifuged, collected, and preserved at −80 °C. The protein samples were standardized and heated at 100–110 °C for denaturing. Electrophoresis with SDS-PAGE was used to separate the proteins. Then, a PVDF membrane was used to transfer the protein and blocked with 2% BSA buffer for 1 h. The primary antibodies, including anti-iNOS (Cell Signaling Technology, #13120, Danvers, MA, USA), anti-IκBα (Cell Signaling Technology, #9242, Danvers, MA, USA), anti-phospho-p44/42 MAPK (ERK1/2) (Thr202/Tyr204; Cell Signaling Technology, #9101, Danvers, MA, USA), anti-p44/42 MAPK (ERK1/2; Cell Signaling Technology, #9102, Danvers, MA, USA), anti-phospho-p38 MAPK (Thr180/Tyr182; Cell Signaling Technology, #9216, Danvers, MA, USA), anti-p38 MAPK (Cell Signaling Technology, #9212, Danvers, MA, USA), anti-phospho-SAPK/JNK (Thr183/Tyr185; Cell Signaling Technology, #4668, Danvers, MA, USA), anti-SAPK/JNK (Cell Signaling Technology, #9252, Danvers, MA, USA), anti-arginase 1 (Cell Signaling Technology, #9819, Danvers, MA, USA), anti-KLF4 (Cell Signaling Technology, #4038, Danvers, MA, USA), anti-GAPDH (Cell Signaling Technology, #5174, Danvers, MA, USA), and anti-β-actin (Sigma-Aldrich, #A5441, St. Louis, MO, USA), were further added and incubated for overnight at 4 °C. After washing with TBST, PVDF was incubated with corresponding secondary antibodies for 1 h. After washing with TBST, ECL was used to visualize the target proteins and detected using a imaging system. For reprobing, the membranes were treated with the stripping buffer for 10 min, washed twice with TBST, and then subjected to immunodetection following the same procedure.

### 2.9. Docking Analysis

Following the published procedure [33], the chemical structure with lowest energy conformer was prepared by ChemBioDraw 20.0. Molecular docking was carried out using iGemdock v2.1. The iNOS crystal structure (PDB ID: 1M9T) was obtained from the protein data bank (PDB).

### 2.10. In Silico Physicochemical Properties Prediction

The physicochemical properties were calculated using drug-likeness and a molecular property prediction tool (MolSoft LLC, San Diego, CA, USA; available at: https://molsoft.com/mprop/, accessed on 16 September 2025) [34]. The software estimates these parameters based on the input SMILES or molecular structure files, using pre-trained machine learning models and empirical rules derived from known drug-like molecules. All compound structures were manually curated and input as canonical SMILES strings to ensure consistency and accuracy across calculations.

### 2.11. Statistical Analysis

Analyses were performed using IBM SPSS Statistics software (version 29.0). Statistical comparisons between groups were performed using one-way ANOVA followed by Tukey’s multiple comparison test. Significance levels were denoted as * *p* < 0.05; ** *p* < 0.01; *** *p* < 0.001 compared with the control. Each value represents the mean ± SD from three independent biological experiments (*n* = 3), with each treatment tested in triplicate wells in each experiment.

## 3. Results and Discussion

### 3.1. Compounds Isolation and Purification

MeOH was used to extract the dried pericarp of *Garcinia mangostana* L. (1.5 kg), yielding 155 g of crude extract (GMP-M). GMP-M was further partitioned with ethyl acetate and water, resulting in an EtOAc extract (Fraction A, GMP-EA, 65 g) and an aqueous fraction. The aqueous fraction was further partitioned with an equal volume of *n*-butanol and water to obtain an *n*-butanol extract (Fraction B, GMP-Bu, 51 g) and an aqueous extract (Fraction C, 38 g). Column chromatography (CC) was conducted for the separation of EtOAc extract, which was eluted with *n*-hexane/EtOAc (60:1 to 0:1), followed by MeOH, yielding ten fractions (Fraction A1–A10).

Fractions A1–A7 were further purified using CC, MPLC, prep. TLC, or HPLC, leading to the isolation of multiple components, including β-sitostenone (**20**) and stigmasta-4,22-dien-3-one (**21**) (13.2 mg) from fraction A1; garcimangone A (**1**) (2.1 mg), dulcisxanthone D (**9**) (5.3 mg), fuscaxanthone C (**16**) (4.3 mg), pruniflorone R (**17**) (3.2 mg), garcimangone B (**2**) (2.8 mg), brasilixanthone B (**10**) (3.3 mg), and 8-hydroxycudraxanthone G (**11**) (4.1 mg) from fraction A2; a mixture of β-sitosterol (**18**) and stigmasterol (**19**) (10.4 mg) from fraction A3; ananixanthone (**14**) (5.2 mg), morusignin J (**15**) (6.3 mg), and α-mangostin (**4**) (28.6 mg) from fraction A4; tovophyllin A (**12**) (3.3 mg), β-mangostin (**8**) (12.9 mg), and gartanin (**7**) (14.8 mg) from fraction A5; garcinone E (**13**) (8.7 mg) from fraction A6; and γ-mangostin (**5**) (6.2 mg), garcinone D (**6**) (24.3 mg), and garcimangone C (**3**) (10.4 mg) from fraction A7 (Figure 1 and Figure 2).

### 3.2. Structural Elucidation

**Figure 3:**
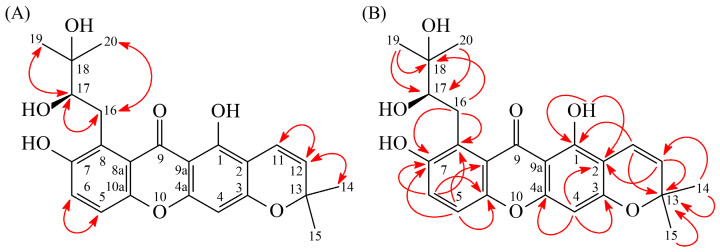
Key ROESY (**A**) and HMBC (**B**) correlation relationship of compound **1**.

Recrystallization of compound **1** was performed with CHCl_3_-MeOH, affording pale yellow needle-shaped crystals (m.p. = 193–195 °C). The ESI-MS spectrum (Figure S1) indicated a pseudomolecular ion peak at *m*/*z* 413 [M+H]^+^. HR-ESI-MS analysis (Figure S2) determined the molecular formula as C_23_H_24_O_7_, with an observed *m*/*z* of 413.2483 [M+H]^+^ (calcd for C_23_H_25_O_7_, 413.1600). According to the IR spectrum (Figure S3), 3447 cm^−1^ and 1651 cm^−1^ indicated a OH group and a C=O group, respectively. The maximum absorptions at 225 (3.88), 287 (4.23), 316 (sh, 3.69), and 391 (3.17) nm were observed in the UV spectrum. The ^1^H-NMR spectra (Figure S4) of compound **1** revealed the presence of a 2,3-dihydroxy-3-methylbutyl moiety at δ 1.27 (3H, s, H-20), 1.38 (3H, s, H-19), 3.65 (1H, dd, *J* = 17.9, 9.0 Hz, H-16), 3.83 (1H, dd, *J* = 17.9, 9.6 Hz, H-16), and 4.77 (1H, d, *J* = 9.6, 9.0 Hz, H-17). Additionally, signals characteristic of a chromeno moiety were observed at δ 1.48 (6H, s, H-14 and H-15), 5.59 (1H, d, *J* = 10.0 Hz, H-12), and 6.73 (1H, d, *J* = 10.0 Hz, H-11). The spectrum also displayed an ortho-coupled aromatic proton pair at δ 7.13 (1H, d, *J* = 8.9 Hz, H-6) and 7.21 (1H, d, *J* = 8.9 Hz, H-5), along with a singlet aromatic proton at δ 6.30 (1H, s, H-4) and a hydrogen-bonded hydroxyl proton at δ 13.16 (1H, s, D_2_O-exchangeable, OH-1). The ^1^H- and ^13^C-NMR (Figures S4 and S5) data closely resembled those of dimethyl–calabaxanthone [35], except for the presence of a 2,3-dihydroxy-3-methylbutyl group at C-8 instead of the isoprenyl group. This substitution was confirmed by HMBC correlations (Figure 3B) between H-16 (δ_H_ 3.65, 3.83) and C-7 (δ_C_ 156.2), C-8 (δ_C_ 125.7), and C-18 (δ_C_ 72.0), as well as ROESY correlations (Figure 3A) between H-16 (δ_H_ 3.65, 3.83) and H-17 (δ_H_ 4.77), H-19 (δ_H_ 1.38), and H-20 (δ_H_ 1.27).

The ROESY and HMBC spectrum were investigated to determine the correlation relationships (Figure 3, Figures S6 and S7). The ROESY correlation (Figure 3A) relationships were observed, including H-5 (δ_H_ 7.21) with H-6 (δ_H_ 7.13); H-11 (δ_H_ 6.73) with H-12 (δ_H_ 5.59); H-12 (δ_H_ 5.59) with H-14/H-15 (δ_H_ 1.48); H-16 (δ_H_ 3.65, 3.83) with H-17 (δ_H_ 4.77); H-19 (δ_H_ 1.38) with H-20 (δ_H_ 1.27); H-17 (δ_H_ 4.77) with H-16 (δ_H_ 3.65, 3.83); and H-19 (δ_H_ 1.38) with H-20 (δ_H_1.27). Additionally, the HMBC correlations (Figure 3B) including OH-1 (δ_H_ 13.16) with C-1 (δ_C_ 157.7), C-2 (δ_C_ 104.1), and C-9a (δ_C_ 104.2); H-4 (δ_H_ 6.30) with C-2 (δ_C_ 104.1), C-3 (δ_C_ 160.7), and C-4a (δ_C_ 156.6); H-5 (δ_H_ 7.21) with C-7 (δ_C_ 156.2), C-8a (δ_C_ 117.9), and C-10a (δ_C_ 151.1); H-6 (δ_H_ 7.13) with C-7 (δ_C_ 156.2), C-8 (δ_C_ 125.7), and C-10a (δ_C_ 151.1); H-11 (δ_H_ 6.73) with C-1 (δ_C_ 157.7), C-13 (δ_C_ 78.2), and C-14/C-15 (δ_C_ 28.4); and H-16 (δ_H_ 3.65, 3.83) with C-7 (δ_C_ 156.2), C-8 (δ_C_ 125.7), and C-18 (δ_C_ 72.0) were observed. On the other hand, the optical rotation of compound **1** was measured as [α]_D_^26^ = +25.2° (c 0.12, CHCl_3_), which was comparable to that of (*R*)-3-methyl-1-phenylbutane-2,3-diol ([α]_D_^23^ = +56°) [36,37] and distinctly different from (*S*)-3-methyl-1-phenylbutane-2,3-diol ([α]_D_^23^ = −55.1°) [36,38]. Based on this comparison, the absolute configuration of C-17 in compound **1** was determined to be in the *R*-form. Furthermore, HSQC (Figure S9) and DEPT (Figure S8) provided confirmation of the structure of compound **1**, and ^1^H-^1^H COSY (Figure S10) supported the ^13^C-NMR data. Based on the above results, compound **1** was identified as garcimangone A, which was confirmed as a new compound.

**Figure 4:**
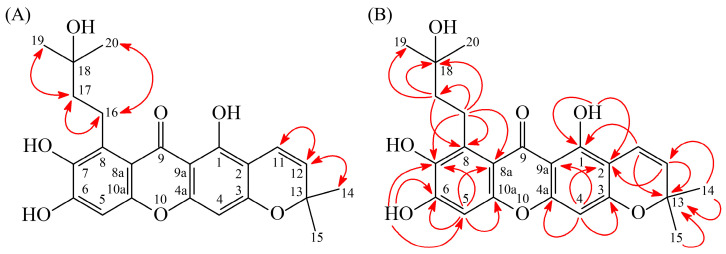
Key ROESY (**A**) and HMBC (**B**) correlation relationship for compound **2**.

Recrystallization of compound **2** was performed with CHCl_3_-MeOH, affording pale-yellow needle-shaped crystals (m.p. = 165–167 °C). The ESI-MS spectrum (Figure S11) indicated a pseudomolecular ion peak at *m*/*z* 413 [M+H]^+^. HR-ESI-MS analysis (Figure S12) determined the molecular formula as C_23_H_24_O_7_, with an observed *m*/*z* of 413.2504 [M+H]^+^ (calcd for C_23_H_25_O_7_, 413.1600). According to the IR spectrum (Figure S13), 3512 and 3429 cm^−1^ indicated OH groups, and 1651 cm^−1^ appeared a C=O group. The maximum absorptions at 220 (3.85), 292 (4.20), 334 (3.70), and 368 (sh, 3.38) nm were observed in the UV spectrum. The ^1^H-NMR spectra (Figure S14) of compound **2** revealed the presence of a 3-hydroxy-3-methylbutyl group at δ 1.39 (6H, s, H-19 and H-20), 1.89 (2H, t, *J* = 6.8 Hz, H-17), and 3.50 (2H, t, *J* = 6.8 Hz, H-16), and a chromeno moiety at δ 1.47 (6H, s, H-14 and H-15), 5.56 (1H, d, *J* = 10.0 Hz, H-12), and 6.73 (1H, d, *J* = 10.0 Hz, H-11). The spectrum also revealed two singlet aromatic proton signals at δ 6.26 (1H, s, H-4) and 6.80 (1H, s, H-6), along with a hydroxyl proton signal at δ 6.40 (1H, s, D_2_O-exchangeable, OH-6) and a hydrogen-bonded hydroxyl proton signal at δ 13.74 (1H, s, D_2_O-exchangeable, OH-1). The ^1^H- and ^13^C-NMR (Figures S14 and S15) data closely resembled those of garcimangos–xanthone F [39], with the primary distinction being the absence of a substituent at C-5. This was evidenced by the presence of a singlet proton signal at δ_H_ 6.81 (1H, s, H-5) and a corresponding carbon signal at δ_C_ 100.5 (C-5). This structural difference was further confirmed by HMBC correlations (Figure 4B), where H-5 (δ_H_ 6.80) exhibited cross-peaks with C-6 (δ_C_ 151.6), C-7 (δ_C_ 138.0), C-8a (δ_C_ 111.3), and C-10a (δ_C_ 153.1), supporting the absence of a substituent at C-5.

The ROESY and HMBC spectrum were investigated to determine the correlation relationships (Figure 4, Figures S16 and S17). ROESY correlation (Figure 4A) relationships were observed, including H-11 (δ_H_ 6.73) with H-12 (δ_H_ 5.56); H-12 (δ_H_ 5.56) with H-11 (δ_H_ 6.73) and H-14/H-15 (δ_H_ 1.47); H-16 (δ_H_ 3.50) with H-17 (δ_H_ 1.89) and H-19/H-20 (δ_H_ 1.39); and H-17 (δ_H_ 1.89) with H-16 (δ_H_ 3.50) and H-19/H-20 (δ_H_ 1.39). Additionally, HMBC correlations (Figure 4B) including OH-1 (δ_H_ 13.74) with C-1 (δ_C_ 157.8), C-2 (δ_C_ 104.3), and C-9a (δ_C_ 103.9); H-4 (δ_H_ 6.26) with C-2 (δ_C_ 104.3), C-3 (δ_C_ 159.6), C-4a (δ_C_ 156.4), and C-9a (δ_C_ 103.9); H-5 (δ_H_ 6.80) with C-6 (δ_C_ 151.6), C-7 (δ_C_ 138.0), C-8a (δ_C_ 111.3), and C-10a (δ_C_ 153.1); OH-6 (δ_H_ 6.40) with C-5 (δ_C_ 100.5), C-6 (δ_C_ 151.6), and C-7 (δ_C_ 138.0); H-11 (δ_H_ 6.73) with C-1 (δ_C_ 157.8) and C-13 (δ_C_ 77.8); H-12 (δ_H_ 5.56) with C-2 (δ_C_ 104.3), C-13 (δ_C_ 77.8) and C-14/C-15 (δ_C_ 28.3); H-16 (δ_H_ 3.50) with C-7 (δ_C_ 138.0), C-8 (δ_C_ 121.3), C-8a (δ_C_ 111.3), C-17 (δ_C_ 32.8), and C-18 (δ_C_ 75.6); and H-17 (δ_H_ 1.89) with C-8 (δ_C_ 121.3), C-18 (δ_C_ 75.6), and C-19/C-20 (δ_C_ 26.5) were observed. Furthermore, HSQC (Figure S19), DEPT (Figure S18), and ^1^H-^1^H COSY (Figure S20) provided confirmation of the structure of compound **2**. Based on the above results, compound **2** was identified as garcimangone B, which was confirmed as a new compound.

**Figure 5:**
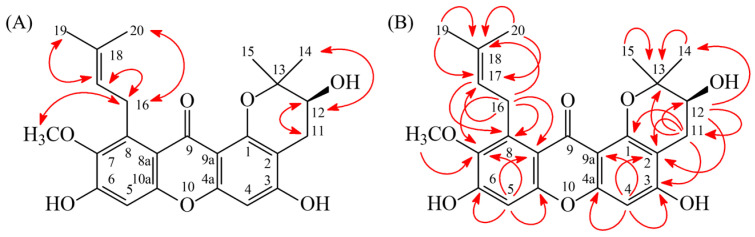
Key ROESY (**A**) and HMBC (**B**) correlation relationship of compound **3**.

Compound **3** was obtained as a yellow viscous substance. The ESI-MS spectrum (Figure S21) indicated a pseudomolecular ion peak at *m*/*z* 449 [M+Na]^+^. HR-ESI-MS analysis (Figure S22) determined the molecular formula as C_24_H_26_O_7_, with an observed *m*/*z* 449.1576 [M+Na]^+^ (calcd for C_24_H_26_NaO_7_, 449.1576). According to the IR spectrum (Figure S23), 3190 cm^−1^ and 1625 cm^−1^ indicated a OH group and a C=O group, respectively. The maximum absorptions at 243 (4.43), 306 (4.21), and 336 (sh, 3.87) nm were observed in the UV spectrum. The ^1^H-NMR spectra (Figure S24) of compound **3** revealed the presence of a (*S*)-2,2-dimethyl-3,4-dihydro-2*H*-pyran-3-ol moiety at δ 1.33 (3H, s, H-14), 1.45 (3H, s, H-15), 2.54 (1H, dd, *J* = 16.9, 7.4 Hz, H_β_-11), 2.90 (1H, dd, *J* = 16.9, 5.6 Hz, H_α_-11), and 3.77 (1H, d, *J* = 7.4, 5.6 Hz, H_β_-12); an isoprenyl group at δ 1.66 (3H, s, H-19), 1.81 (3H, s, H-20), 4.02 (1H, dd, *J* = 13.1, 6.9 Hz, H_a_-16), 4.06 (1H, dd, *J* = 13.1, 6.9 Hz, H_b_-16), and 5.28 (1H, t, *J* = 6.9 Hz, H-17); a methoxy group at δ 3.73 (3H, s, OMe-7); and two singlet aromatic proton signals at δ 6.31 (1H, s, H-4), 6.66 (1H, s, H-5). The ^1^H- and ^13^C-NMR (Figures S24 and S25) data closely resembled those of 12-hydroxy-3-*O*-methyl-1-isomangostin [40], with a hydroxyl (-OH) group substituent at C-3 in compound **3**, instead of a methoxy (-OMe) group. The absence of an ROESY correlation (Figure 5A) between OMe-3 and H-4, which was observed in 12-hydroxy-3-*O*-methyl-1-isomangostin [40], supported the lack of a methoxy group at C-3. Additionally, HMBC correlations (Figure 5B) further validated this substitution, as H-11 (δ_H_ 2.54, 2.90) and H-4 (δ_H_ 6.31) exhibited cross-peaks with C-3 (δ_C_ 162.2), confirming the presence of a hydroxyl group at this position.

The ROESY and HMBC spectrum were investigated to determine the correlation relationships (Figure 4, Figures S26 and S27). ROESY correlation (Figure 5A) relationships were observed, including OMe-7 (δ_H_ 3.73) with H-16 (δ_H_ 4.02, 4.06); H-11 (δ_H_ 2.54, 2.90) with H-12 (δ_H_ 3.77); H-14 (δ_H_ 1.33) with H-12 (δ_H_ 3.77) and H-15 (δ_H_ 1.45); H-16 (δ_H_ 4.02, 4.06) with H-17 (δ_H_ 5.28) and H-19 (δ_H_ 1.66); and H-17 (δ_H_ 5.28) with H-16 (δ_H_ 4.02, 4.06) and H-19 (δ_H_ 1.66). Additionally, HMBC correlations (Figure 5B) including H-4 (δ_H_ 6.31) with C-2 (δ_C_ 105.4), C-3 (δ_C_ 162.2), C-4a (δ_C_ 158.3), and C-9a (δ_C_ 107.6); H-5 (δ_H_ 6.66) with C-6 (δ_C_ 156.7), C-7 (δ_C_ 144.8), C-8a (δ_C_ 114.9), and C-10a (δ_C_ 155.7); OMe-7 (δ_H_ 3.73) with C-7 (δ_C_ 144.8); H-11 (δ_H_ 2.54, 2.90) with C-1 (δ_C_ 156.2), C-2 (δ_C_ 105.4), C-3 (δ_C_ 162.2), C-12 (δ_C_ 69.6), and C-13 (δ_C_ 79.5); H-12 (δ_H_ 3.77) with C-2 (δ_C_ 105.4), C-11 (δ_C_ 27.0), C-14 (δ_C_ 20.6), and C-15 (δ_C_ 25.6); H-14 (δ_H_ 1.33)/ H-15 (δ_H_ 1.45) with C-13 (δ_C_ 79.5); H-16 (δ_H_ 4.02, 4.06) with C-7 (δ_C_ 144.8), C-8 (δ_C_ 138.3), C-8a (δ_C_ 114.9), C-17 (δ_C_ 125.7), and C-18 (δ_C_ 131.4); H-17 (δ_H_ 5.28) with C-8 (δ_C_ 138.3), C-19 (δ_C_ 26.0), and C-20 (δ_C_ 18.3); and H-19 (δ_H_ 1.66)/ H-20 (δ_H_ 1.81) with C-17 (δ_C_ 125.7) and C-18 (δ_C_ 131.4) were observed.

On the other hand, the optical rotation of compound **3** was measured as [α]_D_^26^ = +11.1° (c 0.23, MeOH), which was comparable to that of cochinensoxanthone ([α]_D_^20^ = +11°) [41] and (2*S*)-2-hydroxy-1,2-dihydroacronycine ([α]_D_^20^ = −15.2°) [42], which was distinctly different from (2*R*)-2-hydroxy-1,2-dihydroacronycine ([α]_D_^20^ = −14.9°) [43]. Based on this comparison, the absolute configuration of C-12 in compound **3** was determined to be in the *S*-form. Furthermore, HSQC (Figure S29) and DEPT (Figure S28) provided the confirmation of the structure of compound **3**, and ^1^H-^1^H COSY (Figure S30) and supported the ^13^C-NMR data. While compound **3** with an undefined absolute configuration had previously been isolated from the bark of *Garcinia mangostana* [44], this study marks the first determination of the absolute configuration with an *S*-form. Based on the above results, compound **3** was identified as a new compound with an absolute *S*-form of garcimangone C.

The identification of known compounds **4**–**21**, including α-mangostin (**4**) [43,45], γ-mangostin (**5**) [45,46], garcinone D (**6**) [45], gartanin (**7**) [47], β-mangostin (**8**) [43], dulcisxanthone D (**9**) [48], brasilixanthone B (**10**) [49], 8-hydroxycudraxanthone G (**11**) [50], tovophyllin A (**12**) [51], garcinone E (**13**) [52], ananixanthone (**14**) [53], morusignin J (**15**) [54], fuscaxanthone C (**16**) [55], pruniflorone R (**17**) [56], β-sitosterol (**18**) [57], stigmasterol (**19**) [57], β-sitostenone (**20**) [58], and stigmasta-4,22-dien-3-one (**21**) [58], were performed via ^1^H-NMR, IR, and the ESI-MS spectrum (Figures S31–S76), in comparison with authentic standards or published data.

### 3.3. Anti-Inflammatory Effects of Solvent Extracts

**Figure 6:**
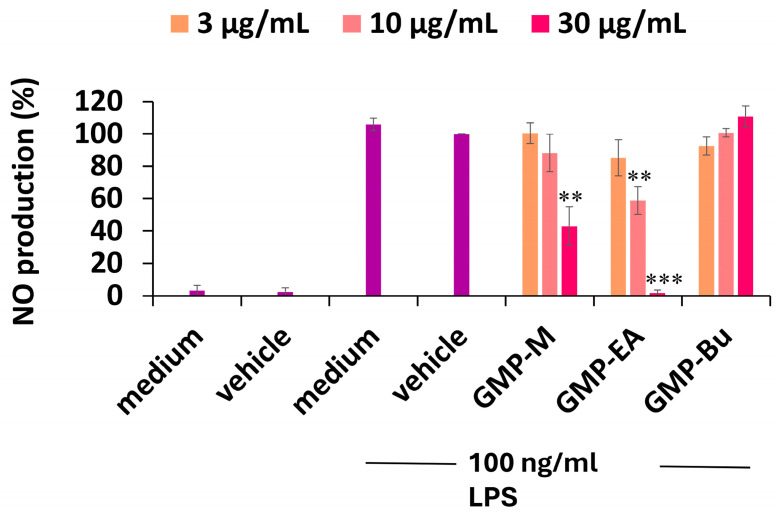
Effects of extracted fractions on LPS-induced NO production. All values were presented as mean ± SD (*n* = 3); ** and *** indicate *p* < 0.01 and *p* < 0.001, respectively, compared to the vehicle control group (LPS group). Purple bars indicate the groups without treatment with the extract samples.

According to the results (Figure 6), treatment with LPS (100 ng/mL) significantly induced NO production compared to the negative control. Interestingly, treatment with different concentrations of GMP-M and GMP-EA significantly inhibited NO production compared to the groups treated with LPS only, whereas GMP-Bu showed non-active in NO inhibition. These findings suggest that the bioactive constituents responsible for NO inhibition are likely concentrated in the ethyl acetate fraction (GMP-EA) of *G. mangostana*.

### 3.4. Anti-Inflammatory Effects of Isolated Compounds

**Figure 7:**
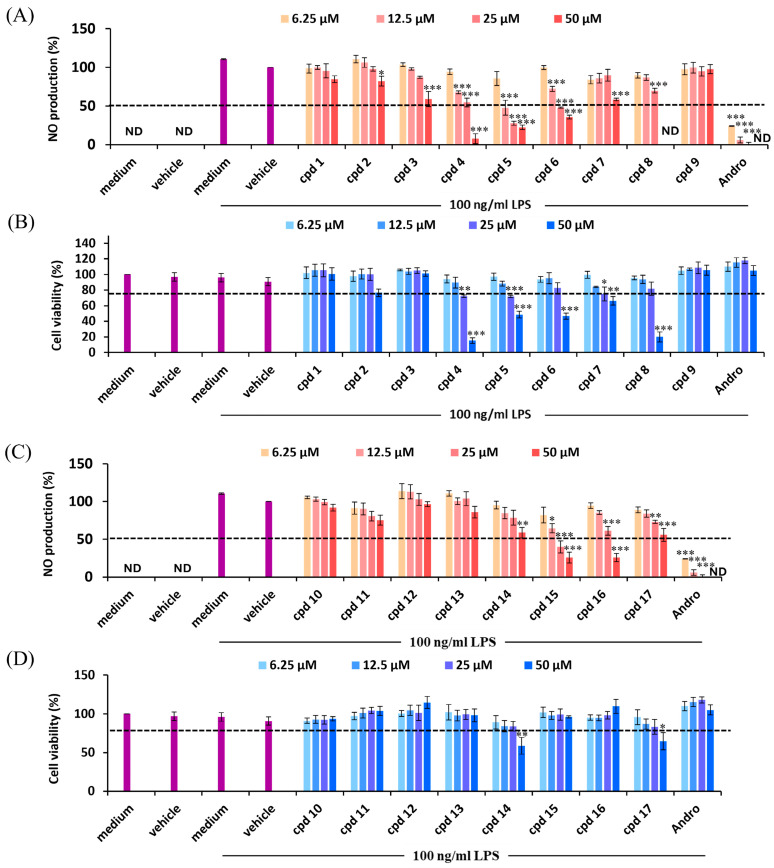
Effects of isolated compounds **1**–**9** on (**A**) LPS-induced NO production and (**B**) cell viability. Effects of compounds **10**–**17** on (**C**) LPS-induced NO production and (**D**) cell viability. All values were presented as mean ± SD (*n* = 3); *, **, and *** indicate *p* < 0.05, *p* < 0.01, and *p* < 0.001, respectively, compared to the vehicle control group (LPS group). Purple bars indicate the groups without compound treatment.

For further investigation of the anti-inflammatory properties of isolated components from *G. mangostana*, the MTT assay and Griess assay were examined for their cytotoxicity and anti-inflammation analysis. Andrographolide was used as a reference compound due to its well-documented effects on anti-inflammation, particularly its inhibition of iNOS and NO in LPS-stimulated RAW264.7 cells [59]. Test concentrations in the range of 6.25–50 μM were initially selected based on preliminary range-finding experiments. As shown in Figure 7, 100 ng/mL LPS significantly induced NO and showed non-cytotoxic effects. On the other hand, Figure 7A,C showed that treatment with different concentrations of isolated components inhibited different levels of NO production. However, the cell viability results (Figure 7B,D) indicated that most of the isolated components possessed good safety (cell viability > 80%), while the compounds **4**–**8**, **14**, and **17** showed cytotoxicity (cell viability < 80%) at 50 μM with a maximum permissible dose (MPD) of 25 μM. In subsequent analyses, the NO-inhibitory effects of each compound were therefore interpreted at non-cytotoxic concentrations within this window, to ensure that the observed reductions in NO production reflected pharmacological activity rather than nonspecific cytotoxicity.

According to Table 1, compounds **5**, **6**, **15**, and **16** exhibited strong inhibition against NO production, with inhibition greater than 55%. Compounds **3**, **4**, **8**, **11**, **14**, and **17** demonstrated moderate NO inhibition, with inhibition ranging from 21% to 45%. In contrast, compounds **1**, **2**, **7**, **9**, **10**, **12**, and **13** exhibited lower activity, with inhibition below 20%.

For the SAR study, the presence and position of functional groups such as chromeno moieties, oxygen-containing substituents (hydroxyl/methoxy groups), and isoprenyl groups appeared to play crucial roles in modulating their anti-inflammatory activity and cytotoxicity (Figure 7 and Table 1). In comparison of compounds **7**, **11**, **14**, and **15**, compounds **14** and **15** with a chromeno moiety at C-3,4 exhibited higher NO inhibition than compounds **7** and **11** without a C-3,4 substituted chromeno moiety. On the other hand, xanthones **4**, **5**, **8**, and **16** with oxygen-containing functional groups at C-1, -3, -6, and -7 position, along with isoprenyl groups at C-2 and -8, exhibited potent NO inhibition, while a difference in cytotoxicity was also observed. Compound **16**, which contains 1-hydroxy and 3,6,7-trimethoxy groups, demonstrated high NO inhibition (74%) and good safety (cell viability > 80%) at 50 μM, whereas compound **4** showed lower cell viability (15%) despite its NO inhibitory activity, indicating potential cytotoxic effects. Additionally, the substituents at C-7 also influenced NO inhibition, as observed in compound **6** and compound **17**, both of which share a 3-hydroxy-3-methylbutyl group at C-8 and an isoprenyl group at C-2. As shown in Table 1, compound **6** exhibited higher NO inhibition (55.20%) than compound **17** (inhibition = 26.95%) at 25 μM, suggesting that the methoxy group at C-7 may enhance NO inhibition, possibly by increasing molecular stability and target affinity, along with the 3-hydroxy-3-methylbutyl group at C-8 and the isoprenyl group at C-2. Furthermore, the higher NO inhibition and lower cytotoxicity of compound **16** than compounds **5**, **6**, and **15** in LPS-induced RAW264.7 cells indicated that fuscaxanthone C (**16**) represents a potent, promising candidate for further investigation.

Overall, these findings suggest that an intact chromeno moiety at C-3,4, the presence of oxygen substituents at C-1,3,6,7, and isoprenyl groups at C-2,8 are key structural features associated with enhanced NO inhibition. Additionally, methoxy substitution at C-7 further contributes to enhancing activity, while cytotoxicity varies among components, emphasizing the need to balance efficacy and safety in drug development. Among the tested components, morusignin J (**15**) and fuscaxanthone C (**16**) exhibited the most promising combination of potent NO suppression and favorable safety in LPS-induced RAW264.7 cells model. Given that excessive NO contributes to the generation of reactive nitrogen species such as peroxynitrite (ONOO^−^), its inhibitory effect on NO production suggests that morusignin J (**15**) and fuscaxanthone C (**16**) may attenuate oxidative stress and downstream free-radical-mediated cellular damage in LPS-induced RAW264.7 cells, supporting their potential as dual-action antioxidants and anti-inflammatory candidates within this in vitro model.

### 3.5. Effects of Morusignin J (***15***) and Fuscaxanthone C (***16***) on Cytokines

**Figure 8:**
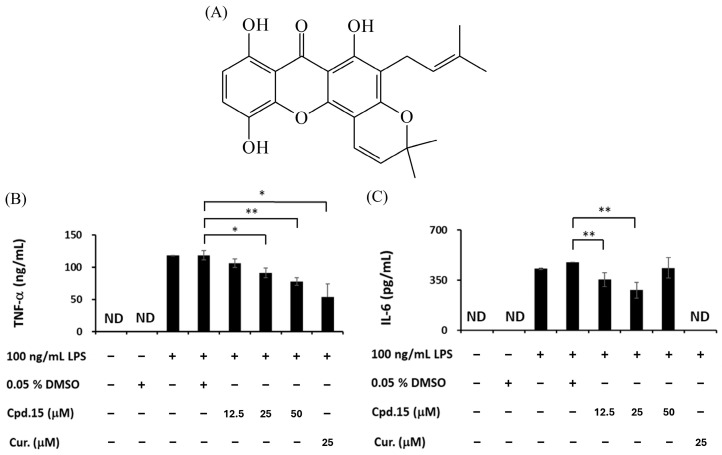
(**A**) The structure of morusignin J (**15**). Effects of compound **15** on (**B**) TNF-α and (**C**) IL-6 levels. * and ** indicate *p* < 0.05 and *p* < 0.01, respectively, compared to the vehicle control group (LPS group).

**Figure 9:**
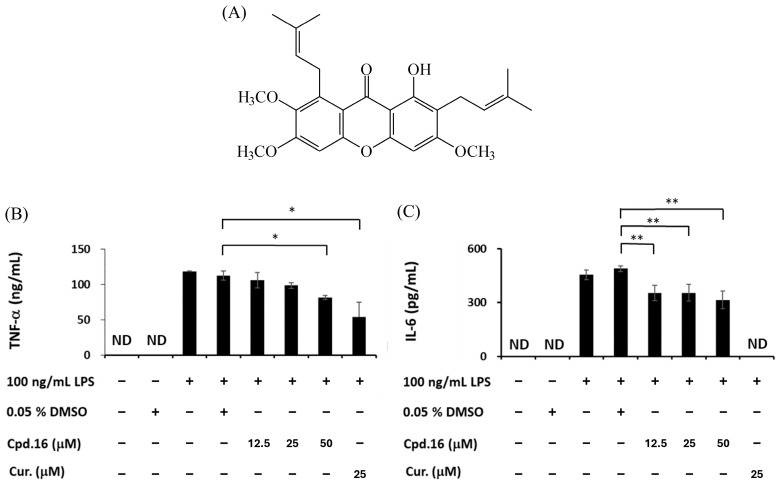
(**A**) The structure of fuscaxanthone C (**16**). Effects of compound **16** on (**B**) TNF-α and (**C**) IL-6 levels. All values are presented as mean ± SD (*n* = 3); * and ** indicate *p* < 0.05 and *p* < 0.01, respectively, compared to the vehicle control group (LPS group).

Furthermore, the potent compounds **15** and **16** with the most favorable safety profiles (cell viability > 80% at 50 μM) were subjected to further LPS-induced inflammatory cytokines analysis. Curcumin was used as a reference compound due to its well-documented anti-inflammatory effects, including inhibition of iNOS and cytokines, as well as the downregulation of IL-10 expression via the modulation of upstream signaling pathways in LPS-induced models [60]. As shown in Figure 8 and Figure 9, 100 ng/mL LPS significantly upregulated the expressions of TNF-α and IL-6, while treatment with compounds **15** and **16** significantly reduced TNF-α and IL-6 at 12.5, 25, and 50 μM dose-dependently.

**Figure 10:**
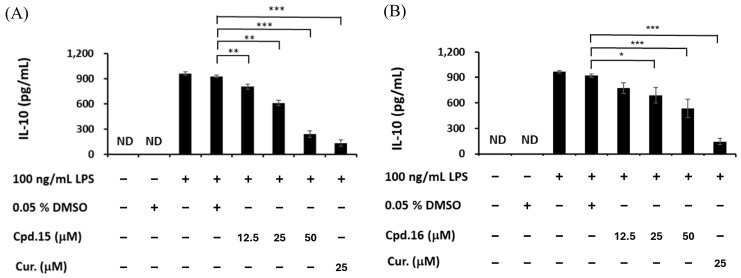
Effects of (**A**) morusignin J (**15**) and (**B**) fuscaxanthone C (**16**) on IL-10 levels. All values are presented as mean ± SD (*n* = 3); *, **, and *** indicate *p* < 0.05, *p* < 0.01, and *p* < 0.001, respectively, compared to the vehicle control group (LPS group).

IL-10, by contrast, serves as an important cytokine involved in maintaining immune homeostasis through the suppression of TNF-α, IL-6, and IL-12 [61]. According to the results (Figure 10), the IL-10 level was significantly increased by 100 ng/mL LPS, indicating an effective induction of negative feedback to counteract inflammation, while the expression of IL-10 was reduced when treating 12.5, 25, and 50 μM of compounds **15** and **16**, suggesting that the negative feedback of inflammatory response was suppressed by inhibiting the upstream inflammatory signaling pathways.

### 3.6. Effects of Morusignin J (***15***) on NF-κB and MAPK Pathways

**Figure 11:**
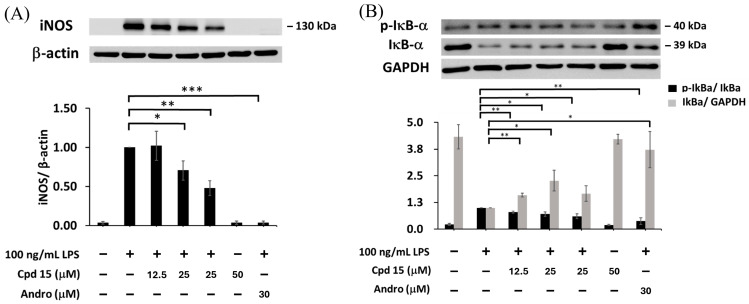
Effects of morusignin J (**15**) on expressions of (**A**) iNOS and (**B**) IκBα. All values are presented as mean ± SD (*n* = 3); *, **, and *** indicate *p* < 0.05, *p* < 0.01, and *p* < 0.001, respectively, compared to the vehicle control group (LPS group).

**Figure 12:**
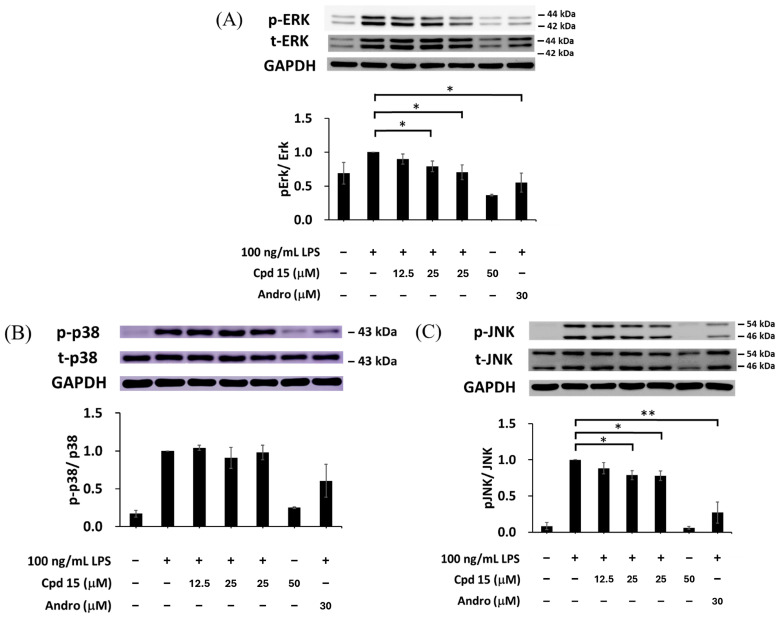
Effects of morusignin J (**15**) on expressions of (**A**) ERK, (**B**) p38, and (**C**) JNK. All values are presented as mean ± SD (*n* = 3); * and ** indicate *p* < 0.05 and *p* < 0.01, respectively, compared to the vehicle control group (LPS group).

In M1 macrophage polarization, several key signaling proteins, including iNOS, IκBα, and MAPKs (ERK, p38, and JNK), play important roles in immune activation and cytokine production [62]. iNOS catalyzes the production of nitric oxide, leading to tissue damage and inflammation. IκBα (inhibitor of NF-κB alpha) prevents the nuclear translocation of NF-κB, thereby suppressing the inflammatory cytokines. MAPKs, including ERK, p38, and JNK, are critical in inflammatory signal transduction. ERK is involved in cell survival and cytokine production; p38 mediates stress responses and cytokine induction; and JNK regulates immune responses and apoptosis. To further study the mechanism of anti-inflammation, morusignin J (**15**) was subjected to Western blot analysis (Figure 11 and Figure 12). Andrographolide was employed as a positive control, owing to its well-established anti-inflammatory activity, especially its capacity to inhibit the MAPK signaling pathway [63] and its ability to promote M2 polarization of macrophages in inflammatory models [64].

According to the results (Figure 11), LPS (100 ng/mL) increased the iNOS expression and phosphorylation of IκBα significantly, leading to NO production, a decrease in IκBα, and an inflammatory response. Treatment with morusignin J (**15**) reduced iNOS expression to 1.03 ± 0.20, 0.77 ± 0.11, and 0.46 ± 0.06-fold at 12.5, 25, and 50 μM, respectively, compared to the LPS group (Figure 11A). On the other hand, p-IκBα expression was reduced to 0.79 ± 0.05-, 0.70 ± 0.10-, and 0.60 ± 0.11-fold of the LPS group, respectively (Figure 11B). Additionally, the IκBα expression of LPS (100 ng/mL) group was decreased by 0.23 ± 0.56-fold compared to the negative control. Importantly, morusignin J (**15**) significantly increased IκBα levels by 1.59 ± 0.01, 2.27 ± 0.49, and 1.66 ± 0.37-fold compared to the LPS group at 12.5, 25, and 50 μM, respectively (Figure 11B).

As shown in Figure 12, treatment with 12.5, 25, and 50 μM of morusignin J (**15**) inhibited p-ERK expression by 0.94 ± 0.08, 0.82 ± 0.07, and 0.74 ± 0.10-fold (Figure 12A), respectively, and reduced p-JNK expression by 0.93 ± 0.06, 0.85 ± 0.01, and 0.82 ± 0.02-fold (Figure 12C), respectively, while it showed no significant effect on p38 phosphorylation (Figure 12B). Taken together, these findings indicate that morusignin J (**15**) attenuates LPS-induced activation of iNOS, NF-κB, and MAPKs signaling in RAW264.7 macrophages, thereby dampening downstream inflammatory responses in this in vitro model.

### 3.7. Effects of Morusignin J (***15***) on M2-Associated Markers

KLF4 (Kruppel-like factor 4) is a key transcription factor that regulates M2 macrophage polarization, playing a crucial role in suppressing pro-inflammatory genes while promoting anti-inflammatory pathways, facilitating tissue repair and immune homeostasis [65]. On the other hand, arginase 1 (Arg1) is a hallmark enzyme of M2 macrophages, catalyzing the conversion of L-arginine to ornithine and urea and competing with iNOS for L-arginine, thereby suppressing inflammation and promoting wound healing and tissue repair [65].

**Figure 13:**
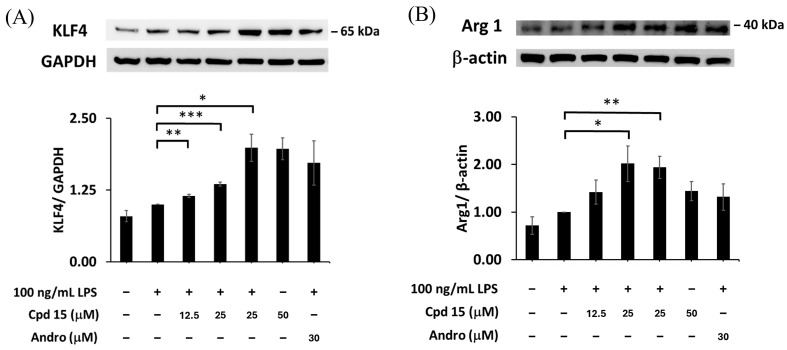
Effects of morusignin J (**15**) on expressions of (**A**) KLF4 and (**B**) arginase 1. All values are presented as mean ± SD (*n* = 3); *, **, *** indicate *p* < 0.05, *p* < 0.01, and *p* < 0.001, respectively, compared to the vehicle control group (LPS group).

According to the results in Figure 13, morusignin J (**15**) significantly induced KLF4 expression, increasing by 13%, 28%, and 38%, at 12.5, 25, and 50 μM, respectively, compared to the LPS group (Figure 13A). Furthermore, the expression of arginase 1 was also enhanced by 1.28 ± 0.26, 1.92 ± 0.42, and 1.80 ± 0.38-fold relative to the LPS group upon treatment with morusignin J (**15**) at 12.5, 25, and 50 μM (Figure 13B), respectively. Within the context of LPS-induced RAW264.7 macrophages, these changes in M2-associated markers are consistent with a partial shift toward an M2-like, pro-resolving phenotype, which may contribute to the overall anti-inflammatory profile of morusignin J (**15**).

Based on these findings, morusignin J (**15**) not only attenuates the inflammatory response by down-regulating iNOS and NF-κB/MAPK activation, but also enhances the expression of M2-associated markers such as KLF4 and arginase 1. Given the interplay between oxidative stress and inflammation, this dual action suggests that morusignin J (**15**) may contribute to limiting nitrosative stress and promote resolution-oriented responses in this in vitro system, and warrants further investigation.

### 3.8. Molecular Docking

To investigate the potential interactions of bioactive compounds with iNOS, morusignin J (**15**) was first subjected to the molecular docking analysis. According to the results presented in Table 2, morusignin J (**15**) (total affinity score = −131.68) showed a favorable predicted binding affinity to iNOS. Notably, morusignin J (**15**) (H-bond affinity score = −48.4) exhibited a comparable hydrogen bond interaction score to that of andrographolide (H-bond affinity score = −48.6), engaging various important residues within the iNOS binding site, including Gln257, Pro344, Glu371, and Hem901, which are crucial for ligand binding and enzymatic regulation. Additionally, binding with Gln257 has also been reported to stabilize the catalytic pocket [66]. These predicted interactions are consistent with the iNOS-related inhibitory effects and reinforce the proposed mechanism of morusignin J (**15**) against iNOS.

**Figure 14:**
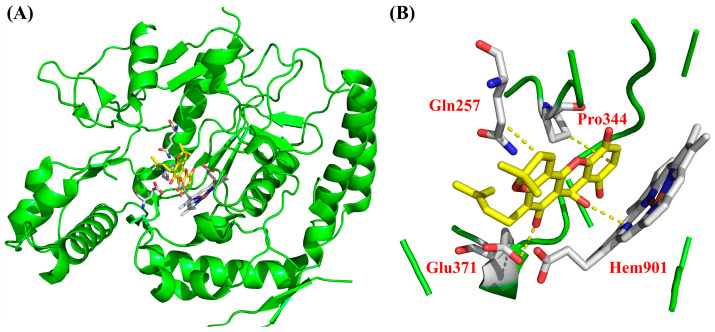
The binding interaction of morusignin J (**15**) with (**A**) iNOS and (**B**) active binding site. Yellow dotted lines indicate the key interactions between morusignin J (**15**) and the active-site residues of iNOS.

To further elucidate the molecular basis of the iNOS inhibitory activity of morusignin J (**15**), the binding pose within the iNOS catalytic pocket was analyzed and visualized, as shown in Figure 14. According to Figure 14, the docking results revealed that morusignin J (**15**) was well-accommodated in the active site of iNOS, aligning along the catalytic cavity and occupying a similar spatial region as the positive control, andrographolide. Specifically, morusignin J (**15**) formed several key van der Waals (VDW) interactions with amino acid residues including Gln257, Glu371, and Pro344, which are known to contribute to substrate positioning and stabilization. Notably, the compound also established VDW and hydrogen-bonding interactions with the heme prosthetic group (Hem901), a central cofactor involved in iNOS catalytic function. These interaction patterns suggest that morusignin J (**15**) may exert its inhibitory effect by occupying the catalytic pocket and engaging residues and cofactors critical for enzymatic activity. The favorable binding interactions of morusignin J (**15**) with iNOS thus provide a plausible structural basis for its observed iNOS and NO inhibition in LPS-induced RAW264.7 cells and support its potential value for further investigation as a natural iNOS-targeting anti-inflammatory candidate.

**Figure 15:**
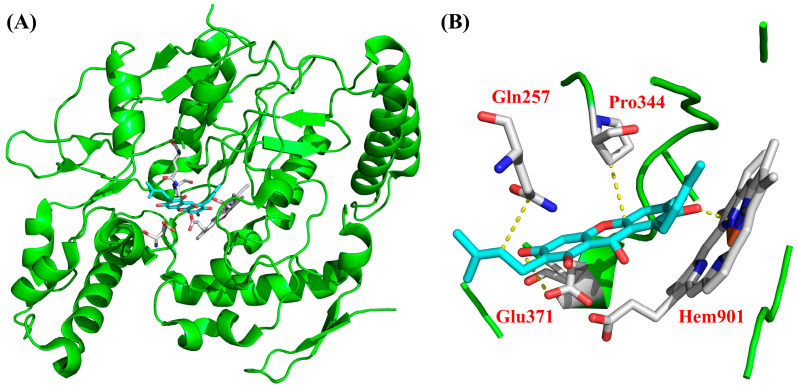
The binding interaction of α-mangostin (**5**) with (**A**) iNOS and (**B**) active binding site. Yellow dotted lines indicate the key interactions between α-mangostin (**5**) and the active-site residues of iNOS.

**Figure 16:**
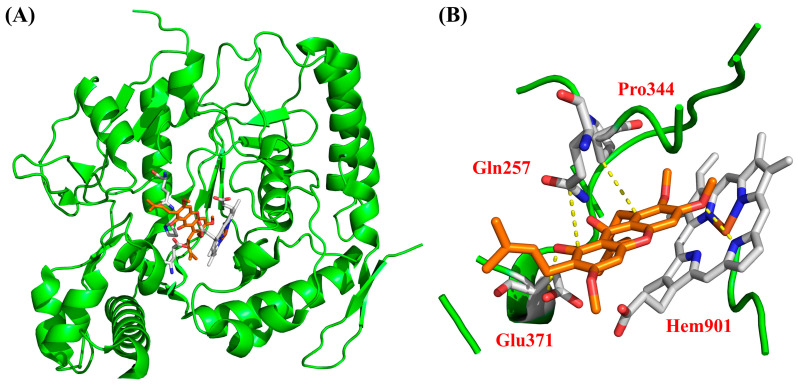
The binding interaction of fuscaxanthone C (**16**) with (**A**) iNOS and (**B**) active binding site. Yellow dotted lines indicate the key predicted interactions between fuscaxanthone C (**16**) and the active-site residues of iNOS.

To further explore how structural differences among prenylated xanthones may influence iNOS binding, α-mangostin (**5**) and fuscaxanthone C (**16**), which exhibited NO inhibitory activity under non-cytotoxic conditions, were additionally subjected to molecular docking analysis (Table 2, Figure 15 and Figure 16).

According to Figure 15 and Table 2, α-mangostin (**5**) exhibited favorable docking affinity (total affinity score = −154.43). Its binding pose extended along the iNOS catalytic tunnel and occupied the heme-containing region, where α-mangostin (**5**) formed an extensive hydrogen-bonding network (total H-bond affinity score = −49.2) and pronounced VDW contacts (VDW affinity score = −105.23). In particular, α-mangostin (**5**) engaged Gln257, Pro344, and Glu371 through VDW interactions and showed strong contact with Hem901, which was considered important for substrate positioning and stabilization within the catalytic pocket. These docking features were consistent with its significant NO inhibitory activity under non-cytotoxic concentrations, suggesting that α-mangostin (**5**) may stabilize the active-site environment of iNOS in a manner comparable to andrographolide.

As shown in Figure 16 and Table 2, fuscaxanthone C (**16**) also displayed a favorable predicted binding affinity toward iNOS (total affinity score = −135.59), with VDW interactions (affinity score = −102.29) contributing more prominently than hydrogen bonding (affinity score = −33.3). The docking pose revealed that fuscaxanthone C (**16**) occupied a similar catalytic region, establishing key contacts with Gln257, Pro344, and Glu371. Notably, fuscaxanthone C (**16**) exhibited relatively strong VDW interactions with Gln257 and Glu371 (affinity score = −7.6 and −8.4, respectively), together with hydrophobic and hydrogen-bonding contacts with Hem901 (H-bond affinity score = −25.7; VDW affinity score = −36.7), indicating that the prenylated moiety is oriented toward the heme pocket.

Although the total docking scores differed among α-mangostin (**5**), morusignin J (**15**), and fuscaxanthone C (**16**), their predicted interaction patterns largely preserved the key contacts identified for morusignin J (**15**) and the reference inhibitor andrographolide, including engagement with Hem901 and the surrounding residues Gln257, Pro344, and Glu371 within the catalytic pocket. Comparing together with the NO inhibition profiles in Table 1, they suggest a trend in which prenylated xanthones that are able to sustain favorable VDW and H-bond interactions in the heme-proximal region of iNOS and that possess appropriately oxygenated xanthone cores with prenyl side chains tend to exhibit more pronounced NO inhibitory activity at non-cytotoxic concentrations.

Taken together, the docking profiles of α-mangostin (**5**), morusignin J (**15**), and fuscaxanthone C (**16**), while not providing direct experimental evidence of iNOS modulation for compounds **5** and **16**, outline a plausible structural framework in which preserved heme-adjacent contacts may contribute to the iNOS- and NO-inhibitory potential of these prenylated xanthones, thereby providing a useful basis for the future design of analogs and for QSAR modeling.

### 3.9. In Silico Prediction of Physicochemical Properties

The physicochemical properties predictions were further conducted to extend the applications of the bioactive α-mangostin (**5**), morusignin J (**15**), and fuscaxanthone C (**16**). The physicochemical and pharmacokinetic properties, including lipophilicity (LogP), molecular weight (M.W.), numbers of hydrogen bond donors (HBDs) and acceptors (HBAs), drug-likeness score, blood–brain barrier (BBB) permeability, and polar surface area (PSA), were evaluated and are summarized in Table 3. The analysis of Lipinski’s rule of five identified any violations related to drug-likeness criteria. The drug-likeness index was used to assess the likelihood of a compound being further developed into a viable drug candidate [67]. Moreover, the estimated oral absorption percentage (%ABS) was calculated to predict the compounds’ potential oral bioavailability [34].

According to the results (Table 3), the bioactive prenylated xanthones, including α-mangostin (**5**), morusignin J (**15**), and fuscaxanthone C (**16**), exhibited the favorable physicochemical characteristics of M.W. (≤500), HBA (≤10), and HBD (≤5). Although its Lipinski’s rule violation score was 1 due to a LogP value greater than 5, the drug-likeness scores of mangostin (**5**), morusignin J (**15**), and fuscaxanthone C (**16**), which were closer to 1 compared to andrographolide, suggested a potentially more favorable profile for developing oral application [67]. On the other hand, the predicted PSA and %ABS results indicated good predicted oral bioavailability, as evidenced by PSA values below 140 Å^2^ and %ABS values above 63% [68]. In addition, the predicted BBB penetration scores of ≤4 implied that mangostin (**5**), morusignin J (**15**), and fuscaxanthone C (**16**) were unlikely to cross into the central nervous system, suggesting they may possess favorable profile for peripheral or non-CNS-related applications [69]. Taken together, these in silico predictions support the argument that α-mangostin (**5**), morusignin J (**15**), and fuscaxanthone C (**16**) possess physicochemical properties compatible with further development as oral candidates, while also highlighting that future optimization may focus on fine-tuning lipophilicity to improve solubility.

Based on the above findings, morusignin J (**15**) not only exhibits robust anti-inflammatory activity in LPS-induced RAW264.7 macrophages through immunomodulatory signaling and macrophage phenotype shifts, but also demonstrates favorable binding affinity and physicochemical properties indicative of potential systemic bioavailability. Its ability to modulate nitric oxide levels and suppress iNOS expression further implies an indirect antioxidant effect via reactive nitrogen species regulation. Although the present work focused on M1- and M2-associated markers within a single LPS-induced RAW264.7 macrophage model, and the findings were obtained exclusively from this in vitro model, together with in silico physicochemical evaluations. These results offer a new insight into the promising potential of morusignin J (**15**) and other bioactive extracts from the pericarps of *G. mangostana* as natural sources for antioxidative and anti-inflammatory candidates, warranting further mechanistic and in vivo investigation to fully characterize macrophage polarization in the future.

## 4. Conclusions

In this study, three new compounds, including garcimangone A (**1**), garcimangone B (**2**), and the *S*-form of garcimangone C (**3**), and 18 known compounds were successfully isolated and characterized from *G. mangostana* pericarp. The bioactive extracts and components were investigated for their anti-inflammatory activities, demonstrating their effects on LPS-induced NO production. Among the isolated bioactive components, γ-mangostin (**5**), garcinone D, morusignin J (**15**), and fuscaxanthone C (**16**) showed strong NO-inhibitory effects. SAR study revealed that the chromeno moiety at C-3,4, oxygen substituents at C-1,3,6,7, and isoprenyl groups at C-2,8 are key structural features enhancing NO inhibition. Cytokine analysis results further indicated that morusignin J (**15**) and fuscaxanthone C (**16**) effectively modulated TNF-α and IL-6. Additionally, Western blot results demonstrated that morusignin J (**15**) modulated the inflammatory response through NF-κB and MAPK signaling and increased the expression of M2-associated markers KLF4 and arginase-1 in LPS-induced RAW264.7 macrophages, contributing to the anti-inflammatory effects. Further molecular docking analysis revealed that morusignin J (**15**) and fuscaxanthone C (**16**) exhibited favorable binding affinities with important residues, including Gln257, Pro344, Glu371, and Hem901. In addition, predicted physicochemical properties supported its oral bioavailability and drug-likeness, suggesting its potential as an orally available promising candidate. These in vitro and in silico findings highlight that pericarps of *G. mangostana* possess the potential as promising natural sources for bioactive extracts and constituents, particularly morusignin J (**15**), offering new insights into their application in the development of antioxidant and anti-inflammatory candidates, and they clearly warrant additional mechanistic and in vivo evaluation in the future.

## Figures and Tables

**Table 1:** Effects of *Garcinia mangostana* components on NO production.

Compound	Dosage (μM)	NO Inhibition (%)
Garcimangone A (**1**)	50	15.39 ± 4.23
Garcimangone B (**2**)		17.94 ± 6.20 *
Garcimangone C (**3**)		40.82 ± 9.78 ***
α-mangostin (**4**)	25	45.39 ± 5.64 ***
γ-mangostin (**5**)		72.32 ± 2.68 ***
Garcinone D (**6**)		55.20 ± 0.70 ***
Gartanin (**7**)		10.23 ± 7.69
β-mangostin (**8**)		30.26 ± 3.04 ***
Dulcisxanthone D (**9**)		5.18 ± 6.16
Brasilixanthone B (**10**)	50	8.01 ± 4.38
8-hydroxycudraxanthone G (**11**)		24.61 ± 6.52
Tovophyllin A (**12**)		3.50 ± 3.28
Garcinone E (**13**)		14.03 ± 7.57
Ananixanthone (**14**)	25	21.42 ± 10.02
Morusignin J (**15**)	50	74.03 ± 7.09 ***
Fuscaxanthone C (**16**)		74.14 ± 5.53 ***
Pruniflorone R (**17**)	25	26.95 ± 2.14 ***
Andrographolide		99.59 ± 2.42 ***

All values are presented as mean ± SD (*n* = 3); * and *** indicate *p* < 0.05 and *p* < 0.001, respectively, compared to the vehicle control group (LPS group).

**Table 2:** Binding affinity of compounds **5**, **15**, and **16** with the iNOS catalytic pocket.

Binding Affinity	Residue	Cpd 5 ^c^	Cpd 15 ^c^	Cpd 16 ^c^	Andrographolide ^d^
Total		−154.43	−131.68	−135.59	−163.47
H-bond ^a^	Total	−49.2	−48.4	−33.3	−48.6
	Glu371	−2.5	−3.6	−2.4	−3.4
	Hem901	−33.9	−28	−25.7	−30
VDW ^b^	Total	−105.23	−83.27	−102.29	−114.87
	Gln257	−4.9	−3.7	−7.6	−7.3
	Pro344	−6.6	−7.3	−5.6	−2.3
	Glu371	−4.9	−4.8	−8.4	−5.2
	Hem901	−48.1	−26.4	−36.7	−52

^a^ H-bond indicates hydrogen bond interactions. ^b^ VDW indicates van der Waals interactions. ^c^ Cpd indicates compound. ^d^ Andrographolide was used as a positive control.

**Table 3:** In silico physicochemical property prediction of compounds **5**, **15**, and **16** and andrographolide.

Compound	LogP	M.W.	HBA	HBD	Lipinski’s Violation	Drug-Likeness Score	BBB ^a^	PSA (Å^2^)	%ABS ^b^
α-mangostin (**5**)	5.99	396.16	6	4	1	0.18	2.61	84.25	79.93
Morusignin J (**15**)	5.61	394.41	6	3	1	−0.03	2.75	74.16	83.41
Fuscaxanthone C (**16**)	7.15	438.20	6	1	1	0.25	3.66	58.66	88.76
Andrographolide	2.19	350.21	5	3	0	−0.64	2.26	71.27	84.41

^a^ BBB scores (0–6): 0 indicates the weakest permeability and 6 the highest; BBB ≤ 4 suggest suitability for non-CNS drugs. ^b^ %ABS = 109 − 0.345 × PSA.

## Data Availability

The original contributions presented in this study are included in the article/Supplementary Material. Further inquiries can be directed to the corresponding author.

## Supplementary Materials

The following supporting information can be downloaded at https://www.mdpi.com/article/10.3390/antiox15010128/s1, Figure S1–S10: MS, IR, and NMR spectrum of compound **1**; Figure S11–S20: MS, IR, and NMR spectrum of compound **2**; Figure S21–S30: MS, IR, and NMR spectrum of compound **3**; Figure S31–S33: MS, IR, and NMR spectrum of compound **4**; Figure S34–S36: MS, IR, and NMR spectrum of compound **5**; Figure S37–S39 MS, IR, and NMR spectrum of compound **6**; Figure S40–S42: MS, IR, and NMR spectrum of compound **7**; Figure S43–S45: MS, IR, and NMR spectrum of compound **8**; Figure S46–S48: MS, IR, and NMR spectrum of compound **9**; Figure S49–S51: MS, IR, and NMR spectrum of compound **10**; Figure S52–S54: MS, IR, and NMR spectrum of compound **11**; Figure S55–S57: MS, IR, and NMR spectrum of compound **12**; Figure S58–S60: MS, IR, and NMR spectrum of compound **13**; Figure S61–S63: MS, IR, and NMR spectrum of compound **14**; Figure S64–S66: MS, IR, and NMR spectrum of compound **15**; Figure S67–S69: MS, IR, and NMR spectrum of compound **16**; Figure S70–S72: MS, IR, and NMR spectrum of compound **17**; Figure S73 and S74: IR and NMR spectrum of compound **18** and **19**; Figure S75 and S76: IR and NMR spectrum of compound **20** and **21**.
