# Supplementary material for: Insights into Bioactive Constituents from Pericarp of Garcinia mangostana: Anti-Inflammatory Effects via NF-κB/MAPK Modulation and M1/M2 Macrophage Polarization
Source: Antioxidants (Basel). 2026 Jan 19;15(1):128. doi: 10.3390/antiox15010128 (PMC12838342; doi:10.3390/antiox15010128)
Supplement: Supplementary file 1 [file antioxidants-15-00128-s001.zip › antioxidants-4042398-supplementary.pdf]

Supporting Information

Insights of Bioactive Constituents from Pericarp of *Garcinia mangostana*: Anti-Inflammatory Effects via NF-κB/MAPK Modulation and M1/M2 Macrophage Polarization

Cheng-Shin Yang [1], Sin-Min Li [2], and Jih-Jung Chen [1,2,3,4,*]

[1] Institute of Biopharmaceutical Sciences, College of Pharmaceutical Sciences, National Yang Ming Chiao Tung University, Taipei 112304, Taiwan

[2] Department of Pharmacy, School of Pharmaceutical Sciences, National Yang Ming Chiao Tung University, Taipei 112304, Taiwan

[3] Department of Medical Research, China Medical University Hospital, China Medical University, Taichung 404333, Taiwan

[4] Traditional Herbal Medicine Research Center, Taipei Medical University Hospital, Taipei 110301, Taiwan

* Corresponding author

E-mail address: jjungchen@nycu.edu.tw Tel.: +886-2-2826-7195

Contents

Spectrum data of compounds…S3

MS, IR, UV, and NMR spectrum of isolated compound **1**…S7

MS, IR, UV, and NMR spectrum of isolated compound **2**…S11

MS, IR, UV, and NMR spectrum of isolated compound **3**…S16

MS, IR, and NMR spectrum of isolated compound **4**…S21

MS, IR, and NMR spectrum of isolated compound **5**…S22

MS, IR, and NMR spectrum of isolated compound **6**…S23

MS, IR, and NMR spectrum of isolated compound **7**…S25

MS, IR, and NMR spectrum of isolated compound **8**…S26

MS, IR, and NMR spectrum of isolated compound **9**…S28

MS, IR, and NMR spectrum of isolated compound **10**…S29

MS, IR, and NMR spectrum of isolated compound **11**…S31

MS, IR, and NMR spectrum of isolated compound **12**…S32

MS, IR, and NMR spectrum of isolated compound **13**…S34

MS, IR, and NMR spectrum of isolated compound **14**…S35

MS, IR, and NMR spectrum of isolated compound **15**…S37

MS, IR, and NMR spectrum of isolated compound **16**…S38

MS, IR, and NMR spectrum of isolated compound **17**…S40

IR, and NMR spectrum of isolated compound **18** and **19**…S41

IR, and NMR spectrum of isolated compound **20** and **21**…S42

Spectrum data of isolated compounds

Garcimangone A (1)

Yellowish needles ($CHCl_3$-MeOH); mp 193–195 °C; $[\alpha]_D^{26}$ = +25.2° (*c* 0.12, $CHCl_3$); UV (MeOH) λ_{max} (log ε) 225 (3.88), 287 (4.23), 316 (sh, 3.69), 391 (3.17) nm; IR (neat) ν_{max} 3347 (OH), 1651 (C=O) cm^{-1}; ^{1}H-NMR (500 MHz, $CDCl_3$) δ 1.27 (3H, s, H-20), 1.38 (3H, s, H-19), 1.48 (6H, s, H-14 and H-15), 3.65 (1H, dd, *J* = 17.9, 9.0 Hz, H-16), 3.83 (1H, dd, *J* = 17.9, 9.6 Hz, H-16), 4.77 (1H, d, *J* = 9.6, 9.0 Hz, H-17), 5.59 (1H, d, *J* = 10.0 Hz, H-12), 6.30 (1H, s, H-4), 6.73 (1H, d, *J* = 10.0 Hz, H-16), 7.13 (1H, d, *J* = 8.9 Hz, H-6), 7.21 (1H, d, *J* = 8.9 Hz, H-5), 13.16 (1H, s, D_2O exchangeable, OH-1); ^{13}C-NMR (125 MHz, $CDCl_3$) δ 23.8 (C-20), 26.2 (C-19), 28.4 (C-14 and C-15), 32.4 (C-16), 72.0 (C-18), 78.2 (C-13), 91.0 (C-17), 94.8 (C-4), 104.1 (C-2), 104.2 (C-9a), 115.5 (C-11), 116.0 (C-6), 117.1 (C-5), 117.9 (C-8a), 125.7 (C-8), 127.4 (C-12), 151.1 (C-10a), 156.2 (C-7), 156.6 (C-4a), 157.7 (C-1), 160.7 (C-3), 183.3 (C-9); ESI-MS *m/z* 413 $[M+H]^+$; HR-ESI-MS *m/z* 413.24833 $[M+H]^+$ (calcd for $C_{23}H_{25}O_7$, 413.16003).

Garcimangone B (2)

Yellowish needles ($CHCl_3$-MeOH); mp 165–167 °C; UV (MeOH) λ_{max} (log ε) 220 (3.85), 292 (4.20), 334 (3.70), 368 (sh, 3.38) nm; IR (neat) ν_{max} 3512, 3429 (OH), 1651 (C=O) cm^{-1}; ^{1}H-NMR (500 MHz, $CDCl_3$) δ 1.39 (6H, s, H-19 and H-20), 1.47 (6H, s, H-14 and H-15), 1.89 (2H, t, *J* = 6.8 Hz, H-17), 3.50 (2H, t, *J* = 6.8 Hz, H-16), 5.56 (1H, d, *J* = 10.0 Hz, H-12), 6.26 (1H, s, H-4), 6.73 (1H, d, *J* = 10.0 Hz, H-16), 6.40 (1H, s, D_2O exchangeable, OH-6), 6.80 (1H, s, H-6), 13.74 (1H, s, D_2O exchangeable, OH-1); ^{13}C-NMR (125 MHz, $CDCl_3$) δ 22.3 (C-16), 26.5 (C-19, C-20), 28.3 (C-14, C-15), 32.8 (C-17), 75.6 (C-18), 77.8 (C-13), 94.1 (C-4), 100.5 (C-5), 103.9 (C-9a), 104.3 (C-2), 111.3 (C-8a), 115.7 (C-11), 121.3 (C-8), 127.1 (C-12), 138.0 (C-7), 151.6 (C-10a), 153.1 (C-4a), 156.4 (C-4a), 157.8 (C-1), 159.6 (C-3), 182.6 (C-9); ESI-MS *m/z* 413 $[M+H]^+$; HR-ESI-MS *m/z* 413.25044 $[M+H]^+$ (calcd for $C_{23}H_{25}O_7$, 413.16003).

Garcimangone C (3)

Yellow gum; $[\alpha]_D^{26}$ = +11.1° (*c* 0.23, MeOH); UV (MeOH) λ_{max} (log ε) 243 (4.43), 306 (4.21), 336 (sh, 3.87) nm; IR (neat) ν_{max} 3190 (OH), 1625 (C=O) cm^{-1}; ^{1}H-NMR (500 MHz, $CDCl_3$) δ 1.33 (3H, s, H-14), 1.45 (3H, s, H-15), 1.66 (3H, s, H-19), 1.81 (3H, s, H-20), 2.54 (1H, dd, *J* = 16.9, 7.4 Hz, H-11), 2.90 (1H, dd, *J* = 16.9, 5.6 Hz, H-11), 3.73 (3H, s, OMe-7), 3.77 (1H, d, *J* = 7.35, 5.65 Hz, H-12), 4.04 (2H, t, *J* = 7.2 Hz, H-16), 5.28 (1H, t, *J* = 7.2 Hz, H-17), 6.31 (1H, s, H-4), 6.66 (1H, s, H-5); ^{13}C-NMR (125 MHz, $CDCl_3$) δ 18.3 (C-20), 20.6 (C-14), 25.6 (C-15), 26.0 (C-19), 27.0 (C-11), 27.1 (C-16), 61.2 (OCH_3-7), 69.6 (C-12), 79.5 (C-13), 94.4 (C-4), 102.3 (C-5), 105.4 (C-2), 107.6 (C-9a), 114.9 (C-8a), 125.7 (C-17), 131.4 (C-18), 138.3 (C-8), 144.8 (C-7), 155.7 (C-10a), 156.2 (C-1), 156.7 (C-6), 158.3 (C-4a), 162.2 (C-3), 178.9 (C-9); ESI-MS *m/z* 449 $[M+Na]^+$; HR-ESI-MS *m/z* 449.15761 $[M+Na]^+$ (calcd for $C_{23}H_{25}O_7$, 449.15762).

α-Mangostin (4)

Yellowish needles (MeOH); mp 182–184 °C; UV (MeOH) λ_{max} (log ε) 243 (4.38), 256 (sh, 4.28), 316 (4.23), 348 (sh, 3.73) nm; IR (neat) ν_{max} 3387 (OH), 1643 (C=O) cm^{-1}; ^{1}H-NMR (600 MHz, $CDCl_3$) δ 1.70 (3H, s, H-20), 1.78 (3H, s, H-19), 1.84 (3H, s, H-15), 1.85 (3H, s, H-14), 3.46 (2H, d, *J* = 7.3 Hz, H-11), 3.81

(3H, s, OMe-7), 4.09 (2H, d, *J* = 6.4 Hz, H-16), 5.27 (1H, br t, *J* = 6.4 Hz, H-17), 5.29 (1H, br t, *J* = 7.3 Hz, H-12), 6.30 (1H, s, H-4), 6.32 (1H, br s, D_2O exchangeable, OH-3), 6.83 (1H, s, H-5), 13.78 (1H, s, D_2O exchangeable, OH-1); ESI-MS *m/z* 409 $[M–H]^-$.

γ-Mangostin (5)

Yellowish needles (MeOH); mp 206–208 °C; UV (MeOH) λ_{max} (log ε) 243 (4.38), 256 (sh, 4.28), 316 (4.23), 348 (sh, 3.73) nm; IR (neat) $_{max}$ 3379 (OH), 1643 (C=O) cm^{-1}; ^{1}H-NMR (500 MHz, acetone-d_6) 1.63 (6H, s, H-15 and 20), 1.77 (3H, s, H-14), 1.83 (3H, s, H-19), 3.34 (2H, d, *J* = 7.3 Hz, H-11), 4.18 (2H, d, *J* = 7.0 Hz, H-16), 5.27 (1H, br t, *J* = 7.3 Hz, H-12), 5.31 (1H, br t, *J* = 7.0 Hz, H-17), 6.36 (1H, s, H-4), 6.79 (1H, s, H-5), 13.95 (1H, s, D_2O exchangeable, OH-1); ESI-MS *m/z* 395 $[M–H]^-$.

Garcinone D (6)

Yellowish needles (MeOH); mp 202–204 °C; UV (MeOH) λ_{max} (log ε) 243 (4.38), 256 (sh, 4.28), 316 (4.23), 348 (sh, 3.73) nm; IR (neat) ν_{max} 3377, 3190 (OH), 1643 (C=O) cm^{-1}; ^{1}H-NMR (500 MHz, acetone-d_6) δ 1.29 (6H, s, H-19 and 20), 1.63 (3H, s, H-15), 1.75 (2H, m, H-17), 1.77 (3H, s, H-14), 3.34 (2H, d, *J* = 7.2 Hz, H-11), 3.45 (2H, m, H-16), 3.85 (3H, s, OMe-7), 5.28 (1H, br t, *J* = 7.2 Hz, H-12), 6.38 (1H, s, H-4), 6.78 (1H, s, H-5), 13.92 (1H, s, D_2O exchangeable, OH-1); ESI-MS *m/z* 427 $[M–H]^-$.

Gartanin (7)

Yellowish needles (MeOH); mp 166–168 °C; UV (MeOH) λ_{max} (log ε) 223 (4.28), 257 (4.24), 283 (4.20), 350 (3.99) nm; IR (neat) ν_{max} 3377 (OH), 1653 (C=O) cm^{-1}; ^{1}H-NMR (400 MHz, $CDCl_3$) δ 1.77 (3H, s, H-20), 1.81 (3H, s, H-15), 1.88 (6H, s, H-14 and 19), 3.48 (2H, d, *J* = 6.8 Hz, H-11), 3.54 (2H, d, *J* = 6.2 Hz, H-16), 5.11 (1H, s, D_2O exchangeable, OH-3), 5.26 (1H, br t, *J* = 6.8 Hz, H-12), 5.29 (1H, br t, *J* = 6.2 Hz, H-17), 6.63 (1H, s, D_2O exchangeable, OH-5), 6.69 (1H, d, *J* = 8.9 Hz, H-7), 7.25 (1H, d, *J* = 8.9 Hz, H-6), 11.29 (1H, s, D_2O exchangeable, OH-8), 12.37 (1H, s, D_2O exchangeable, OH-1); ESI-MS *m/z* 395 $[M–H]^-$.

β-Mangostin (8)

Yellowish needles (MeOH); mp 175–176 °C; UV (MeOH) λ_{max} (log ε) 243 (4.47), 259 (4.42), 315 (4.33) nm; IR (neat) ν_{max} 3391 (OH), 1643 (C=O) cm^{-1}; ^{1}H-NMR (400 MHz, $CDCl_3$) δ 1.68 (6H, s, H-15 and 20), 1.80 (3H, s, H-14), 1.83 (3H, s, H-19), 3.35 (2H, d, *J* = 6.7 Hz, H-11), 3.81 (3H, s, OMe-7), 3.91 (3H, s, OMe-3), 4.10 (2H, d, *J* = 6.4 Hz, H-16), 5.23 (1H, br t, *J* = 6.7 Hz, H-12), 5.26 (1H, br t, *J* = 6.4 Hz, H-17), 6.34 (1H, s, H-4), 6.83 (1H, s, H-5), 13.42 (1H, s, D_2O exchangeable, OH-1); ESI-MS *m/z* 423 $[M–H]^-$.

Dulcisxanthone D (9)

Yellowish needles (MeOH); mp 218–220 °C; UV (MeOH) λ_{max} (log ε) 256 (4.30), 289 (4.51), 300 (4.45), 335 (4.28), 386 (sh, 3.70) nm; IR (neat) ν_{max} 3518, 3393 (OH), 1651 (C=O) cm^{-1}; ^{1}H-NMR (400 MHz, $CDCl_3$) δ 1.48 (6H, s, H-14 and 15), 1.49 (6H, s, H-24 and 25), 1.69 (3H, s, H-20), 1.88 (3H, s, H-19), 3.57 (2H, d, *J* = 7.6 Hz, H-16), 5.27 (1H, br t, *J* = 7.6 Hz, H-17), 5.58 (1H, d, *J* = 10.0 Hz, H-12), 5.78 (1H, d, *J* = 10.1 Hz, H-22), 6.31 (1H, s, H-4), 6.31 (1H, s, D_2O exchangeable, OH-6), 6.73 (1H, d, *J* = 10.0 Hz, H-11), 7.99 (1H, d, *J* = 10.1 Hz, H-21), 13.70 (1H, s, D_2O exchangeable, OH-1); ESI-MS *m/z* 459 $[M–H]^-$.

Brasilixanthone B (10)

Yellowish needles (acetone); mp 229–231 °C; UV (MeOH) λ_{max} (log ε) 256 (4.30), 288 (4.54), 300 (4.48), 335 (4.28), 385 (sh, 3.72) nm; IR (neat) ν_{max} 3507, 3395 (OH), 1649 (C=O) cm^{-1}; ^{1}H-NMR (400 MHz, $CDCl_3$) δ 1.47 (6H, s, H-14 and 15), 1.50 (6H, s, H-19 and 20), 5.58 (1H, d, *J* = 9.7 Hz, H-12), 5.83 (1H, d, *J* = 10.1 Hz, H-17), 6.23 (1H, s, D_2O exchangeable, OH-6), 6.26 (1H, s, H-4), 6.73 (1H, d, *J* = 10.1 Hz, H-11), 6.83 (1H, s, H-5), 8.02 (1H, d, *J* = 10.1 Hz, H-16), 13.63 (1H, s, D_2O exchangeable, OH-1); ESI-MS *m/z* 393 $[M+H]^+$.

8-Hydroxycudraxanthone G (11)

Yellow amorphous powder; UV (MeOH) λ_{max} (log ε) 237 (4.25), 262 (4.31), 278 (4.20), 350 (3.98) nm; IR (neat) ν_{max} 3397 (OH), 1651 (C=O) cm^{-1}; ^{1}H-NMR (600 MHz, $CDCl_3$) δ 1.71 (3H, s, H-20), 1.74 (3H, s, H-15), 1.81 (3H, s, H-19), 1.87 (3H, s, H-14), 3.42 (2H, d, *J* = 6.8 Hz, H-11), 3.55 (2H, d, *J* = 6.5 Hz, H-16), 3.82 (3H, s, OMe-3), 5.13 (1H, s, D_2O exchangeable, OH-5), 5.24 (2H, br t, *J* = 6.8 Hz, H-12), 5.24 (2H, br t, *J* = 6.5 Hz, H-17), 6.70 (1H, d, *J* = 8.8 Hz, H-7), 7.26 (1H, d, *J* = 8.8 Hz, H-6), 11.14 (1H, s, D_2O exchangeable, OH-8), 12.13 (1H, s, D_2O exchangeable, OH-1); ESI-MS *m/z* 409 $[M–H]^-$.

Tovophyllin A (12)

Yellow needles (CH_2Cl_2-MeOH); mp 218–220 °C; UV (MeOH) λ_{max} (log ε) 245 (4.39), 262 (4.41), 324 (4.17), 363 (sh, 3.90) nm; IR (neat) ν_{max} 3385 (OH), 1643 (C=O) cm^{-1}; ^{1}H-NMR (500 MHz, $CDCl_3$) δ1.69 (3H, s, H-15), 1.78 (3H, s, H-19), 1.85 (3H, s, H-25), 1.88 (3H, s, H-19), 3.47 (2H, d, *J* = 7.2 Hz, H-11), 3.59 (2H, d, *J* = 7.4 Hz, H-16), 5.28 (1H, br t, *J* = 7.2 Hz, H-12), 5.30 (1H, br t, *J* = 7.4 Hz, H-17), 5.77 (1H, d, *J* = 10.2 Hz, H-22), 6.35 (1H, s, H-4), 6.14 (1H, br s, D_2O exchangeable, OH-3), 6.30 (1H, br s, D_2O exchangeable, OH-6), 8.01 (1H, d, *J* = 10.2 Hz, H-21), 13.76 (1H, s, D_2O exchangeable, OH-1); ESI-MS *m/z* 461 $[M–H]^-$.

Garcinone E (13)

Yellow needles (EtOAc); mp 153–155 °C; UV (MeOH) λ_{max} (log ε) 246 (4.41), 266 (4.41), 337 (4.32), 380 (3.70) nm; IR (neat) ν_{max} 3518, 3298 (OH), 1649 (C=O) cm^{-1}; ^{1}H-NMR (600 MHz, $CDCl_3$) δ1.71 (3H, s, H-15), 1.78 (6H, s, H-20 and 25), 1.85 (3H, s, H-14), 1.88 (6H, s, H-19 and H-24), 3.47 (2H, d, *J* = 6.5 Hz, H-11), 3.58 (2H, d, *J* = 6.5 Hz, H-16), 4.29 (2H, d, *J* = 6.8 Hz, H-21), 5.28 (1H, br t, *J* = 6.5 Hz, H-12), 5.28 (1H, br t, *J* = 6.5 Hz, H-17), 5.28 (1H, br t, *J* = 6.8 Hz, H-22), 6.34 (1H, s, H-4), 13.83 (1H, s, D_2O exchangeable, OH-1); ESI-MS *m/z* 463 $[M–H]^-$.

Ananixanthone (14)

Yellow needles (MeOH); mp 170–171 °C; UV (MeOH) λ_{max} (log ε) 253 (4.43), 271 (4.41), 332 (4.00), 379 (3.41) nm; IR (neat) ν_{max} 3370 (OH), 1645 (C=O) cm^{-1}; ^{1}H-NMR (400 MHz, $CDCl_3$) δ 1.50 (6H, s, H-19 and H-20), 1.69 (3H, s, H-15), 1.82 (3H, s, H-14), 3.36 (2H, d, *J* = 7.3 Hz, H-11), 5.25 (1H, br t, *J* = 7.3 Hz, H-12), 5.65 (1H, d, *J* = 10.0 Hz, H-17), 6.79 (1H, d, *J* = 10.0 Hz, H-16), 7.24 (1H, dd, *J* = 7.9, 7.9 Hz, H-7), 7.31 (1H, dd, *J* = 7.9, 1.6 Hz, H-6), 7.78 (1H, dd, *J* = 7.9, 1.6 Hz, H-8), 13.20 (1H, s, D_2O exchangeable, OH-1); ESI-MS *m/z* 379 $[M+H]^+$.

Morusignin J (15)

Yellow needles (acetone); mp 184–186 °C; UV (MeOH) λ_{max} (log ε) 228 (4.23), 259 (4.40), 269 (4.38), 364 (3.98) nm; IR (neat) ν_{max} 3408 (OH), 1641 (C=O) cm^{-1}; ^{1}H-NMR (500 MHz, $CDCl_3$) δ 1.50 (6H, s, H-19 and H-20), 1.69 (3H, s, H-15), 1.82 (3H, s, H-14), 3.35 (2H, d, *J* = 7.4 Hz, H-11), 4.99 (1H, br s, D_2O exchangeable, OH-5), 5.23 (1H, br t, *J* = 7.4 Hz, H-12), 5.65 (1H, d, *J* = 10.0 Hz, H-17), 6.68 (1H, d, *J* = 8.7 Hz, H-7), 6.75 (1H, d, *J* = 10.0 Hz, H-16), 7.24 (1H, d, *J* = 8.7 Hz, H-6), 11.32 (1H, s, D_2O exchangeable, OH-8), 12.35 (1H, s, D_2O exchangeable, OH-1); ESI-MS *m/z* 395 $[M+H]^+$.

Fuscaxanthone C (16)

Yellowish needles (MeOH); mp 123–124 °C; UV (MeOH) λ_{max} (log ε) 245 (4.38), 261 (4.47), 313 (4.20) nm; IR (neat) ν_{max} 3400 (OH), 1645 (C=O) cm^{-1}; ^{1}H-NMR (600 MHz, $CDCl_3$) δ 1.68 (6H, s, H-15 and H-20), 1.80 (3H, s, H-14), 1.85 (3H, s, H-19), 3.36 (2H, d, *J* = 7.2 Hz, H-11), 3.80 (3H, s, OMe-7), 3.91 (3H, s, OMe-3), 3.96 (3H, s, OMe-6), 4.14 (2H, d, *J* = 6.7 Hz, H-16), 5.24 (1H, br t, *J* = 7.2 Hz, H-12), 5.25 (1H, br t, *J* = 6.7 Hz, H-17), 6.33 (1H, s, H-4), 6.75 (1H, s, H-5), 13.48 (1H, s, D_2O exchangeable, OH-1); ESI-MS *m/z* 439 $[M+H]^+$.

Pruniflorone R (17)

Yellow amorphous powder; UV (MeOH) λ_{max} (log ε) 244 (4.38), 261 (4.43), 317 (4.22), 360 (3.80) nm; IR (neat) ν_{max} 3485, 3368 (OH), 1645 (C=O) cm^{-1}; ^{1}H-NMR (600 MHz, $CDCl_3$) δ 1.39 (6H, s, H-19 and H-20), 1.68 (3H, s, H-15), 1.80 (3H, s, H-14), 1.89 (2H, t, *J* = 6.8 Hz, H-17), 3.36 (2H, d, *J* = 7.1 Hz, H-11), 3.51 (2H, t, *J* = 6.8 Hz, H-16), 5.24 (1H, br t, *J* = 7.1 Hz, H-12), 6.36 (1H, s, H-4), 6.40 (1H, br s, D_2O exchangeable, OH-7), 6.81 (1H, s, H-5), 13.45 (1H, s, D_2O exchangeable, OH-1); ESI-MS *m/z* 427 $[M-H]^-$.

Mixture of β-Sitosterol (18) and Stigmasterol (19)

Colorless needles; mp 134–136 °C; $[\alpha]_D^{25}$ = −36.7° (*c* 0.8, $CHCl_3$); IR (neat) ν_{max} 3319 (OH) cm^{-1}; ^{1}H-NMR ($CDCl_3$, 400 MHz) of **18**: δ 0.68 (3H, s, H-18), 0.81 (3H, d, *J* = 6.8 Hz, H-27), 0.83 (3H, d, *J* = 6.8 Hz, H-26), 0.85 (3H, t, *J* = 7.5 Hz, H-29), 0.92 (3H, d, *J* = 6.5 Hz, H-21), 1.01 (3H, s, H-19), 3.52 (1H, m, OH-3), 5.35 (1H, br d, *J* = 5.2 Hz, H-6); ^{1}H-NMR ($CDCl_3$, 400 MHz) of **19**: δ 0.70 (3H, s, H-18), 0.79 (3H, d, *J* = 6.8 Hz, H-27), 0.82 (3H, t, *J* = 7.2 Hz, H-29), 0.83 (3H, d, *J* = 6.8 Hz, H-26), 1.01 (3H, s, H-19) , 1.02 (3H, d, *J* = 6.5 Hz, H-21), 3.52 (1H, m, OH-3), 5.01 (1H, dd, *J* = 15.2, 8.8 Hz, H-23), 5.15 (1H, dd, *J* = 15.2, 8.4 Hz, H-22), 5.35 (1H, br d, *J* = 5.2 Hz, H-6).

Mixture of β-Sitostenone (20) and Stigmasta-4,22-dien-3-one (21)

Colorless needles (MeOH); mp 87–89 °C; $[\alpha]_D^{26}$ = +85.4° (*c* 0.21, $CHCl_3$); UV (MeOH) λ_{max} (log ε) 243 (4.22) nm; IR (neat) ν_{max} 1676 (C=O) cm^{-1}; ^{1}H-NMR ($CDCl_3$, 400 MHz) of **20**: δ 0.71 (3H, s, H-18), 0.81 (3H, d, *J* = 6.8 Hz, H-27), 0.83 (3H, d, *J* = 6.8 Hz, H-26), 0.86 (3H, t, *J* = 7.2 Hz, H-29), 0.92 (3H, d, *J* = 6.4 Hz, H-21), 1.18 (3H, s, H-19), 5.72 (1H, s, H-4); ^{1}H-NMR ($CDCl_3$, 400 MHz) of **21**: δ 0.73 (3H, s, H-18), 0.79 (3H, d, *J* = 6.8 Hz, H-27), 0.82 (3H, d, *J* = 7.2 Hz, H-29), 0.83 (3H, t, *J* = 6.8 Hz, H-26), 1.02 (3H, d, *J* = 6.8 Hz, H-21), 1.18 (3H, s, H-19), 5.02 (1H, dd, *J* = 15.2, 8.7 Hz), 5.14 (1H, dd, *J* = 15.2, 8.6 Hz), 5.72 (1H, s, H-4).

MS, IR, and NMR spectrum of isolated compounds

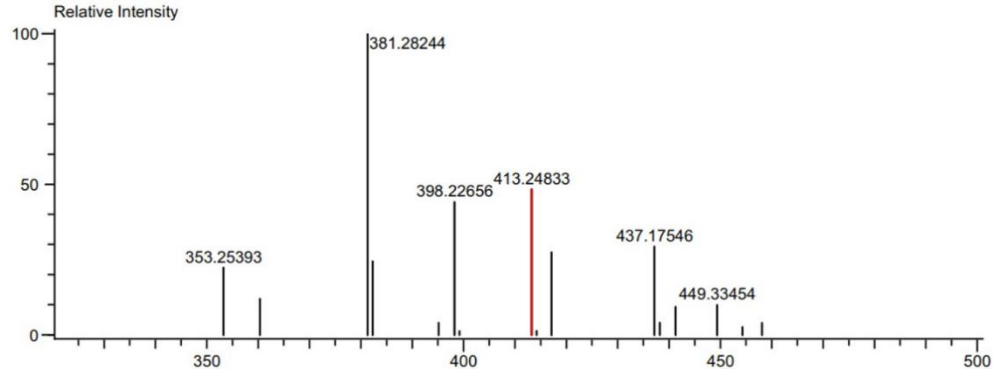

Figure S1. ESI-MS spectrum of compound **1**.

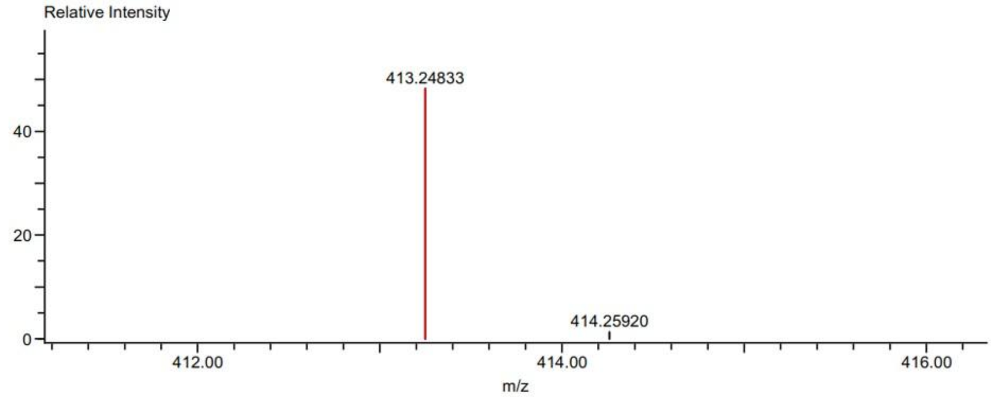

Mass	Intensity	Calc. Mass	Mass Difference [mDa]	Mass Difference [ppm]	Possible Formula
413.24833	1851.82	413.16003	88.30	213.73	$^{12}C_{23}{}^{1}H_{25}{}^{16}O_{7}$

Figure S2. HR-ESI-MS spectrum of compound **1**.

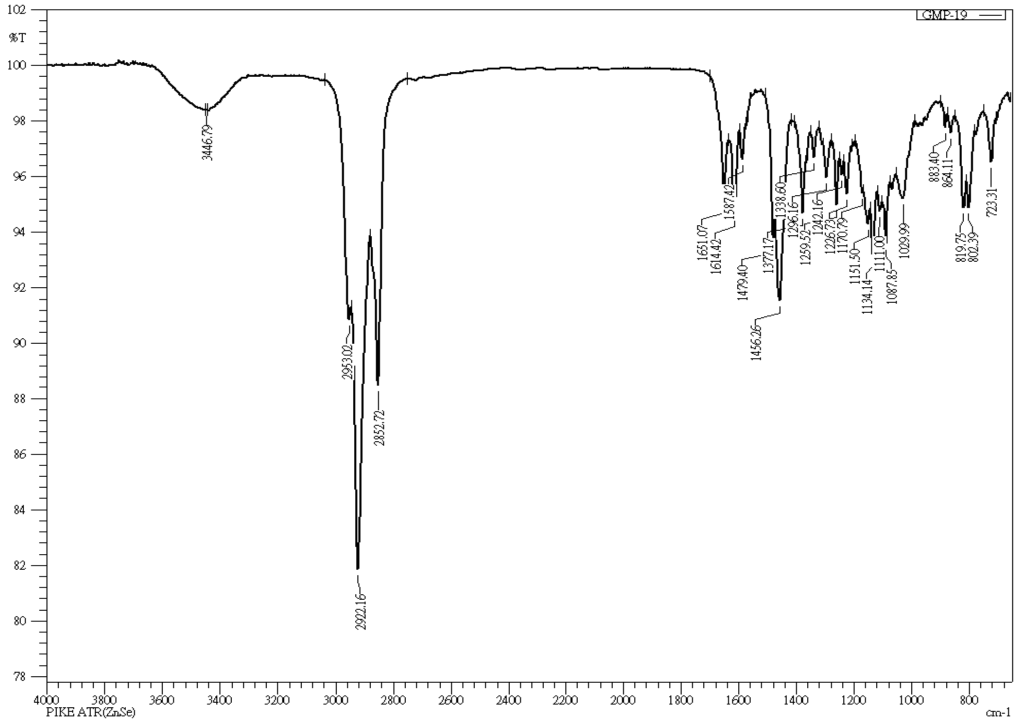

Figure S3. IR spectrum of compound **1**.

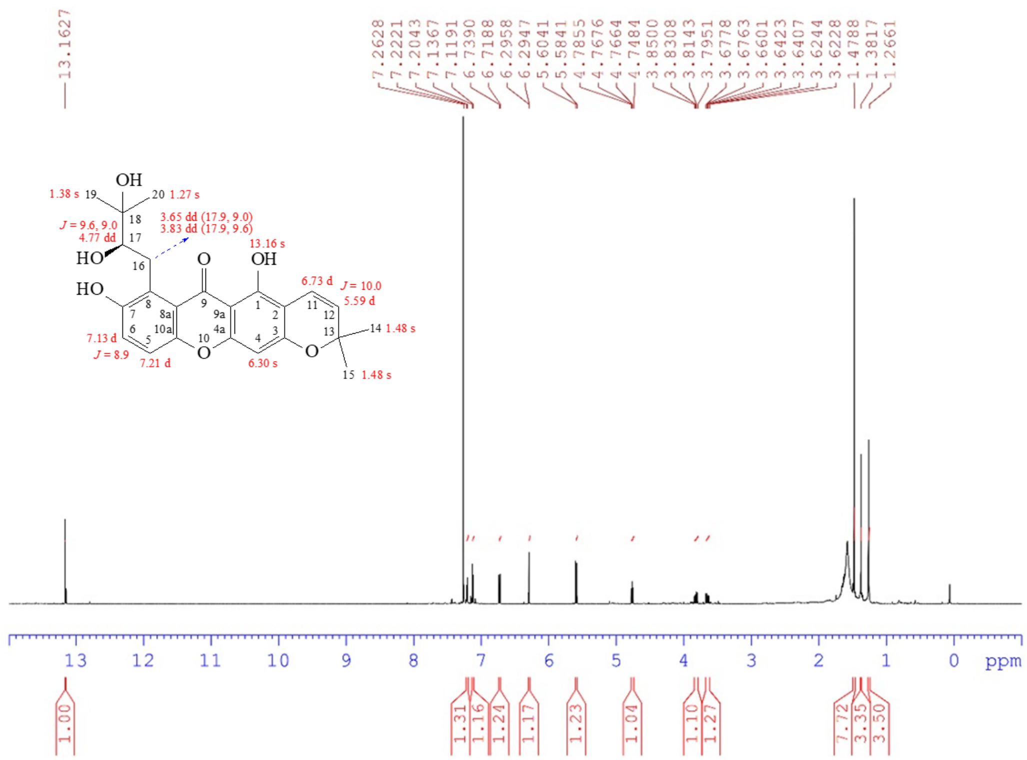

Figure S4. ^{1}H-NMR spectrum of compound **1**.

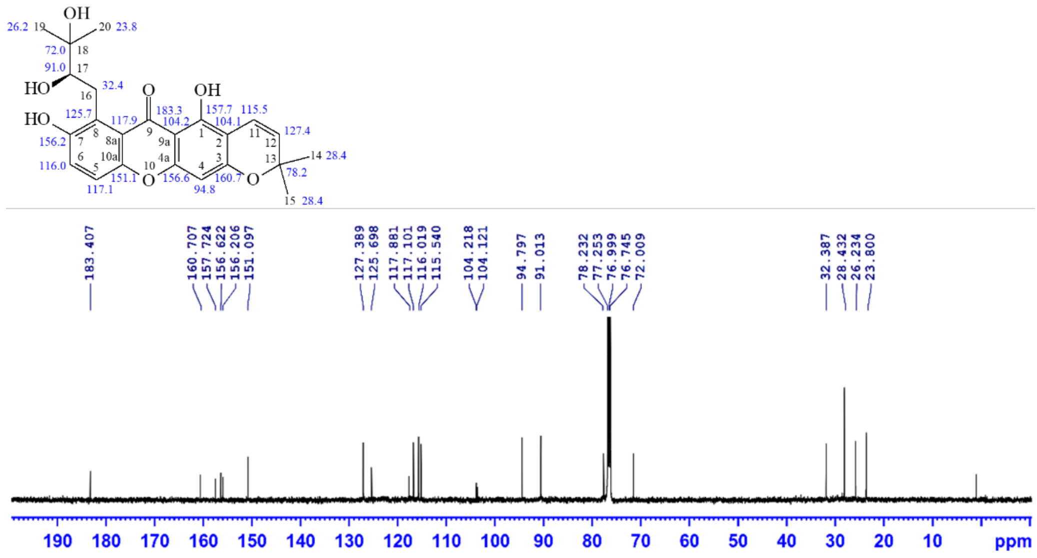

Figure S5. ^{13}C-NMR spectrum of compound **1**.

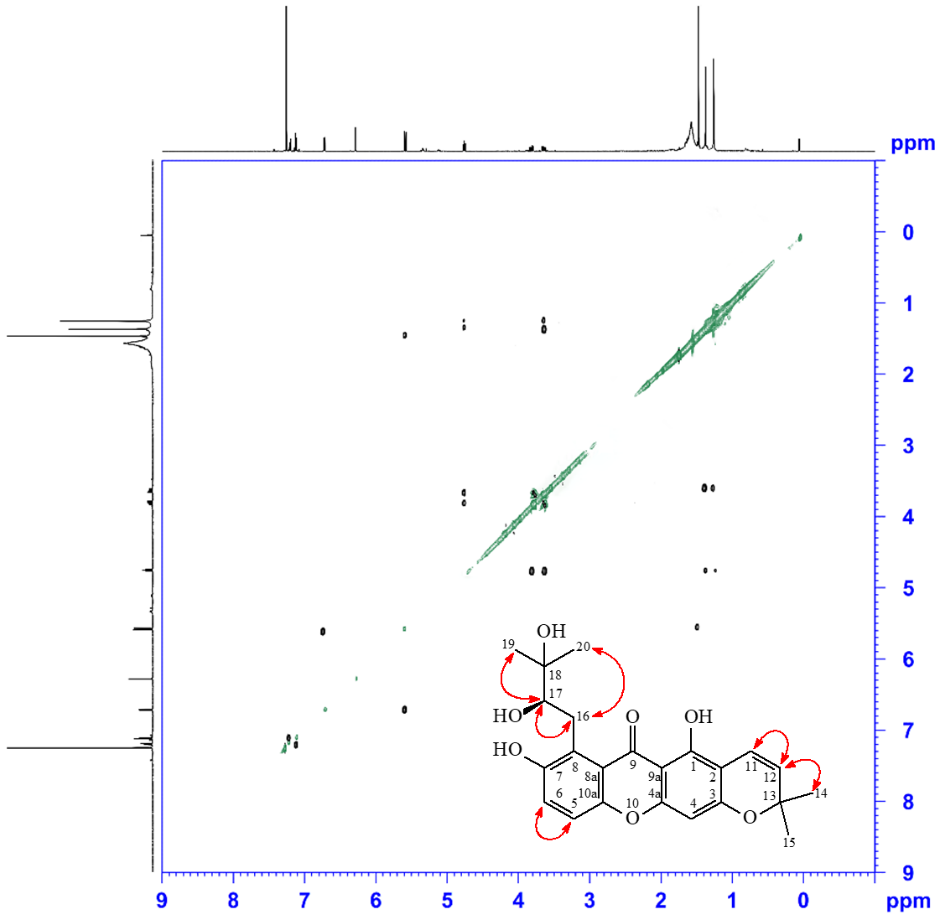

Figure S6. ROESY spectrum of compound **1**.

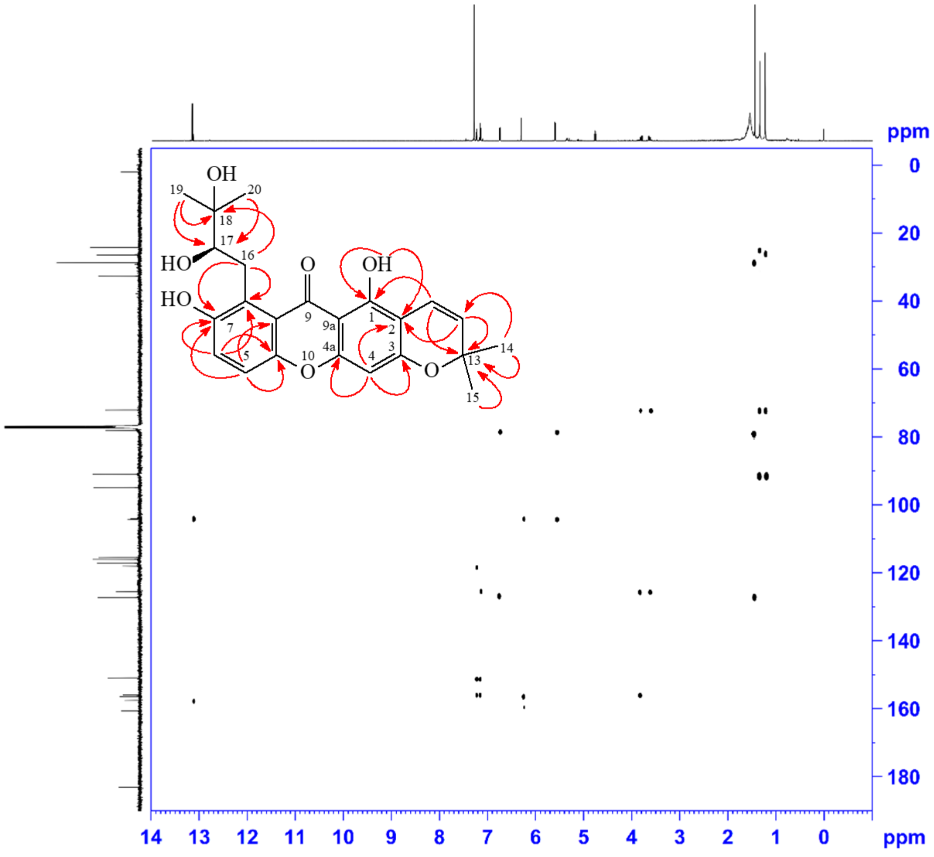

Figure S7. HMBC spectrum of compound **1**.

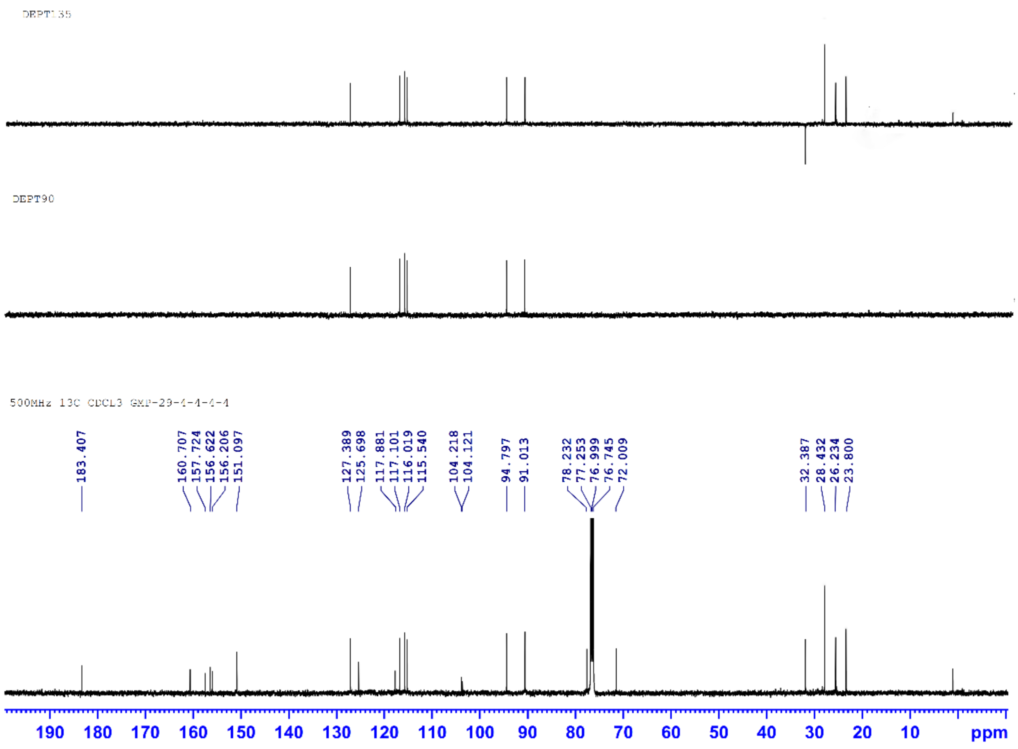

Figure S8. DEPT spectrum of compound **1**.

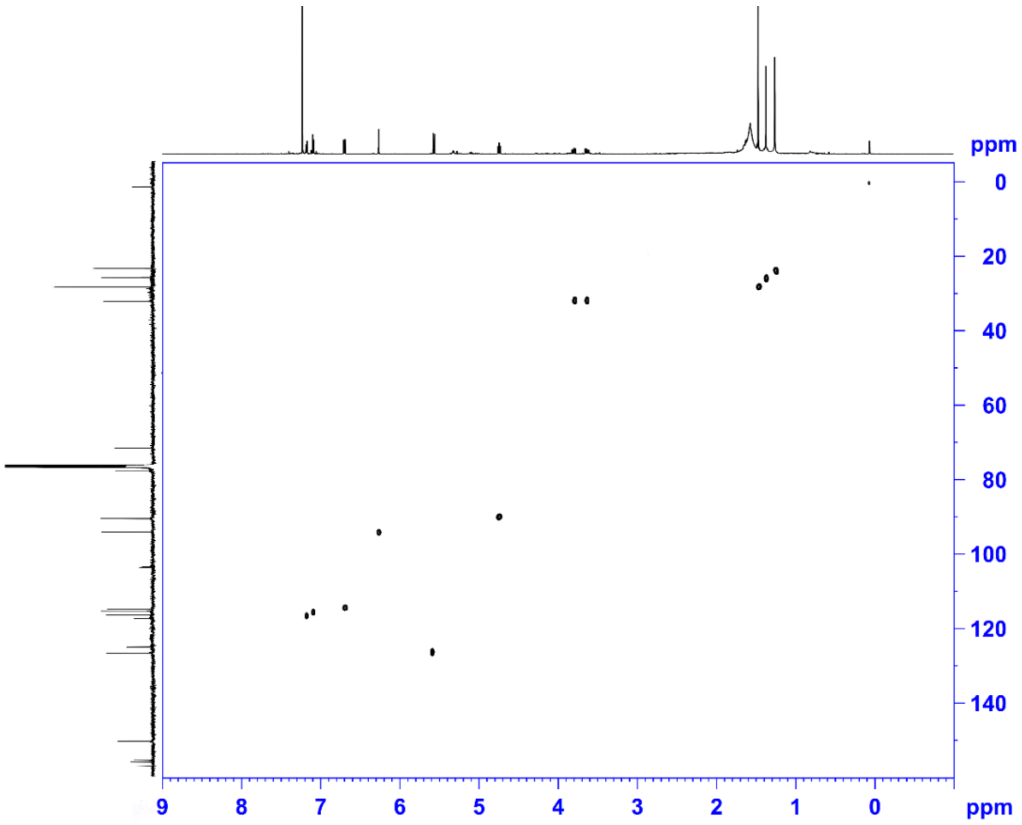

Figure S9. HSQC spectrum of compound **1**.

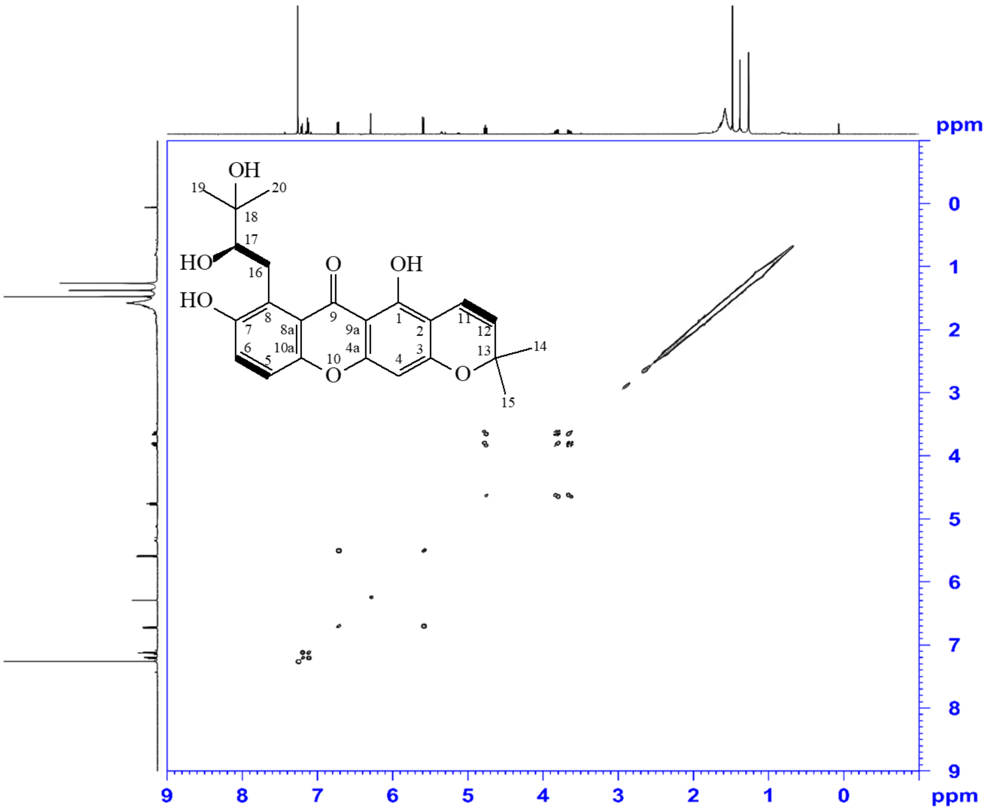

Figure S10. ^{1}H-^{1}H COSY spectrum of compound **1**.

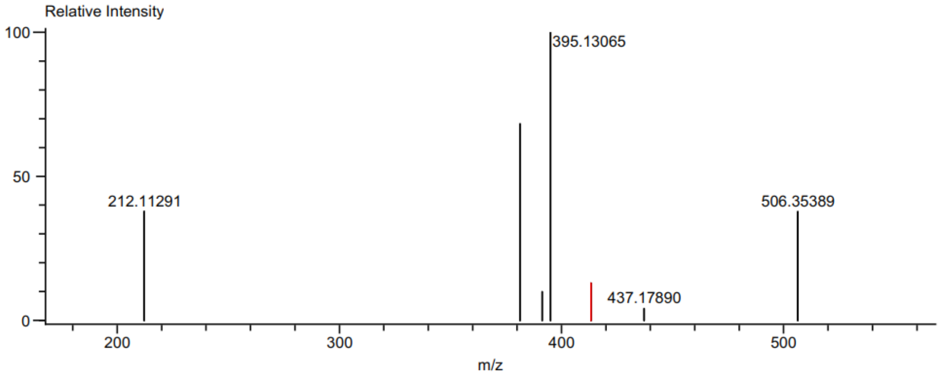

Figure S11. ESI-MS spectrum of compound **2**.

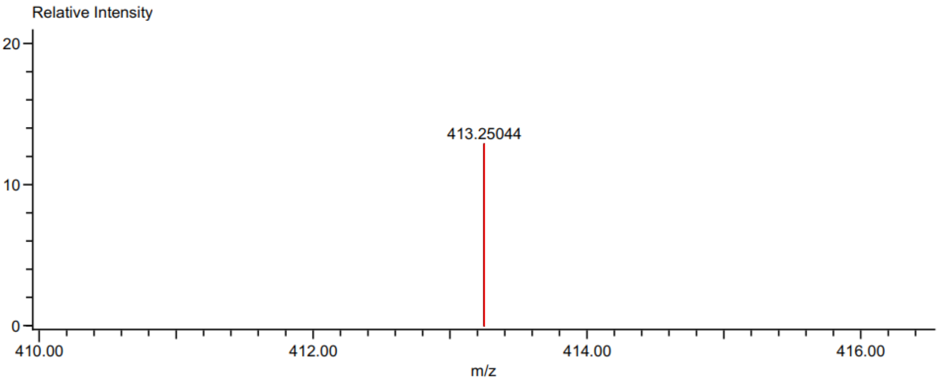

Mass	Intensity	Calc. Mass	Mass Difference [mDa]	Mass Difference [ppm]	Possible Formula
413.25044	191.28	413.16003	90.41	218.83	$^{12}C_{23}{}^{1}H_{25}{}^{16}O_{7}$

Figure S12. HR-ESI-MS spectrum of compound **2**.

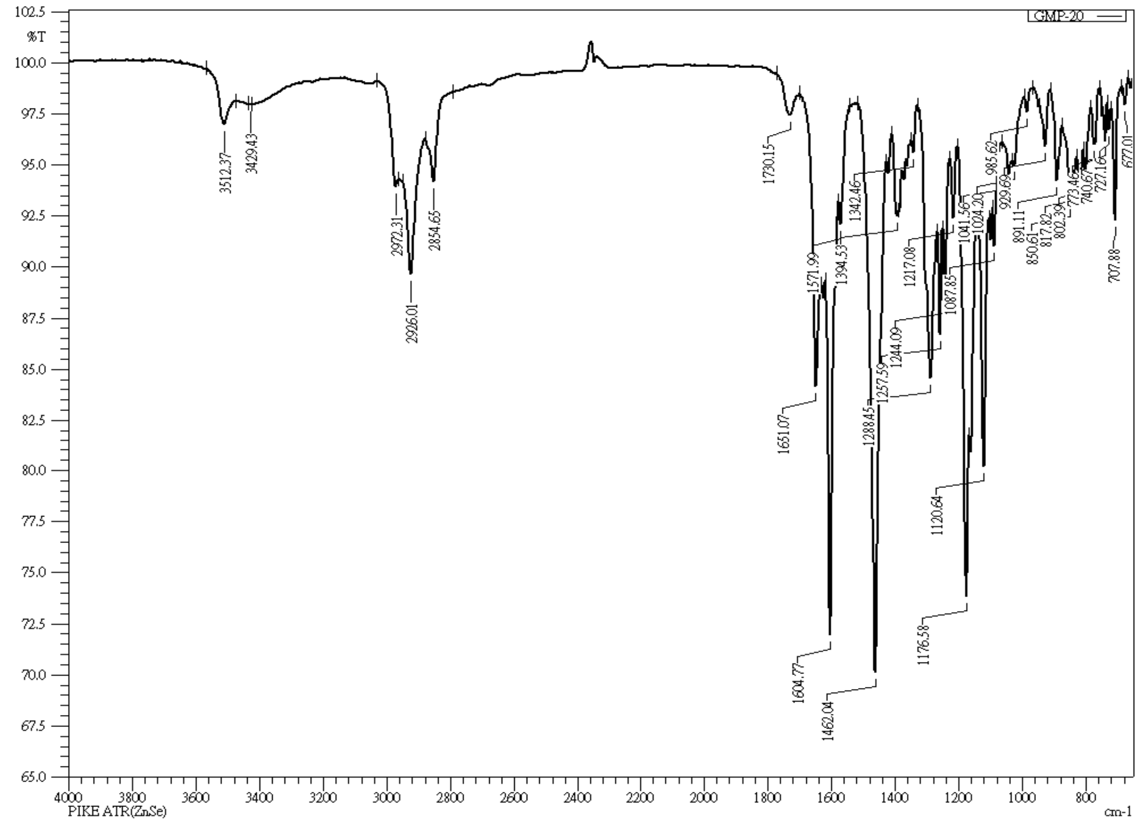

Figure S13. IR spectrum of compound **2**.

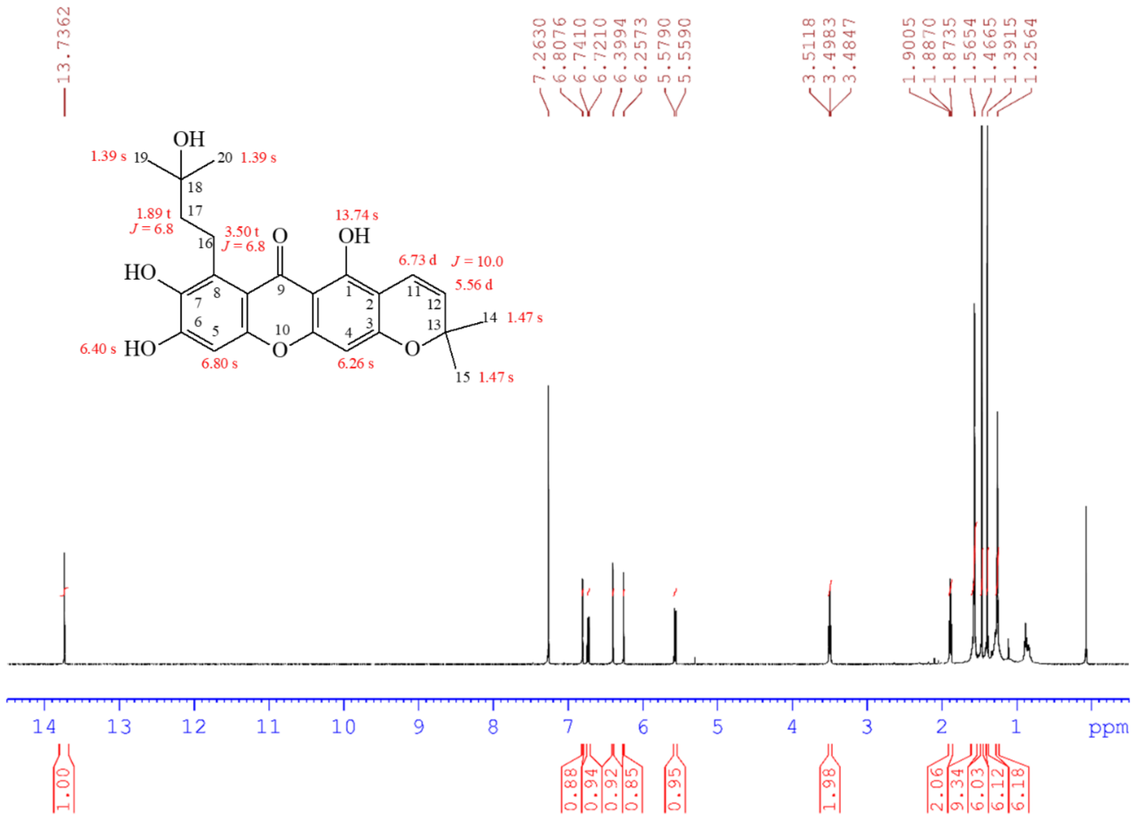

Figure S14. ^{1}H-NMR spectrum of compound **2**.

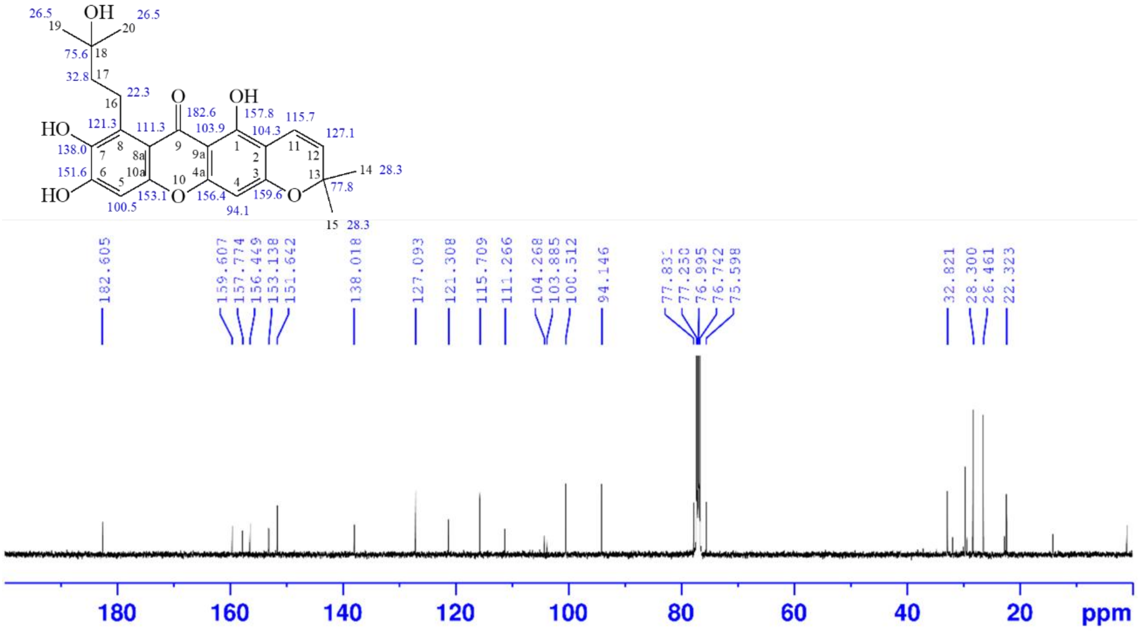

Figure S15. ^{13}C-NMR spectrum of compound **2**.

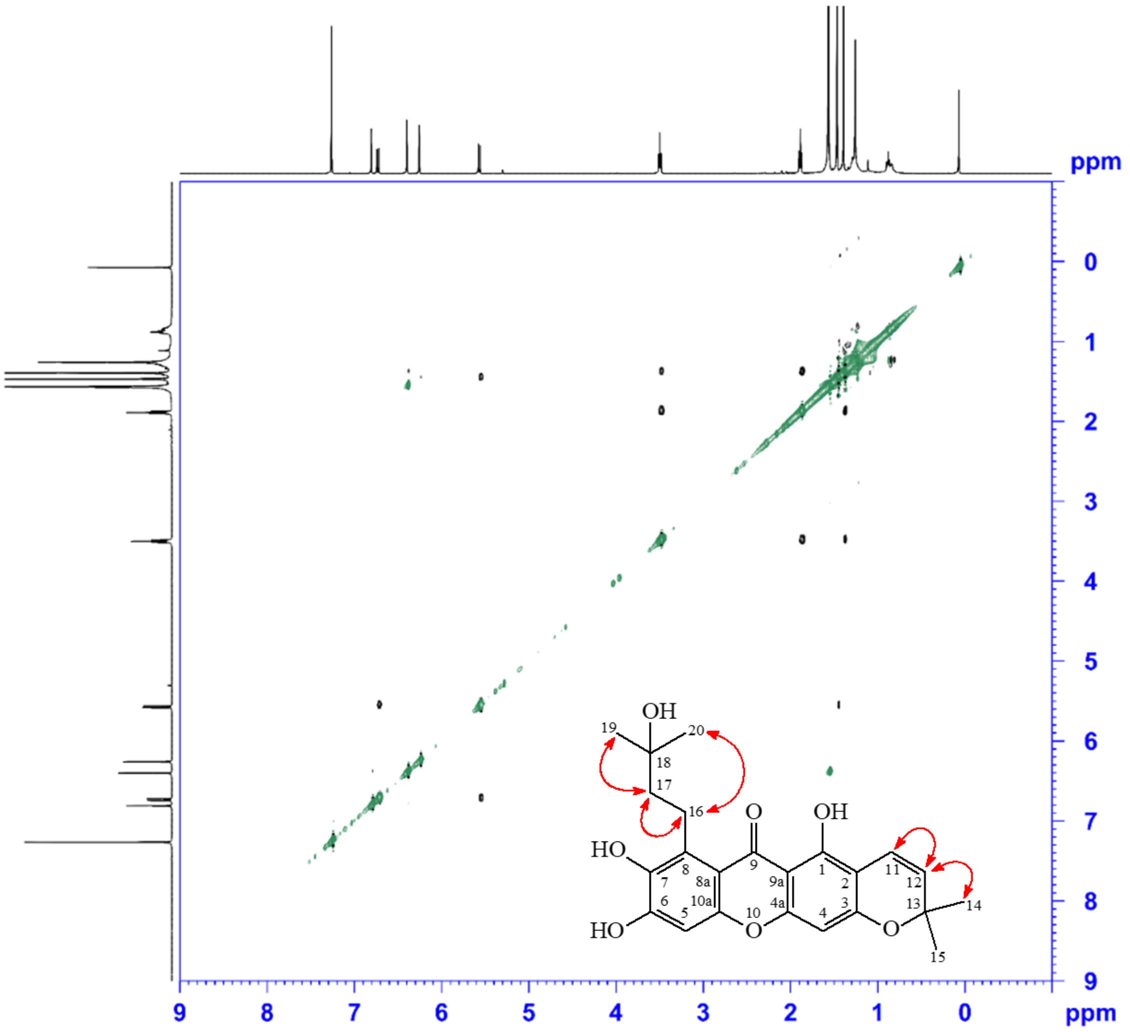

Figure S16. ROESY spectrum of compound **2**.

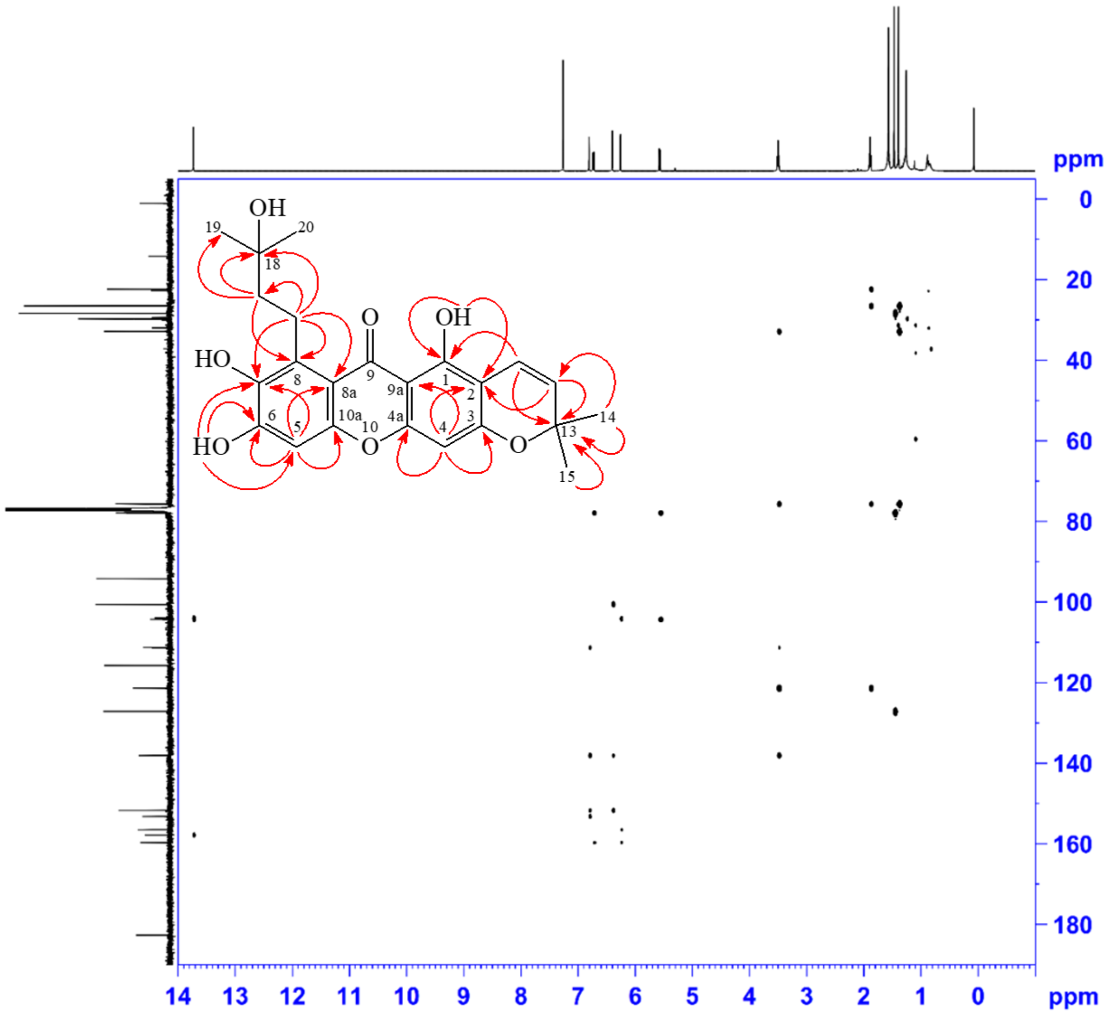

Figure S17. HMBC spectrum of compound **2**.

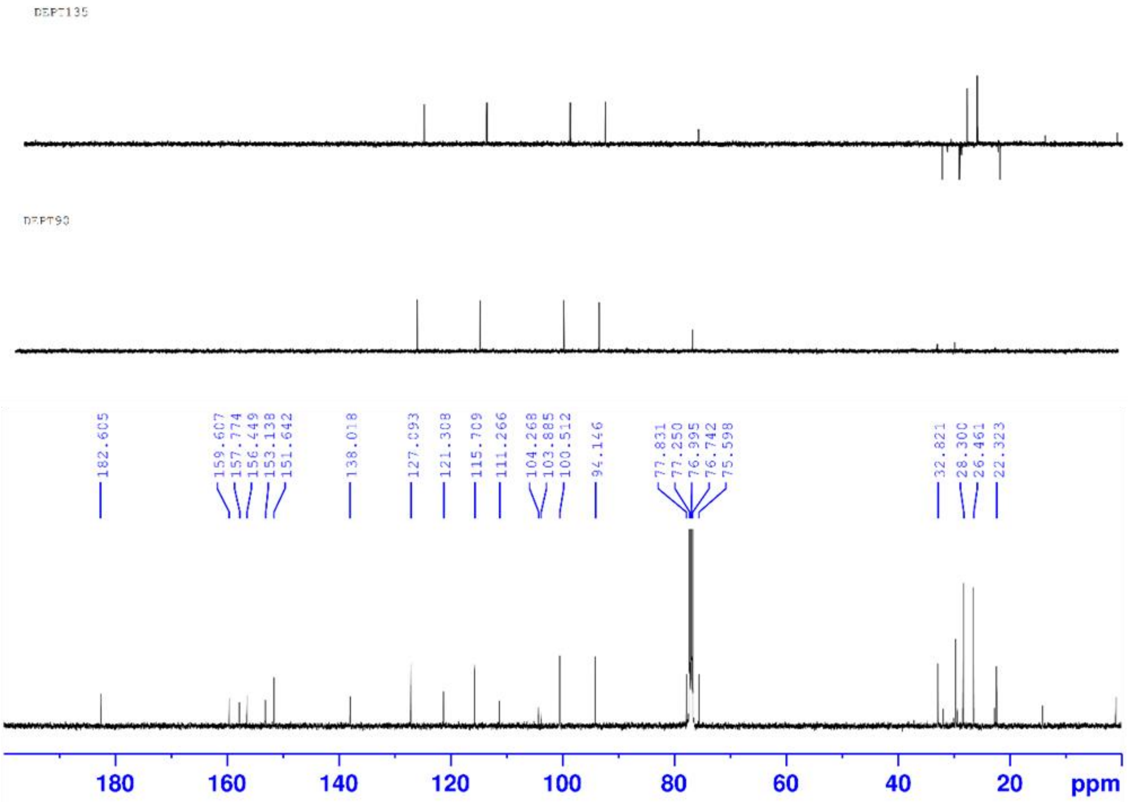

Figure S18. DEPT spectrum of compound **2**.

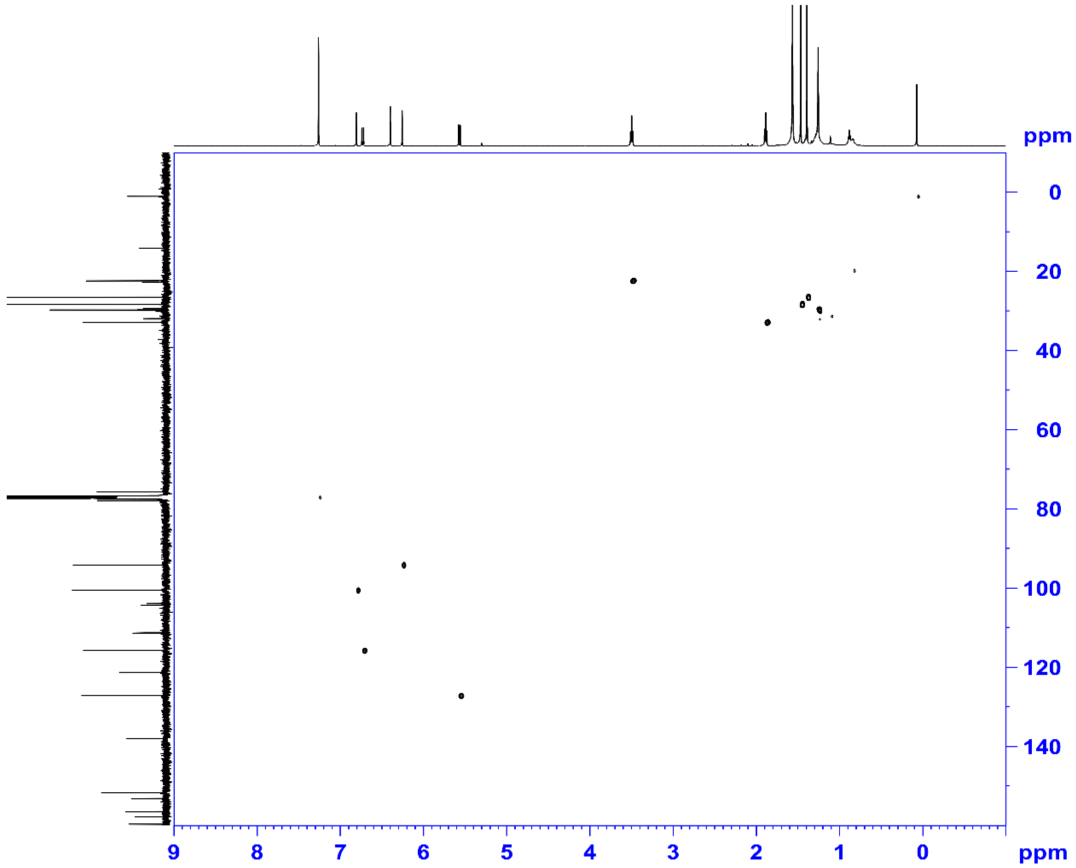

Figure S19. HSQC spectrum of compound **2**.

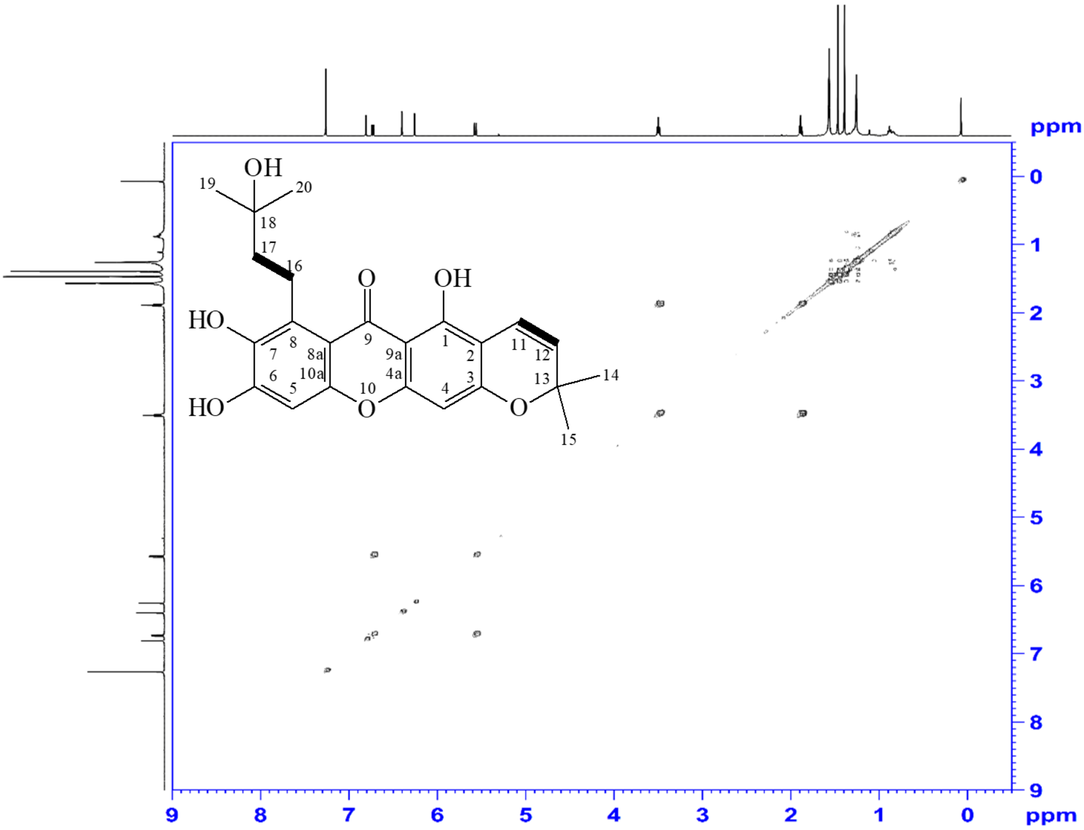

Figure S20. ^{1}H-^{1}H COSY spectrum of compound **2**.

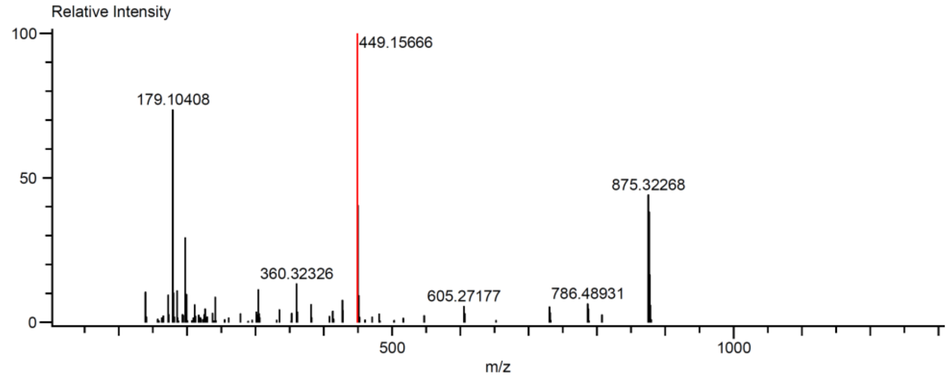

Figure S21. ESI-MS spectrum of compound **3**.

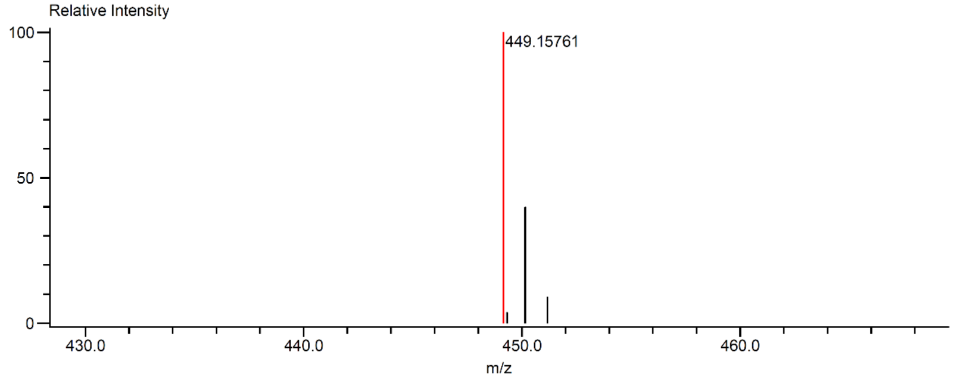

Mass	Intensity	Calc. Mass	Mass Difference [mDa]	Mass Difference [ppm]	Possible Formula
449.15761	18211.82	449.15762	-0.01	-0.03	$^{12}C_{24}{}^{1}H_{26}{}^{23}Na_{1}{}^{16}O_{7}$

Figure S22. HR-ESI-MS spectrum of compound **3**.

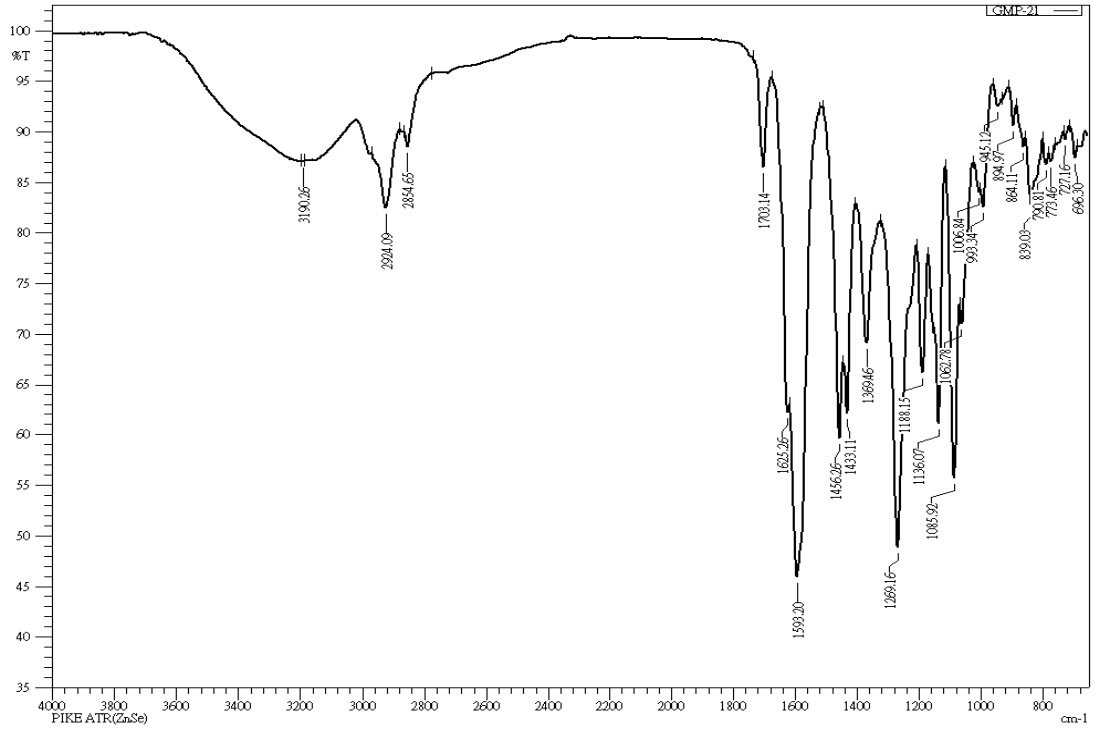

Figure S23. IR spectrum of compound **3**.

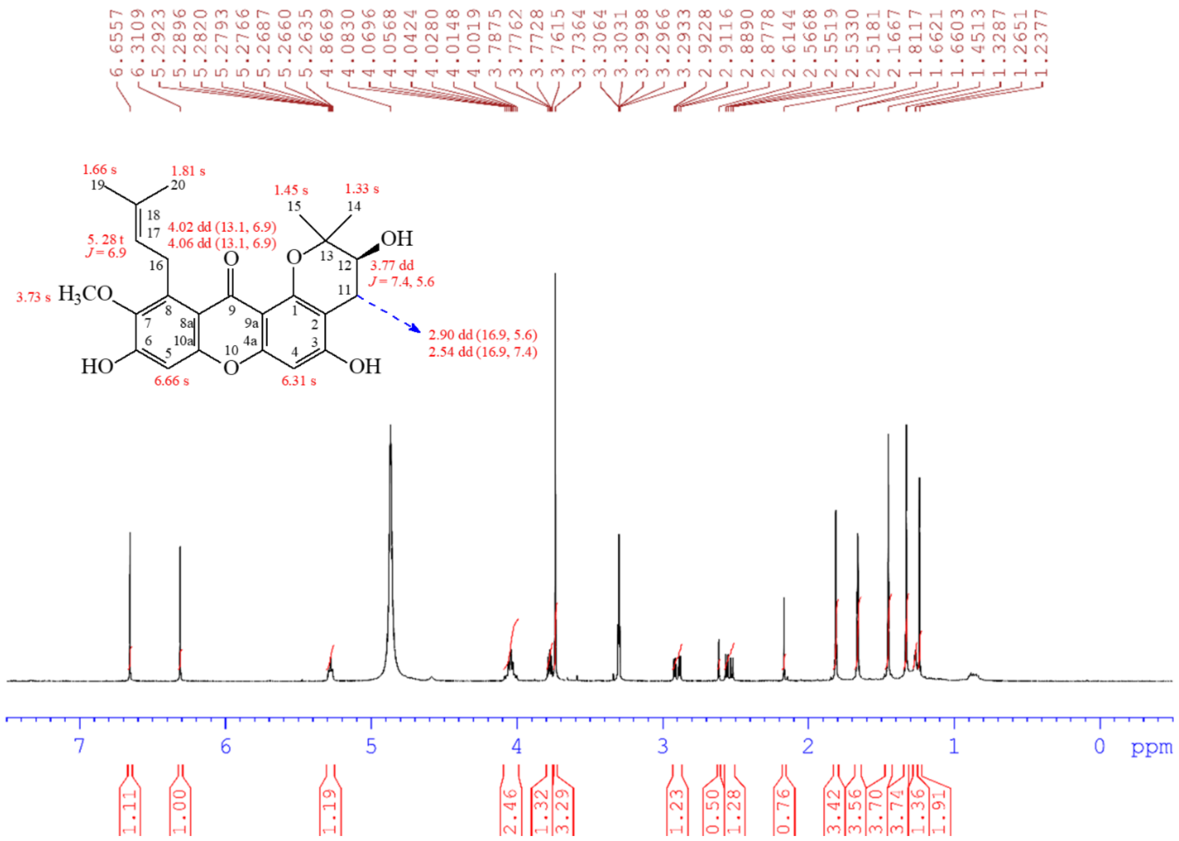

Figure S24. ^{1}H-NMR spectrum of compound **3**.

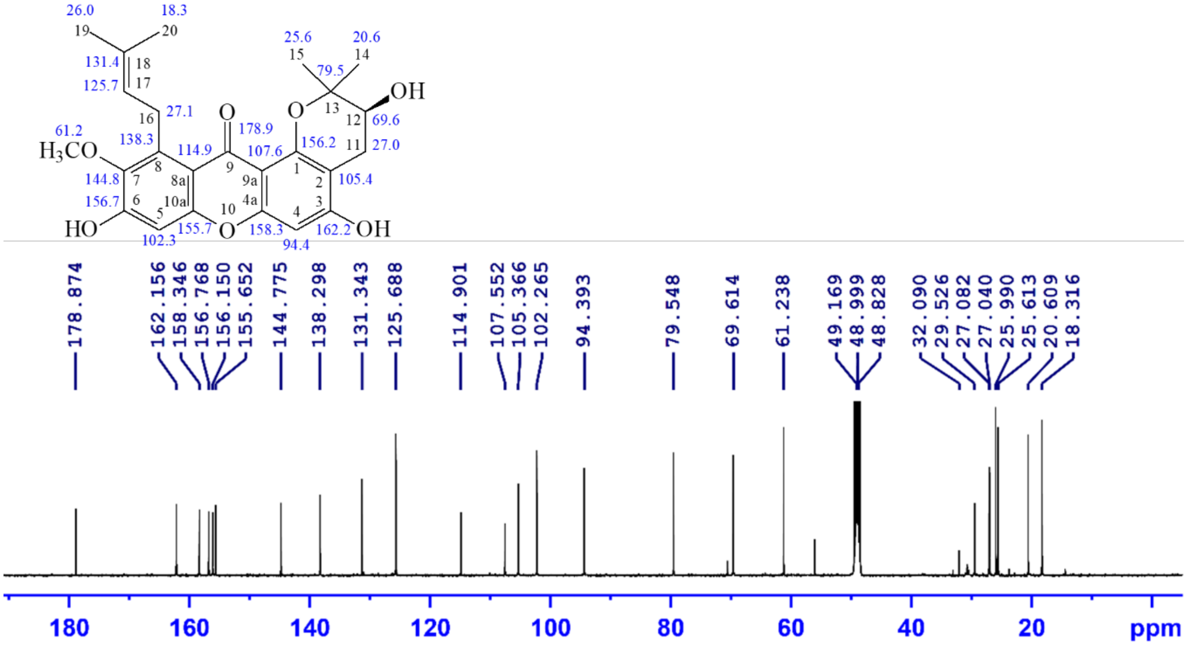

Figure S25. ^{13}C-NMR spectrum of compound **3**.

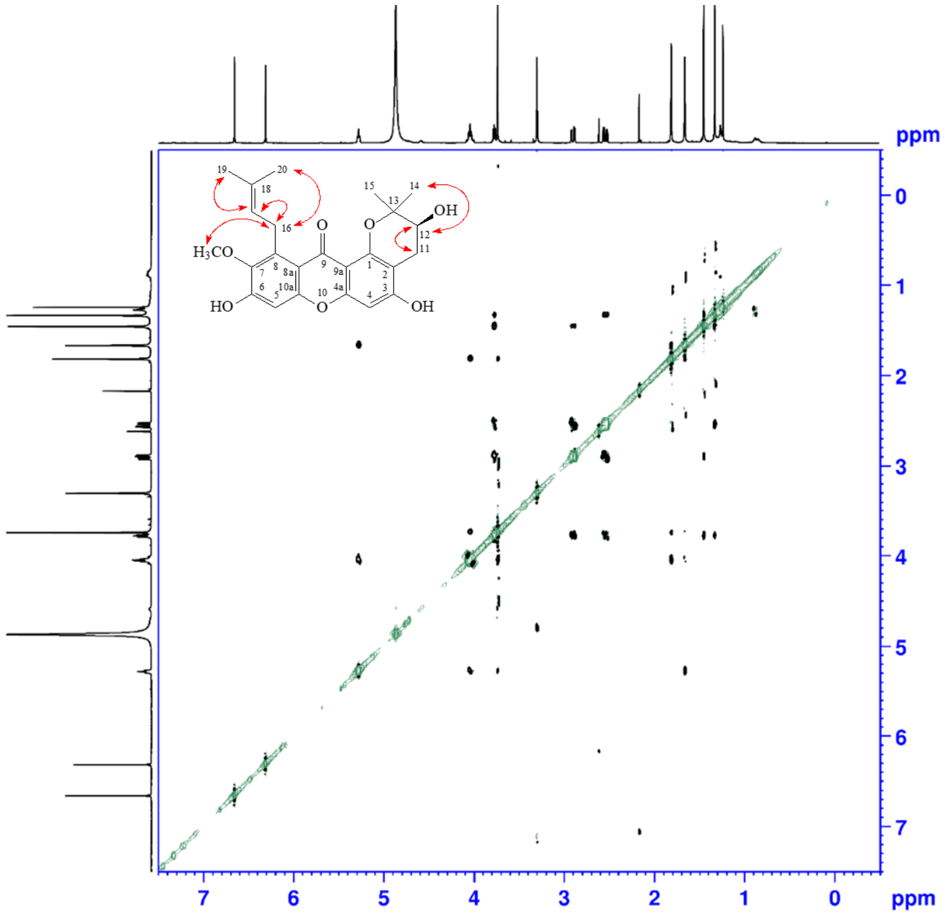

Figure S26. ROESY spectrum of compound **3**.

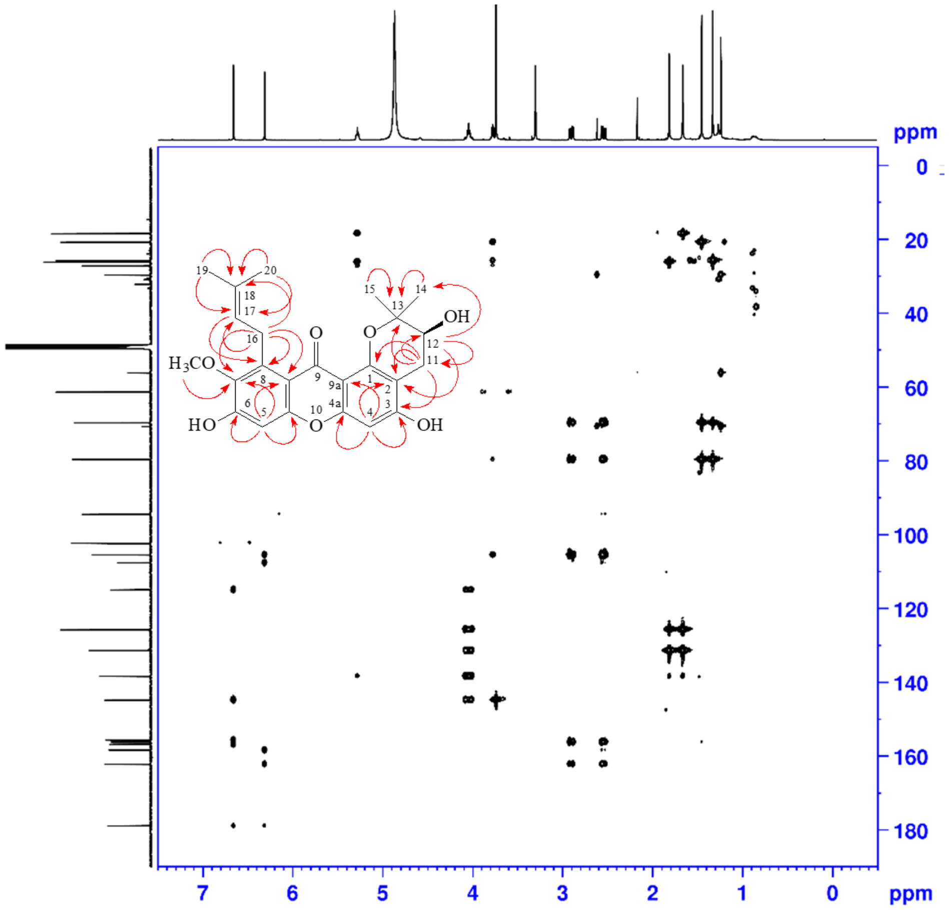

Figure S27. HMBC spectrum of compound **3**.

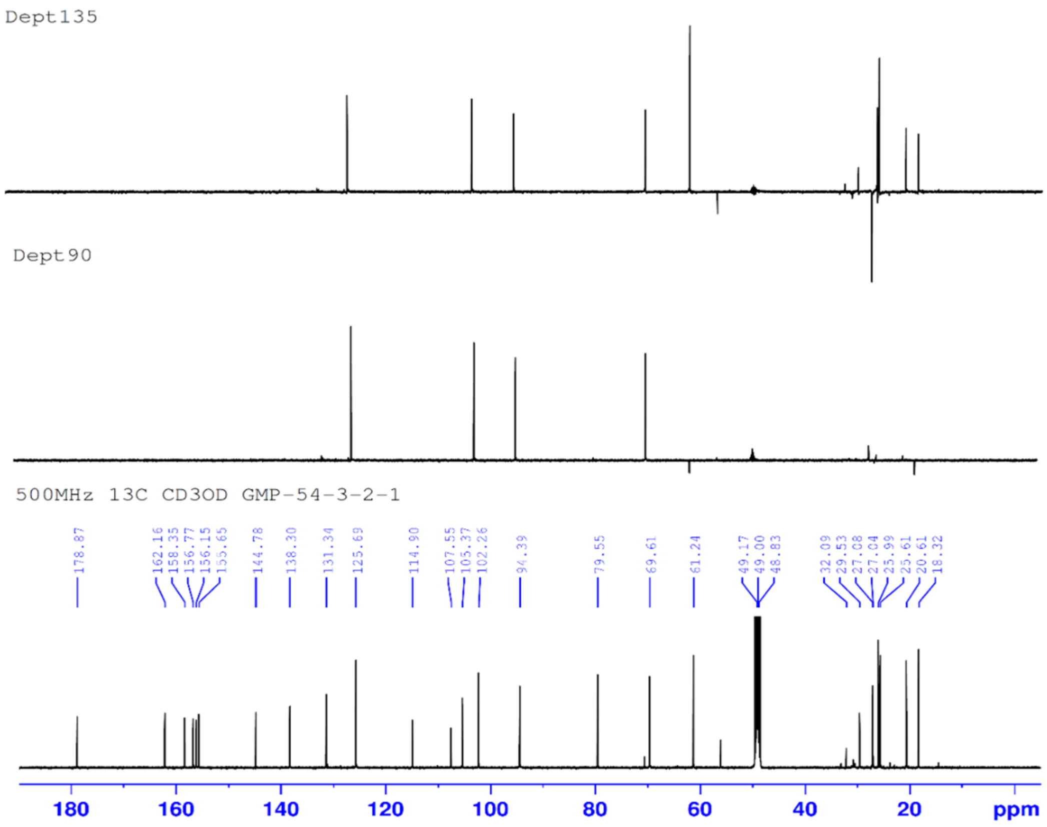

Figure S28. DEPT spectrum of compound **3**.

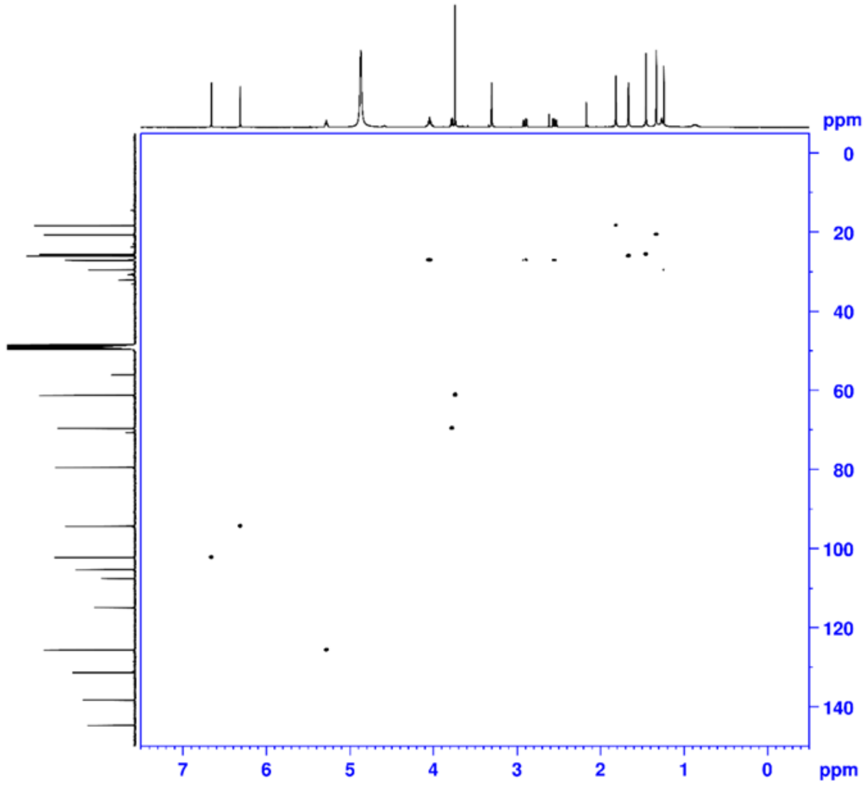

Figure S29. HSQC spectrum of compound **3**.

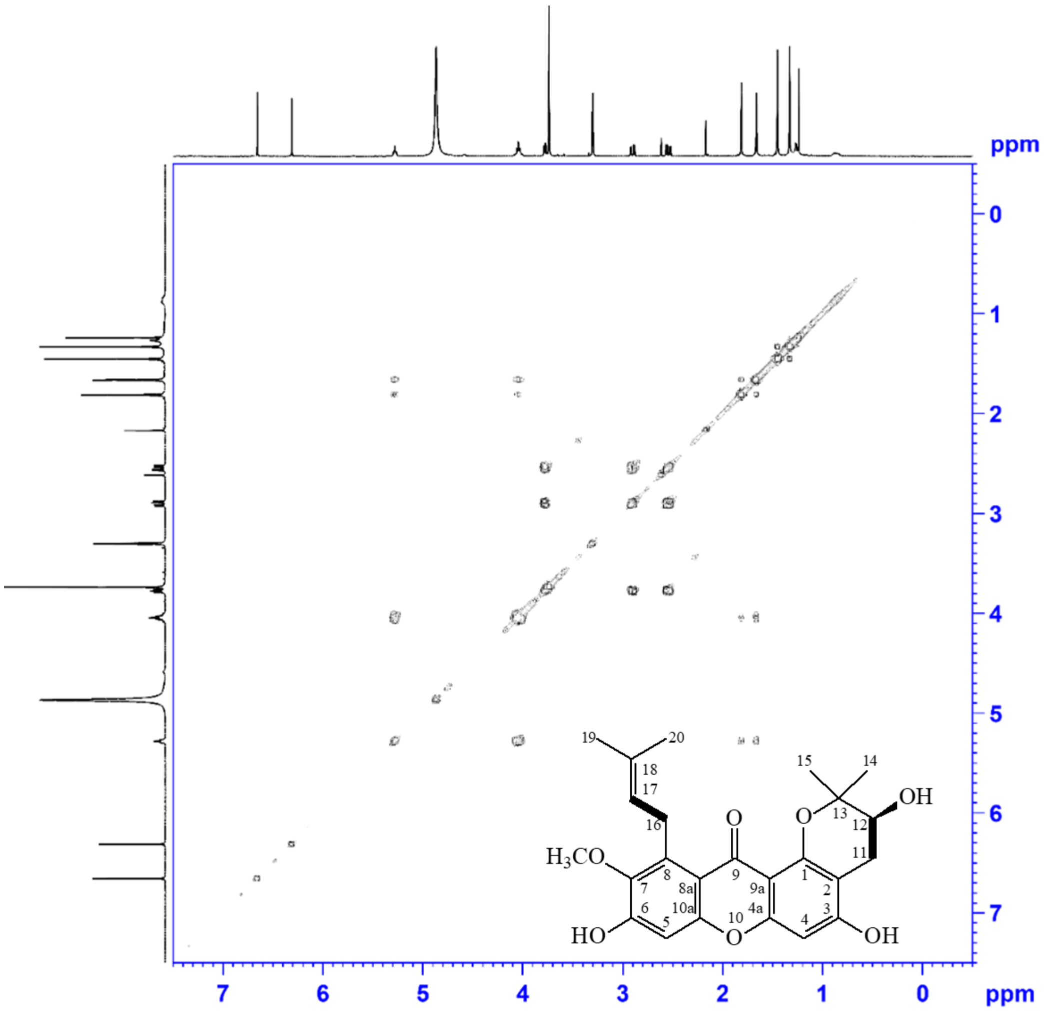

Figure S30. ^{1}H-^{1}H COSY spectrum of compound **3**.

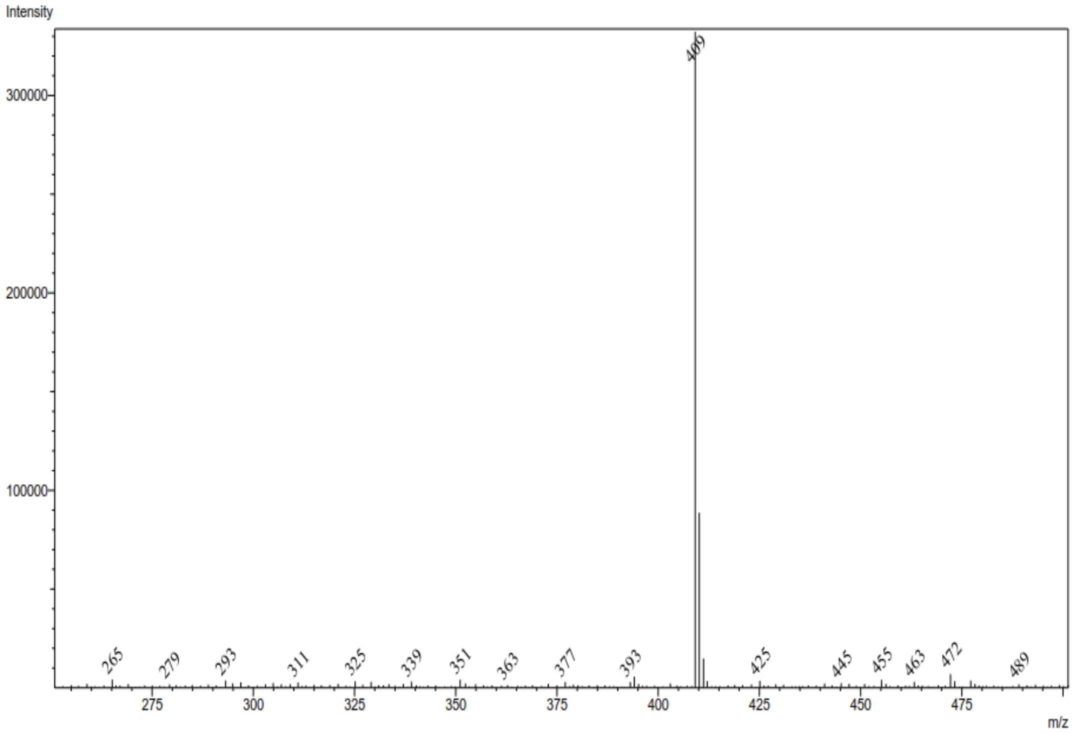

Figure S31. ESI-MS spectrum of compound **4**.

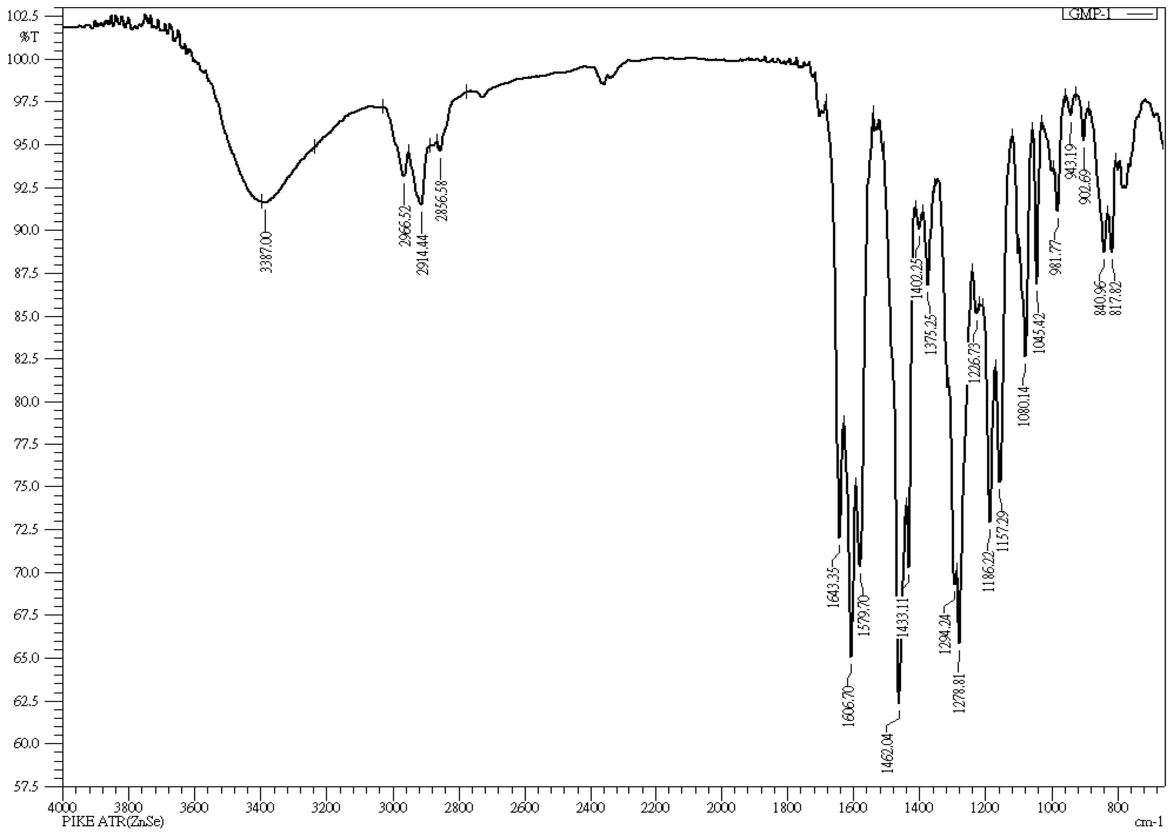

Figure S32. IR spectrum of compound **4**.

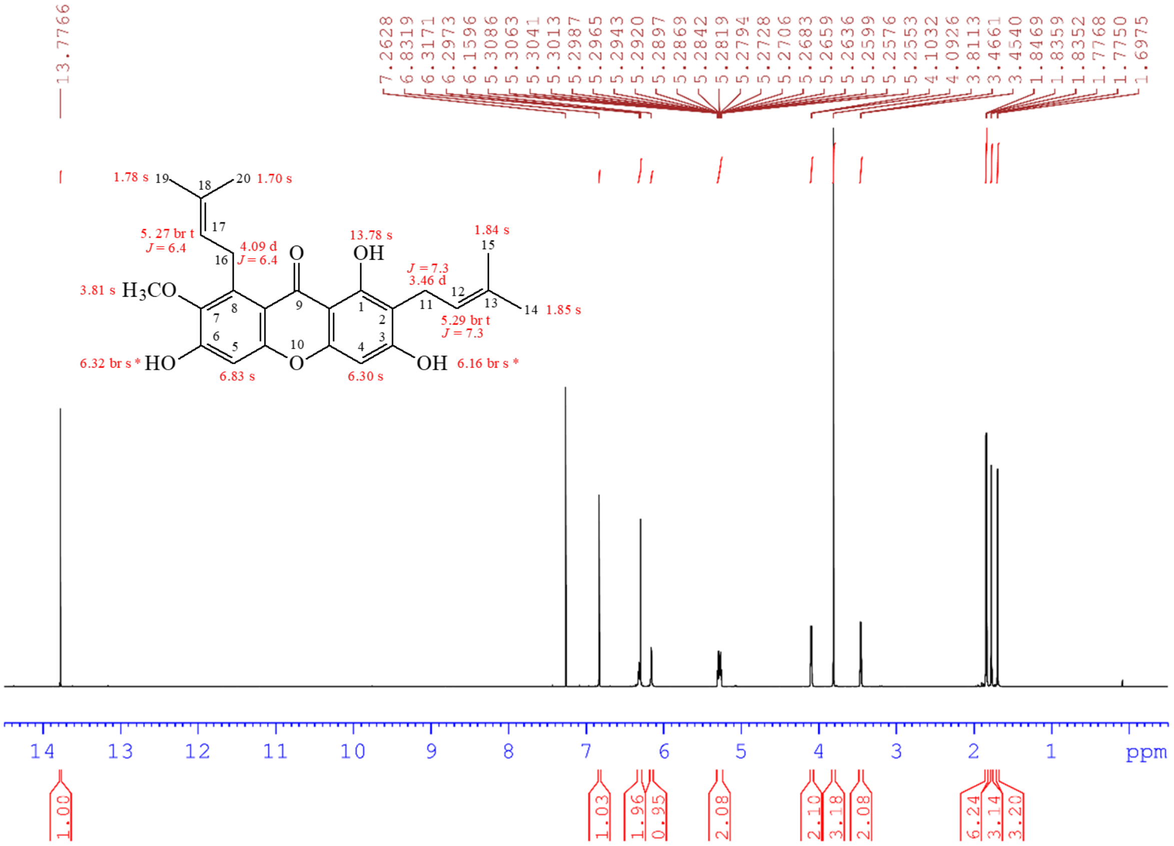

Figure S33. ^{1}H-NMR spectrum of compound **4**.

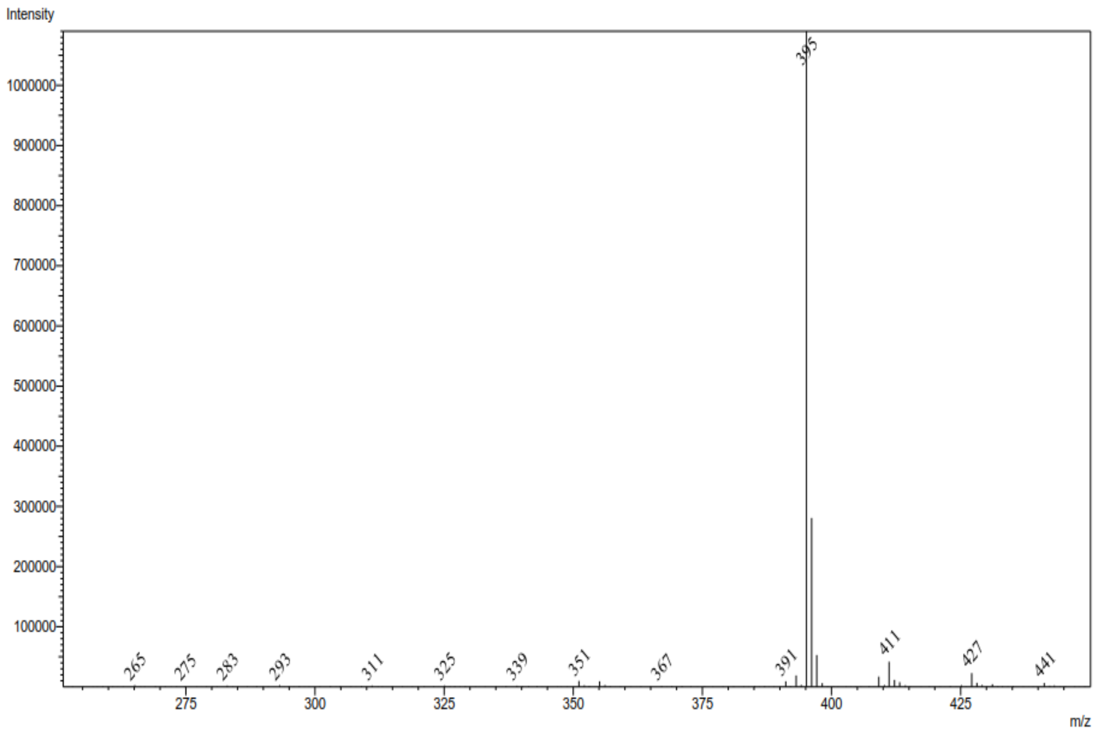

Figure S34. ESI-MS spectrum of compound **5**.

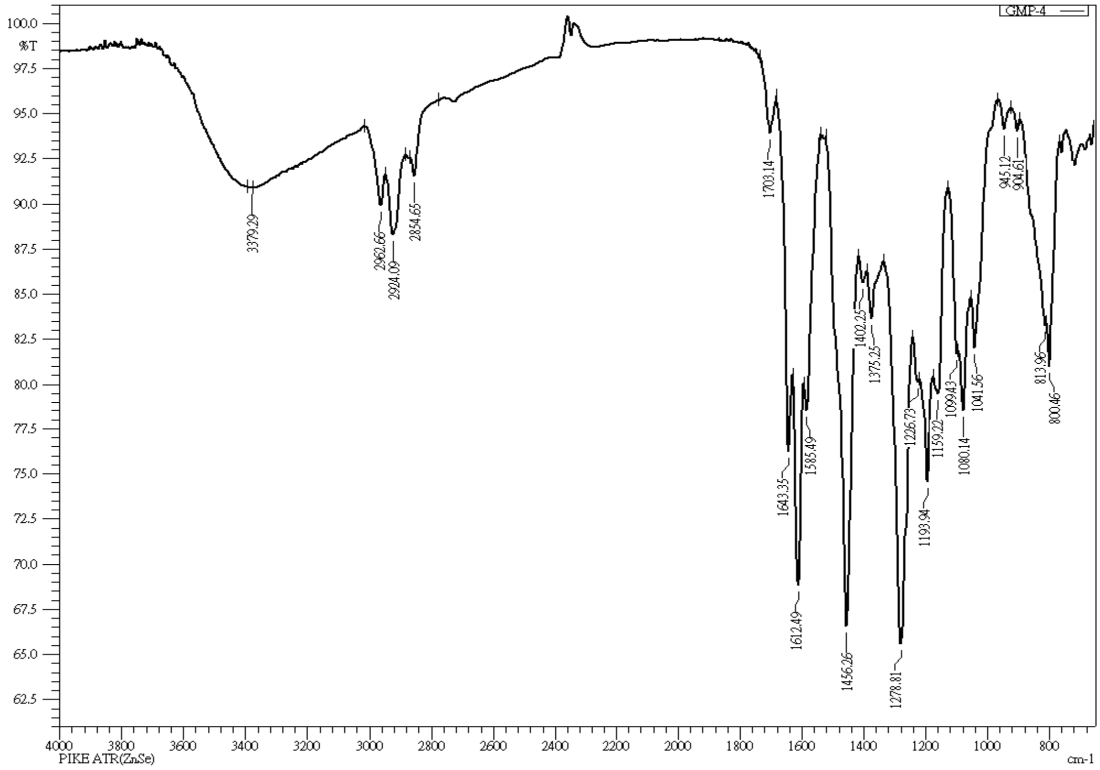

Figure S35. IR spectrum of compound **5**.

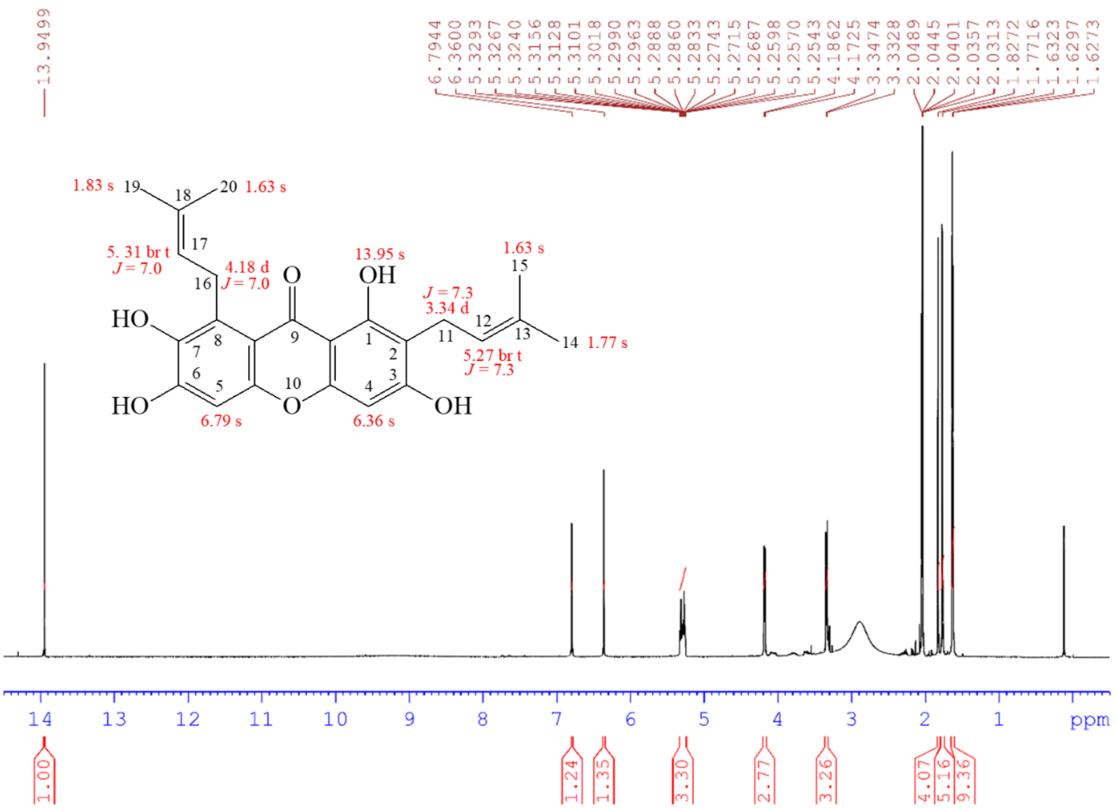

Figure S36. ^{1}H-NMR spectrum of compound **5**.

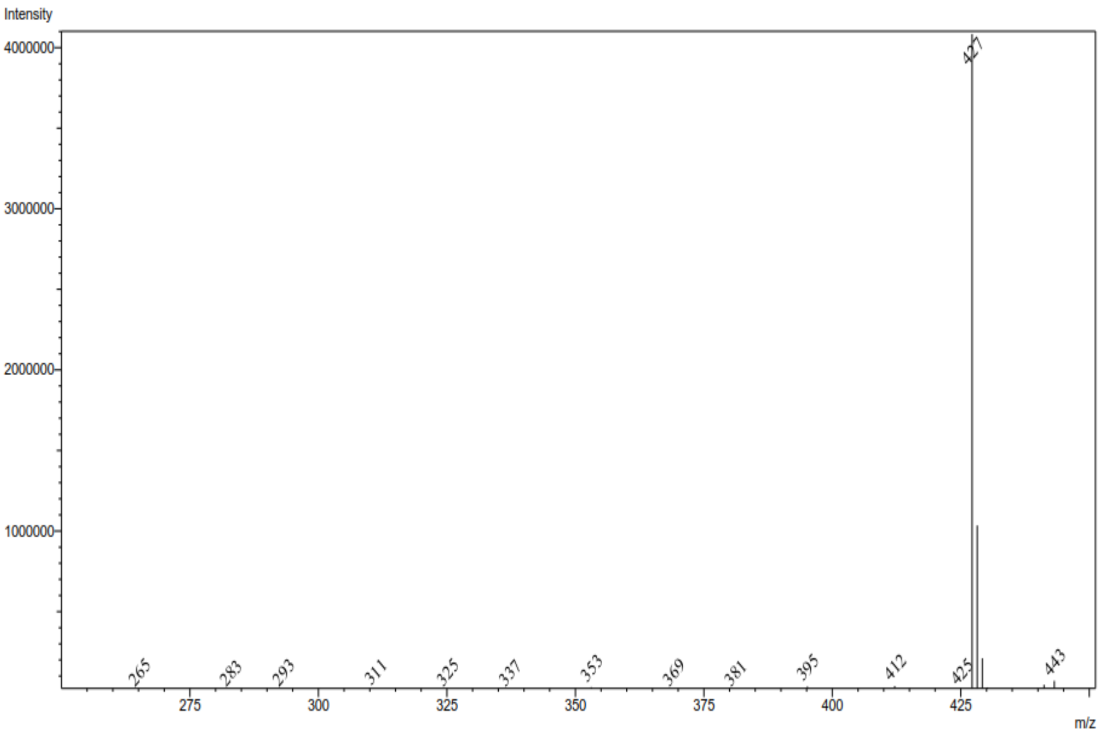

Figure S37. ESI-MS spectrum of compound **6**.

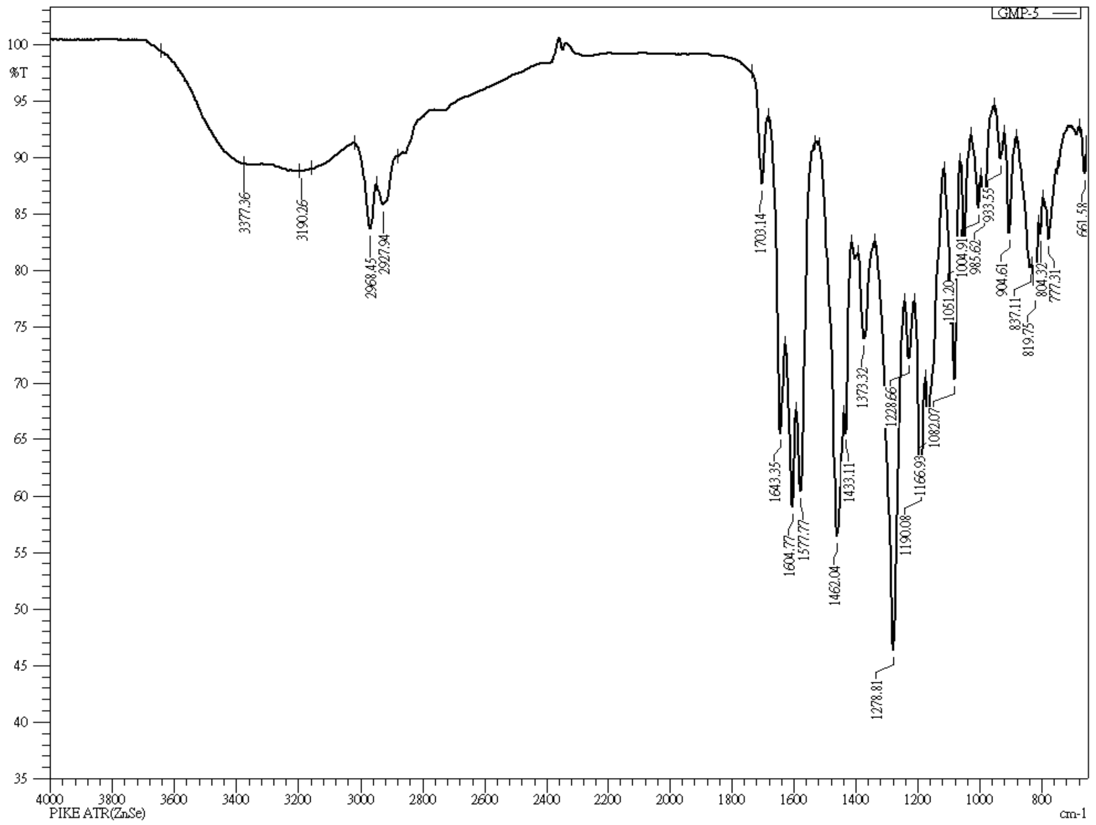

Figure S38. IR spectrum of compound **6**.

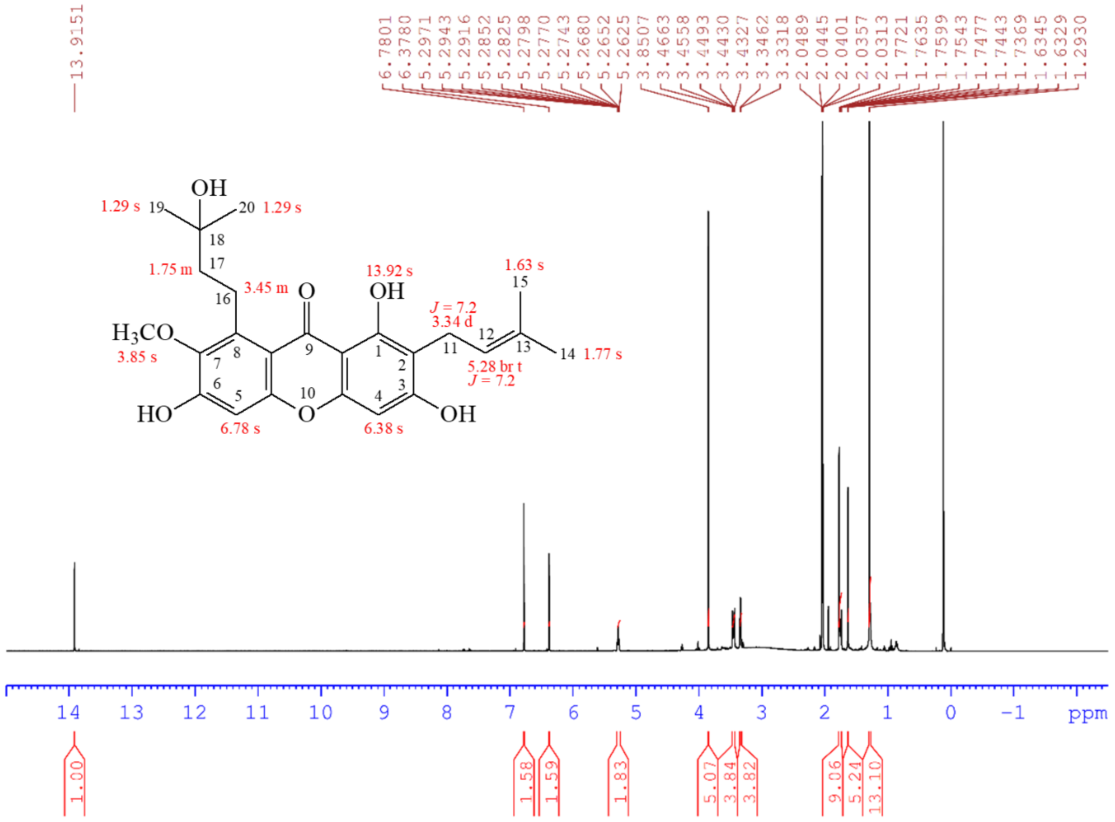

Figure S39. ^{1}H-NMR spectrum of compound **6**.

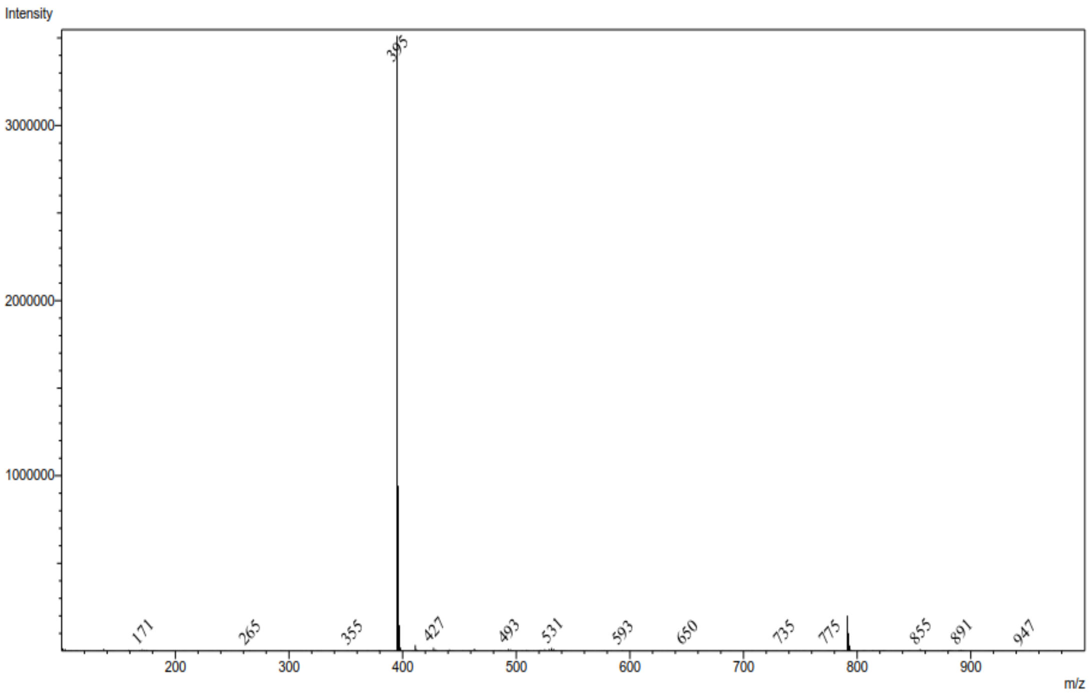

Figure S40. ESI-MS spectrum of compound **7**.

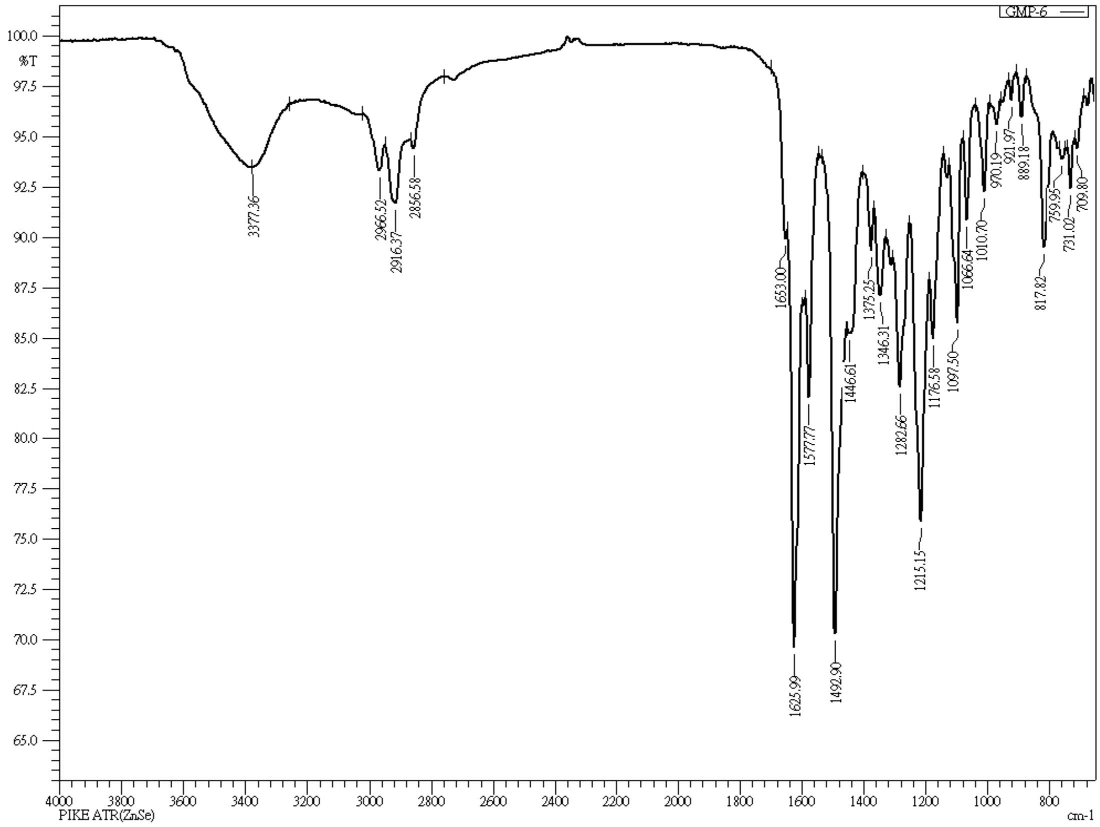

Figure S41. IR spectrum of compound **7**.

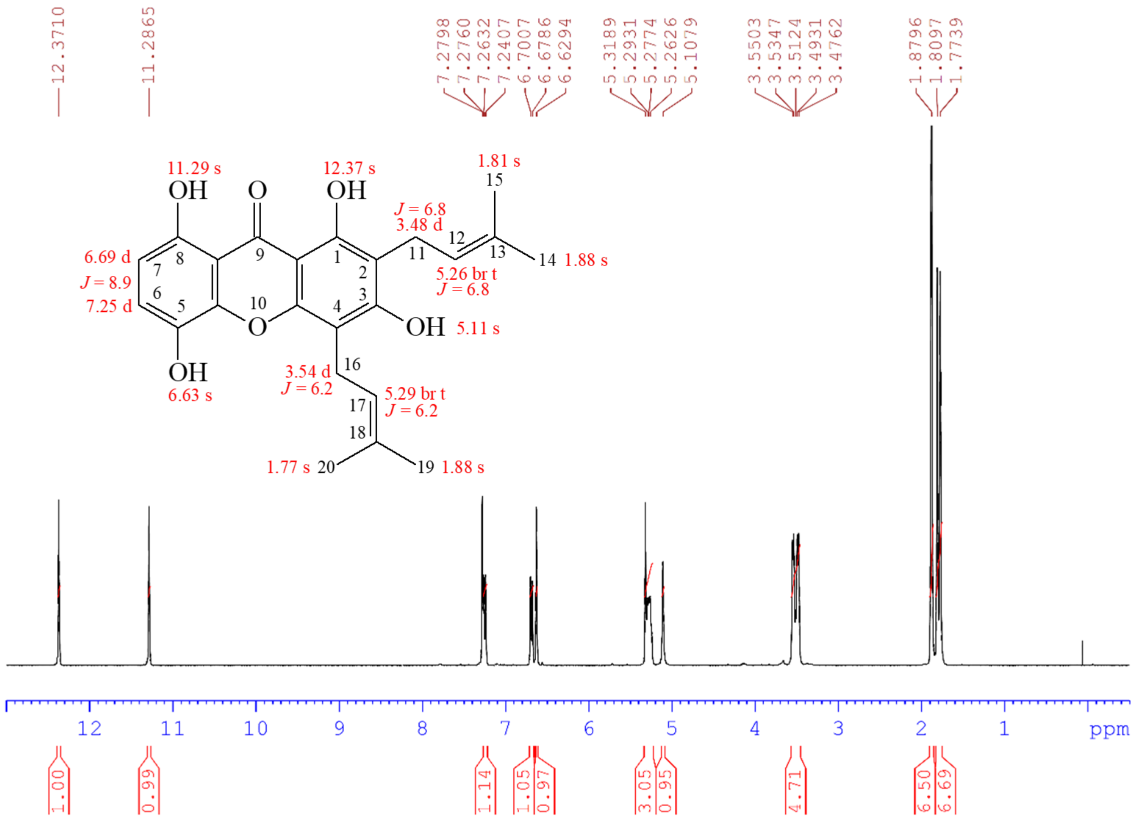

Figure S42. ^{1}H-NMR spectrum of compound **7**.

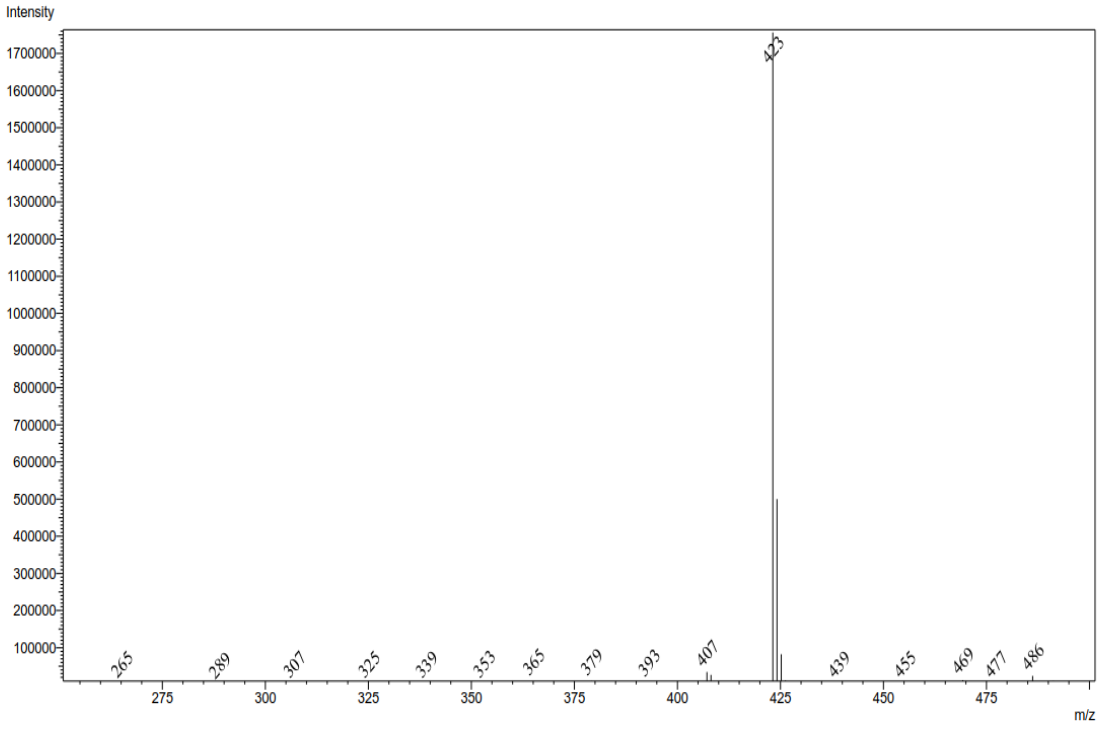

Figure S43. ESI-MS spectrum of compound **8**.

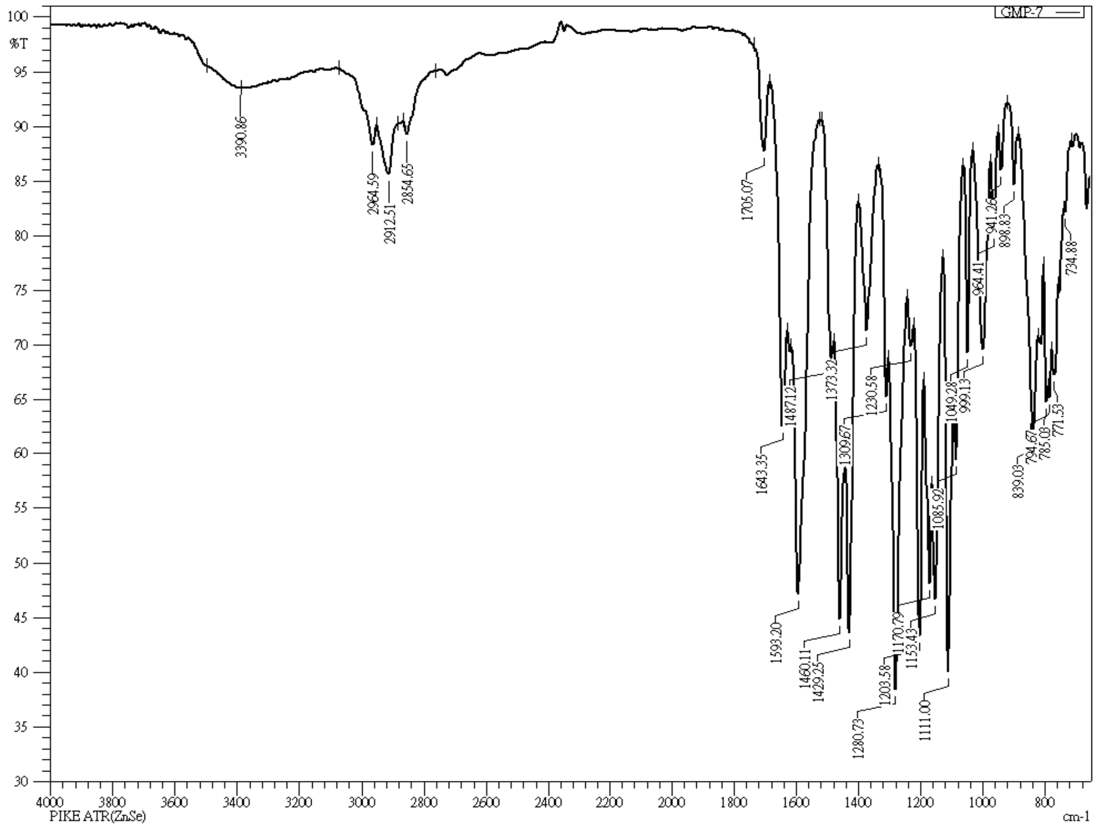

Figure S44. IR spectrum of compound **8**.

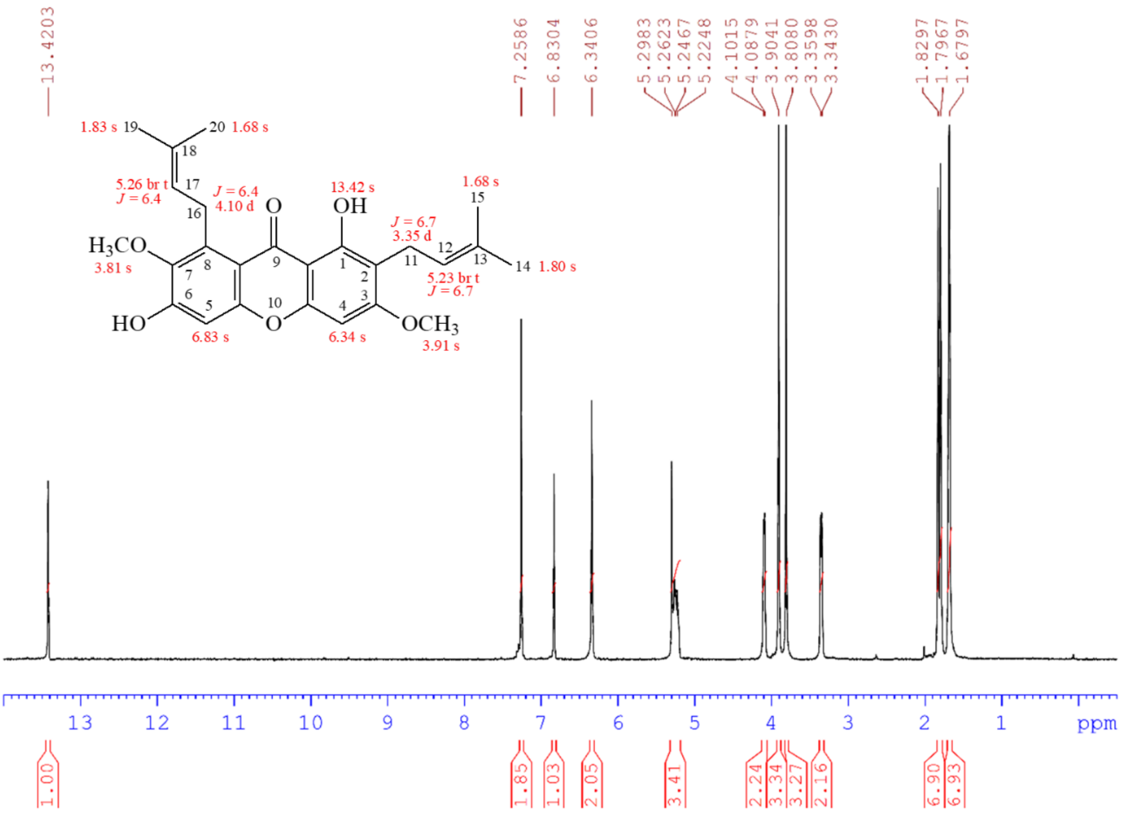

Figure S45. ^{1}H-NMR spectrum of compound **8**.

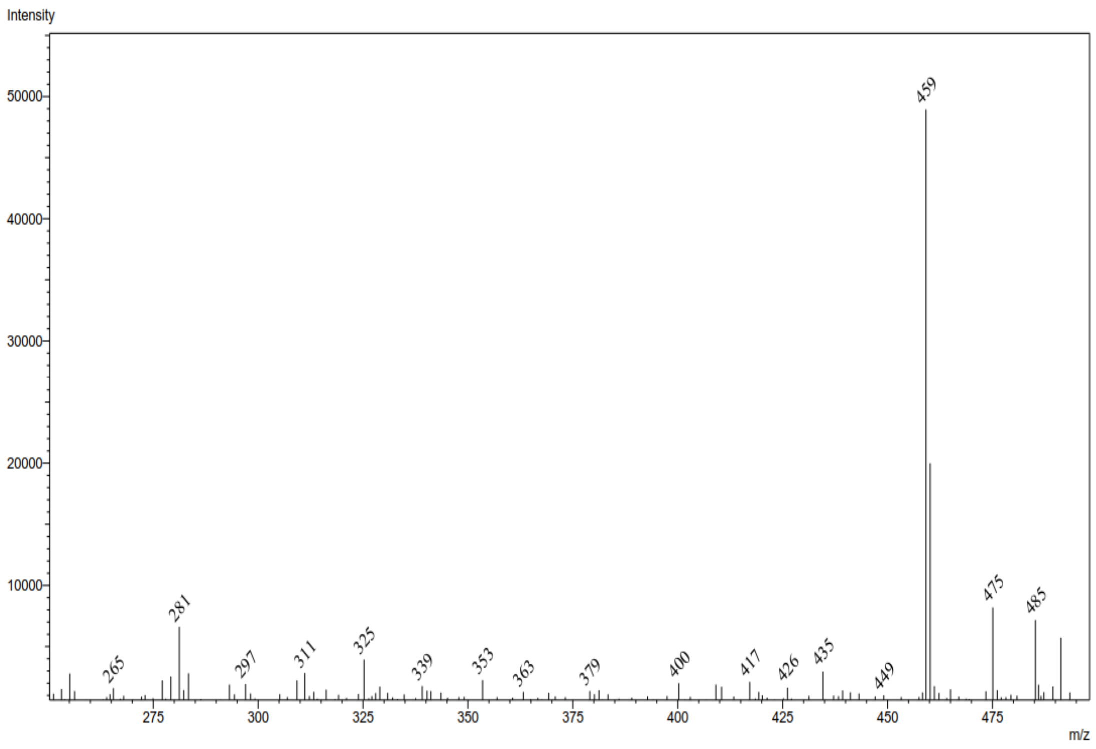

Figure S46. ESI-MS spectrum of compound 9.

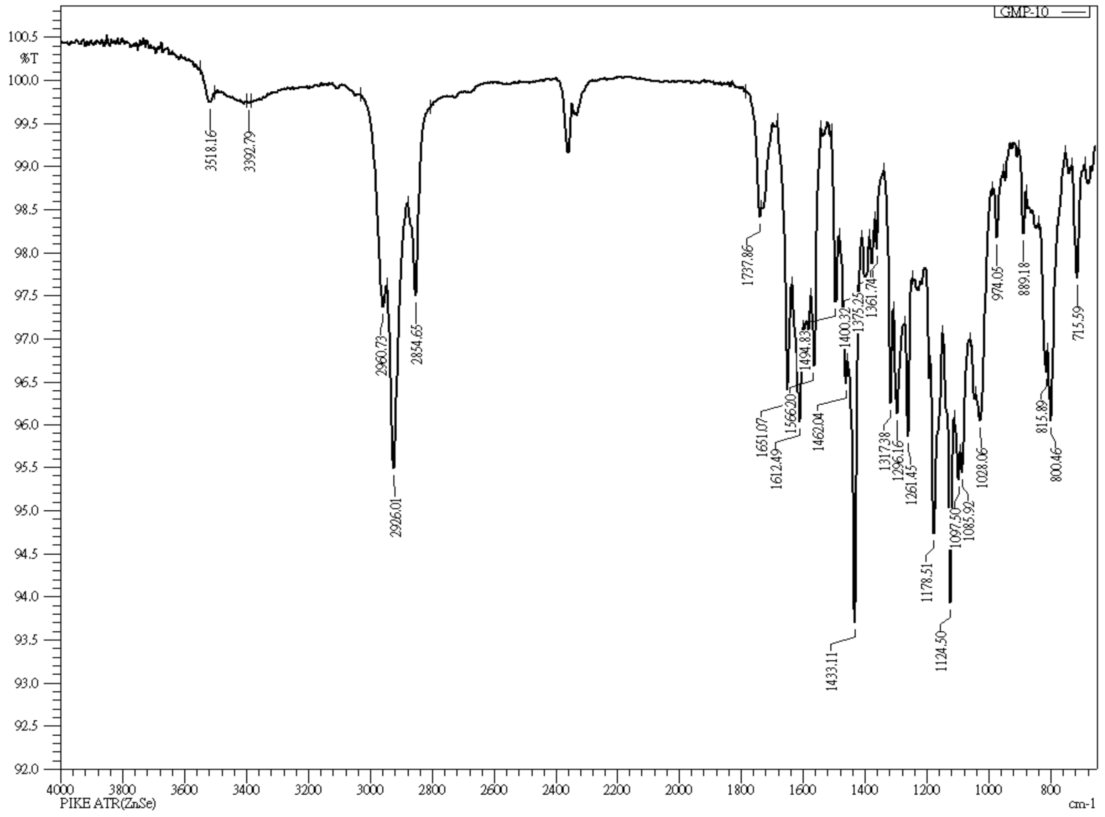

Figure S47. IR spectrum of compound 9.

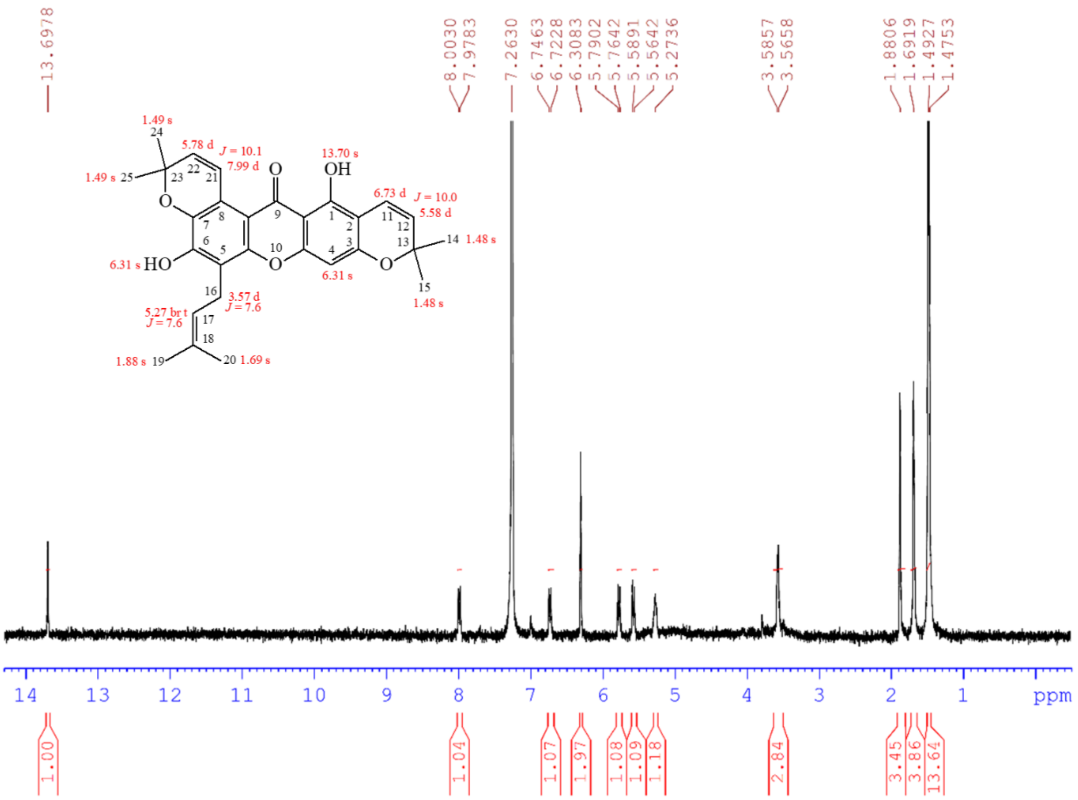

Figure S48. ^{1}H-NMR spectrum of compound **9**.

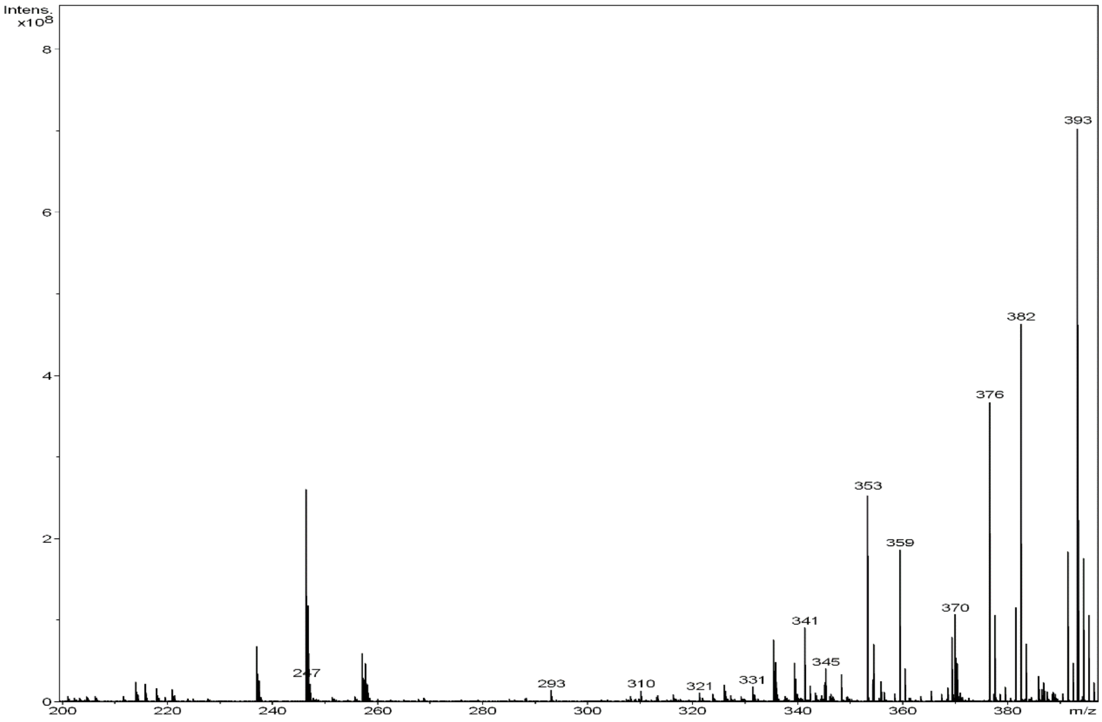

Figure S49. ESI-MS spectrum of compound **10**.

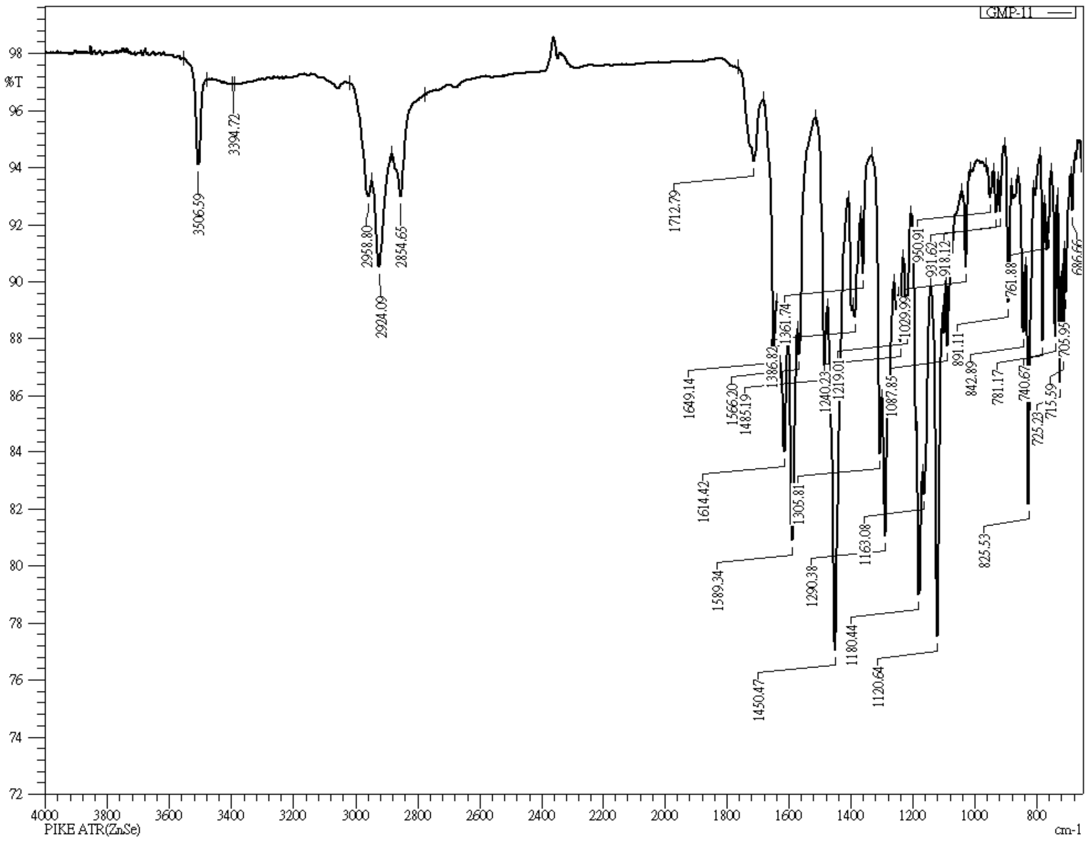

Figure S50. IR spectrum of compound **10**.

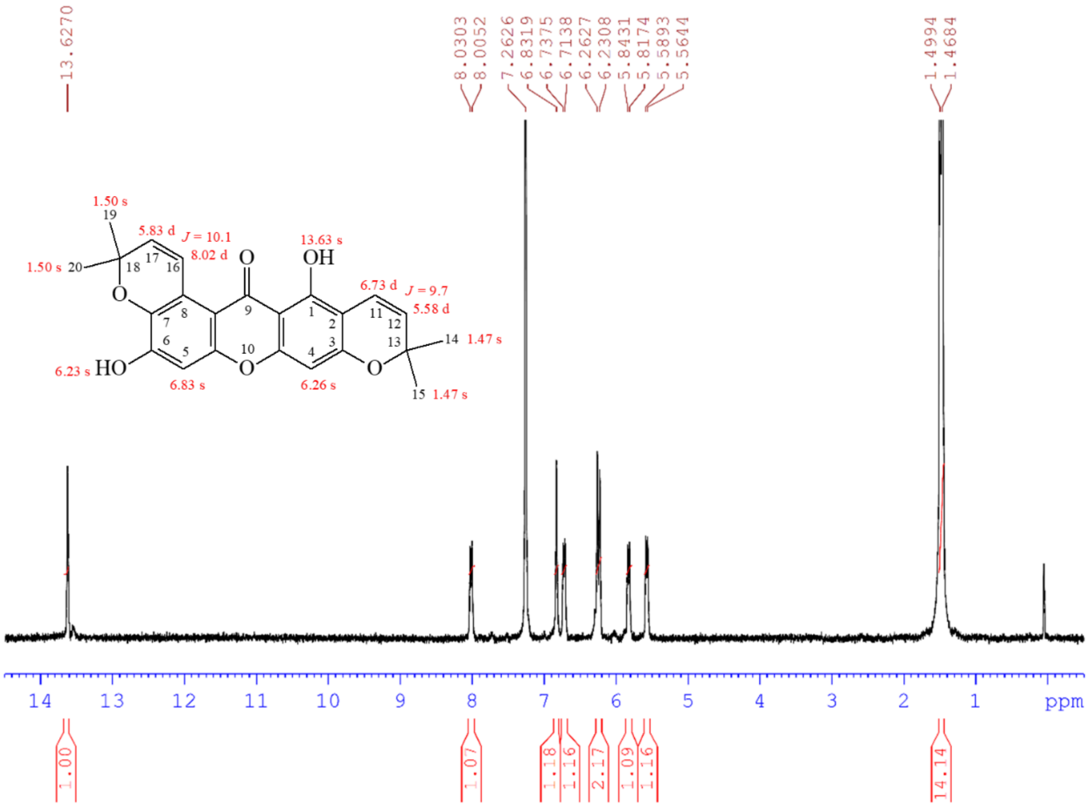

Figure S51. ^{1}H-NMR spectrum of compound **10**.

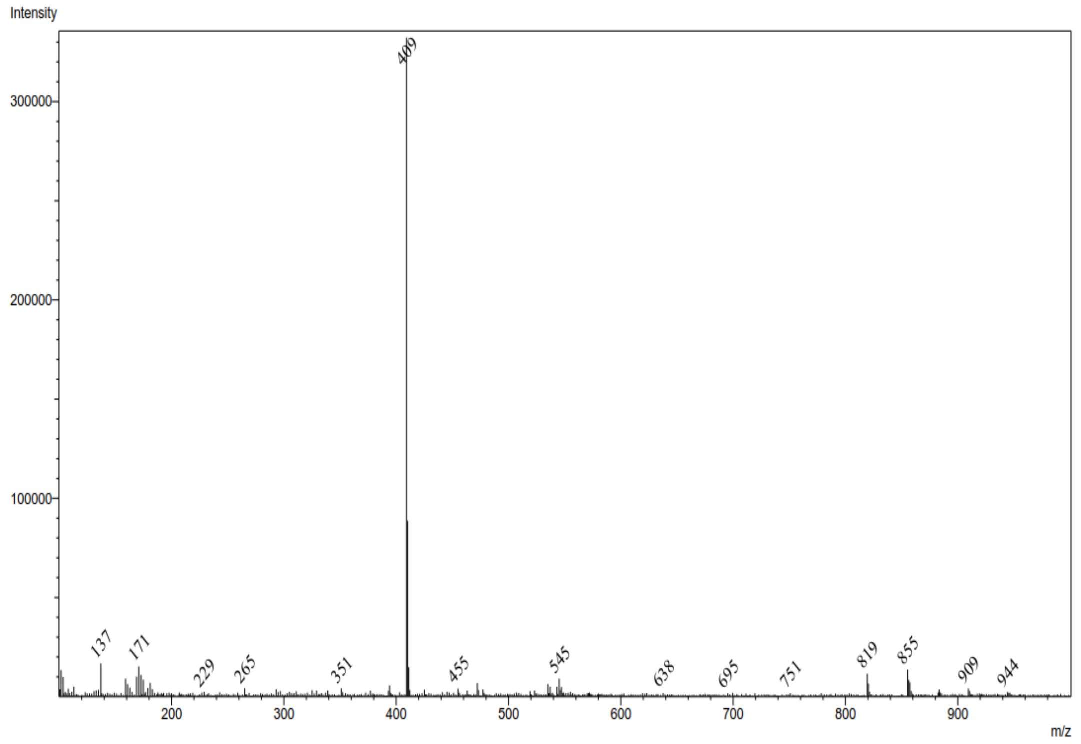

Figure S52. ESI-MS spectrum of compound **11**.

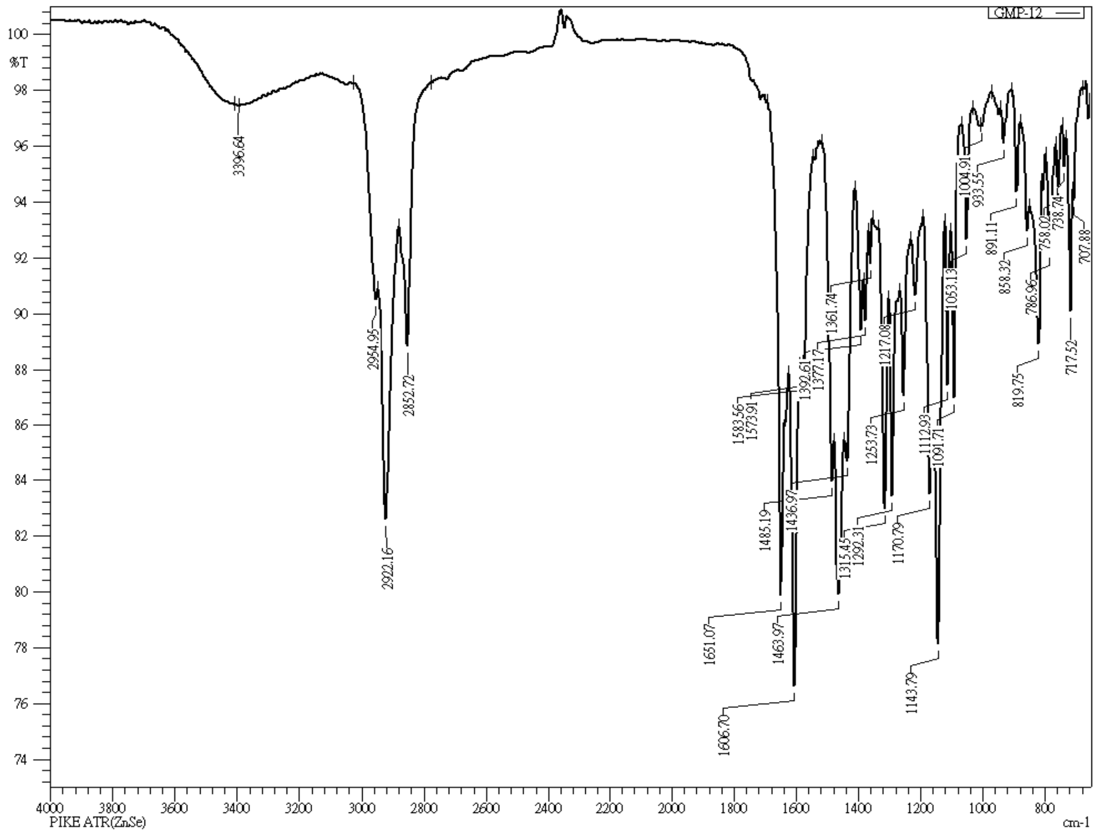

Figure S53. IR spectrum of compound **11**.

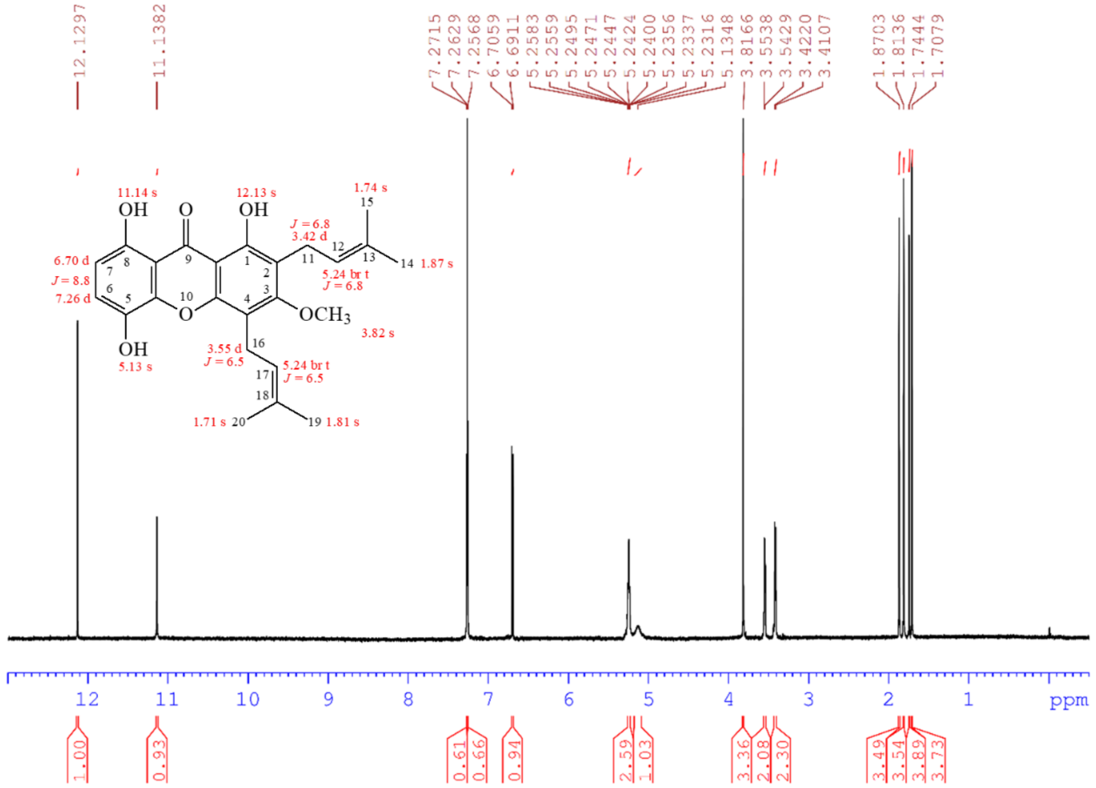

Figure S54. ¹H-NMR spectrum of compound **11**.

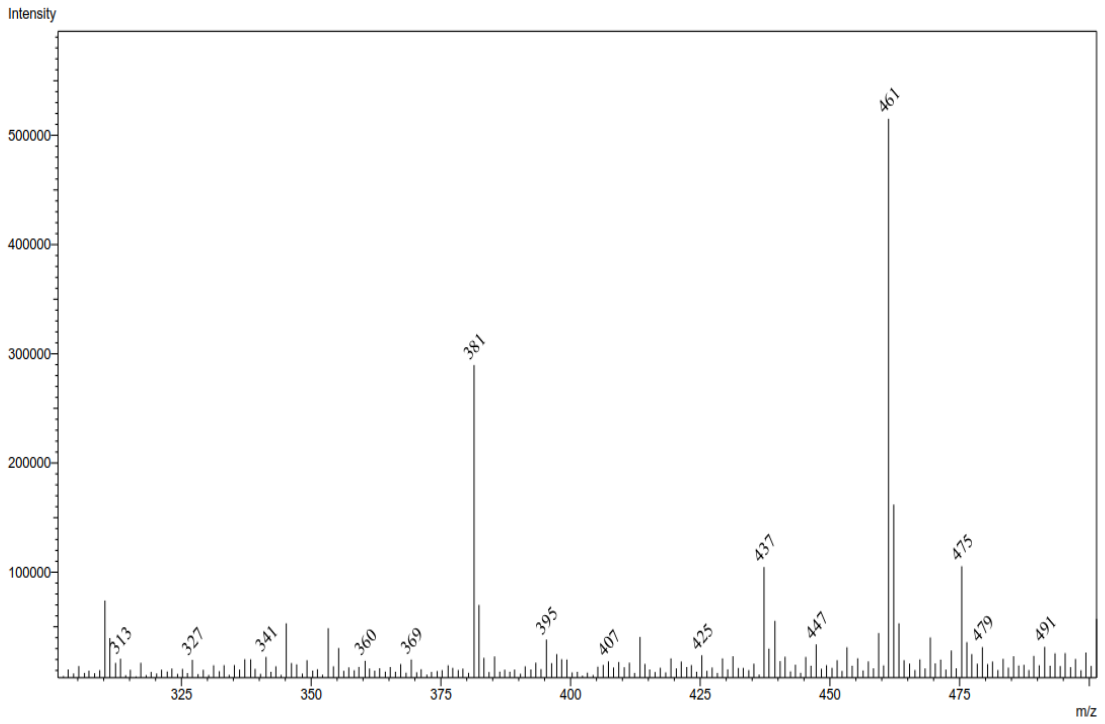

Figure S55. ESI-MS spectrum of compound **12**.

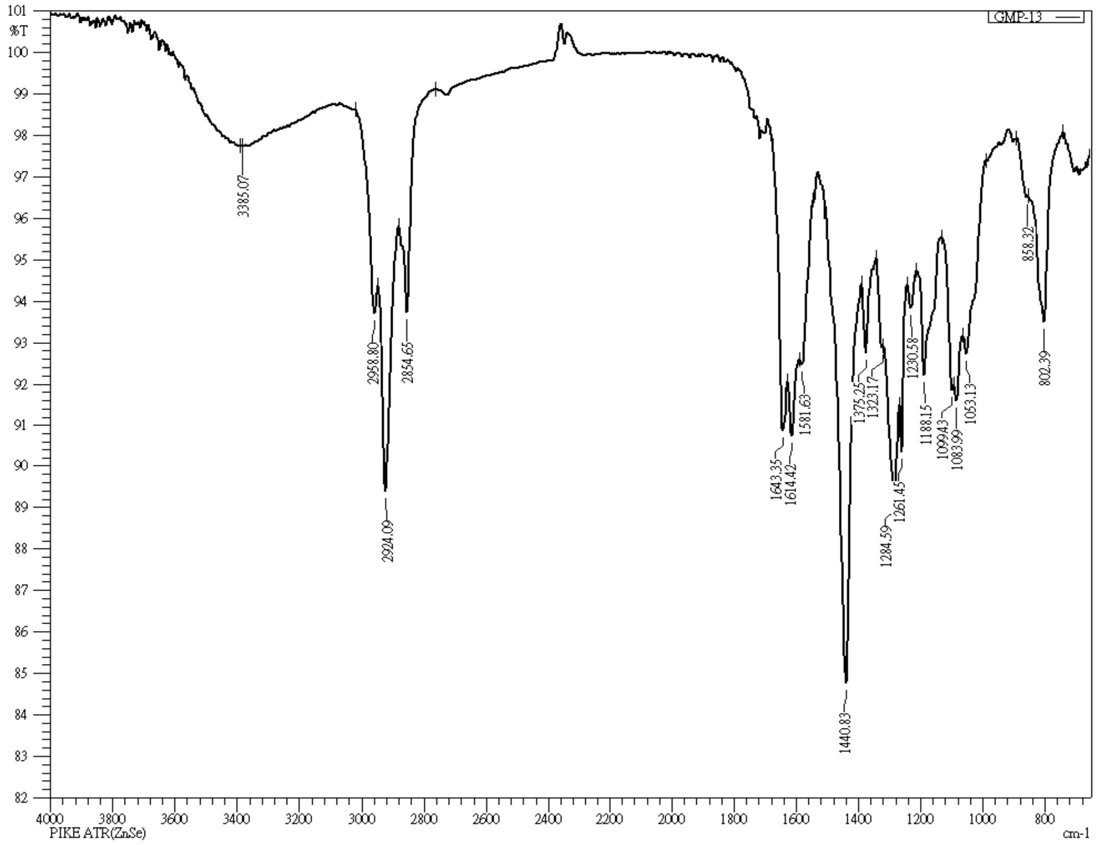

Figure S56. IR spectrum of compound **12**.

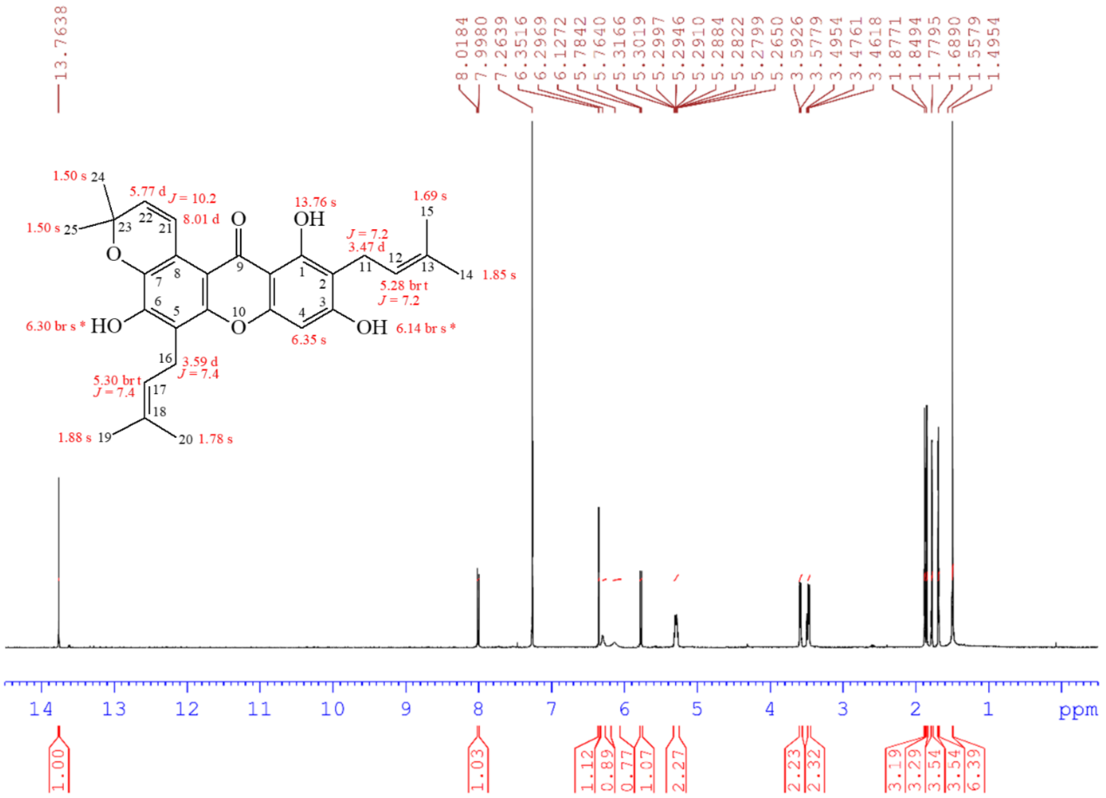

Figure S57. ^{1}H-NMR spectrum of compound **12**.

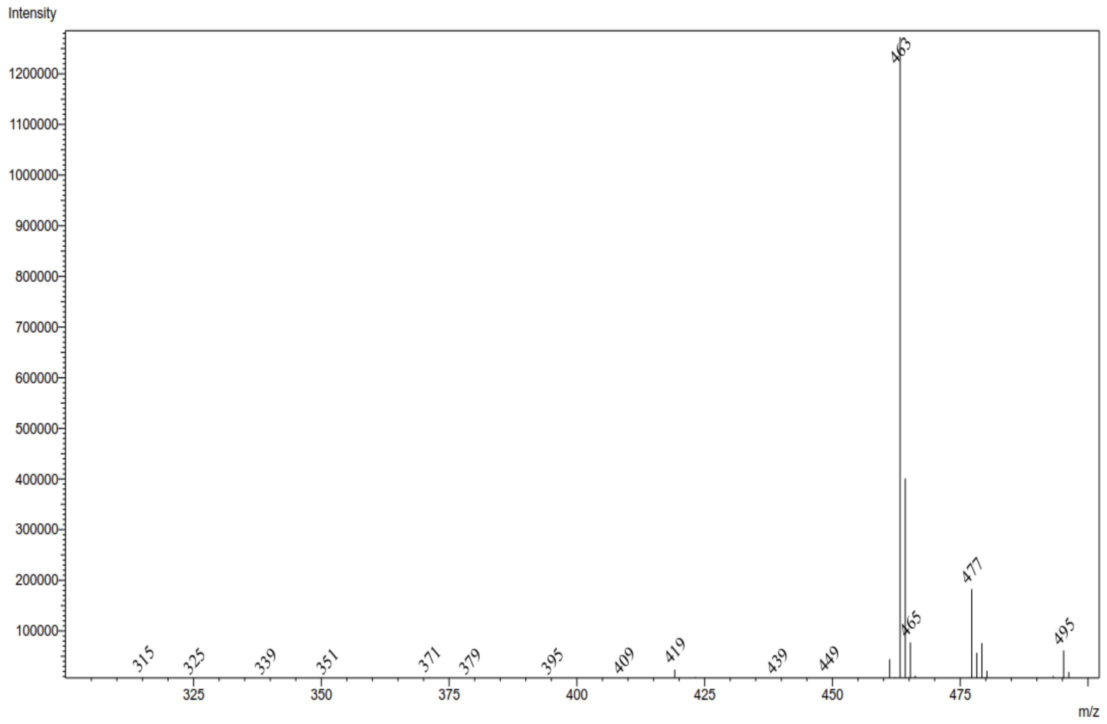

Figure S58. ESI-MS spectrum of compound **13**.

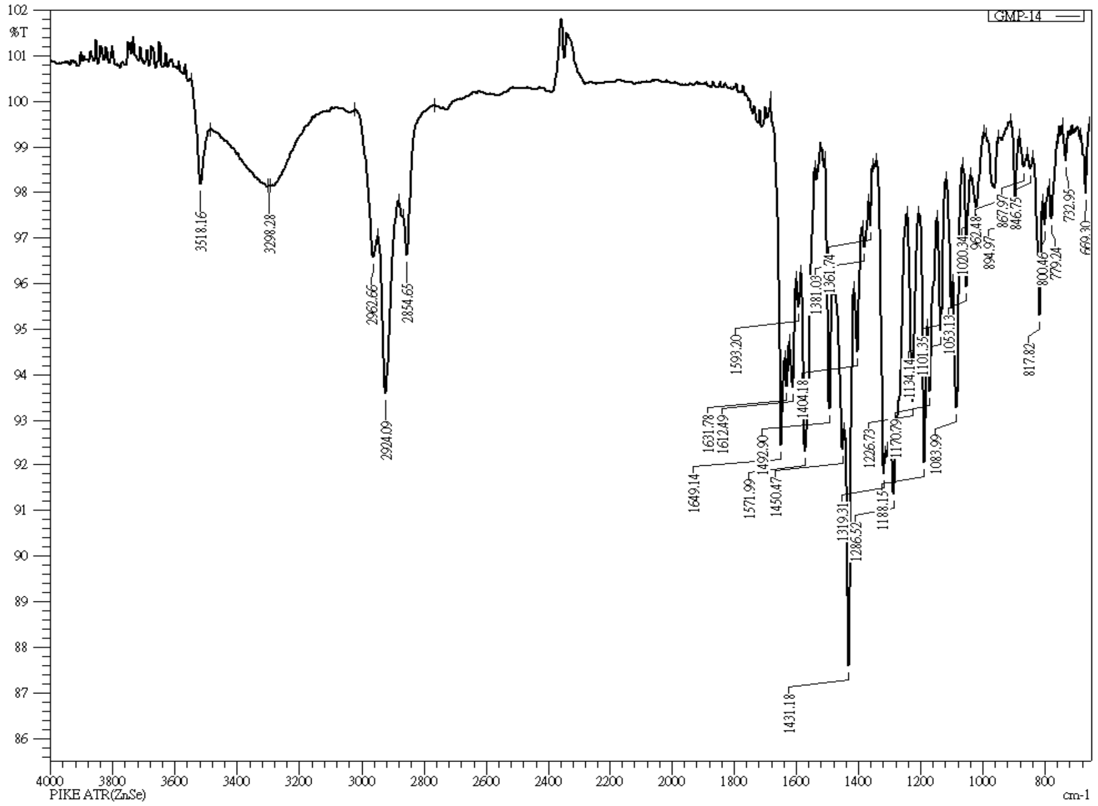

Figure S59. IR spectrum of compound **13**.

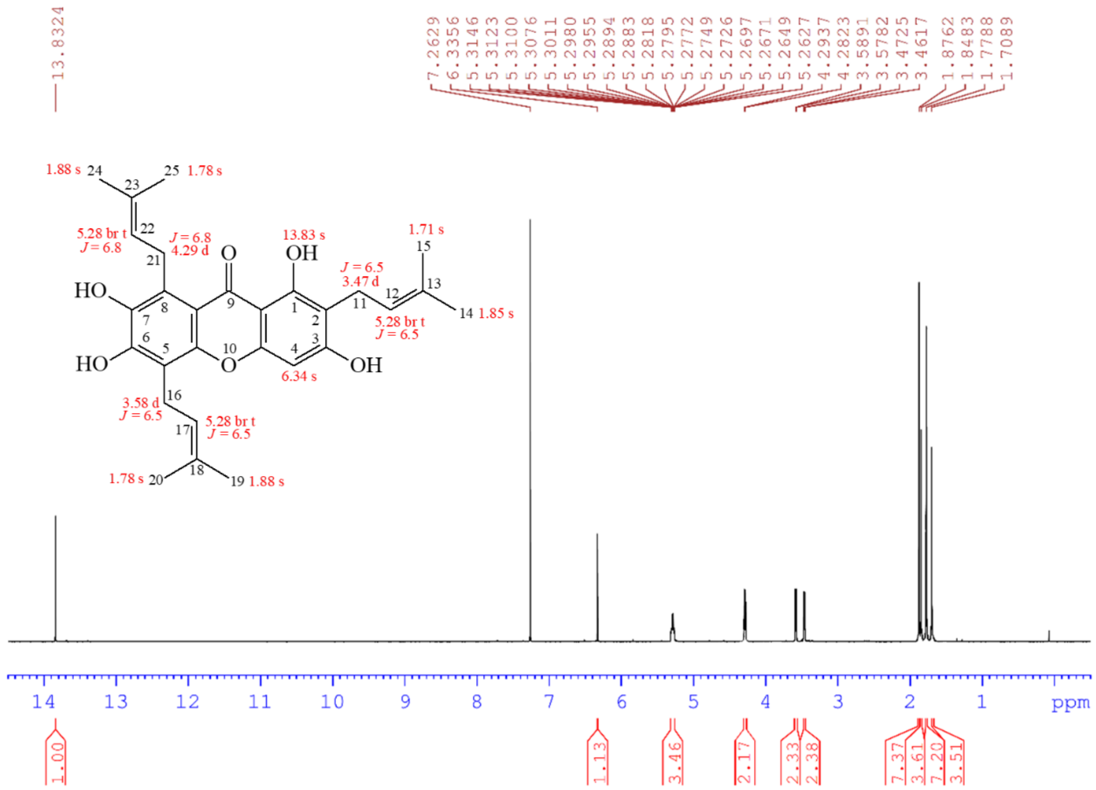

Figure S60. ^{1}H-NMR spectrum of compound **13**.

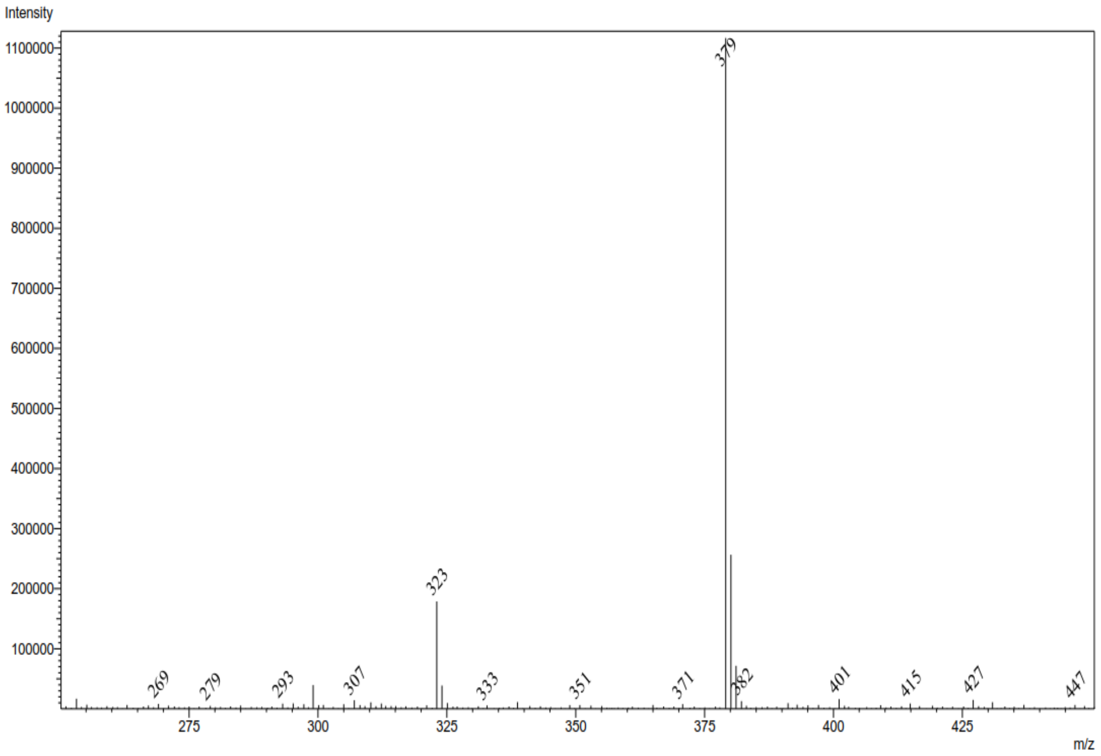

Figure S61. ESI-MS spectrum of compound **14**.

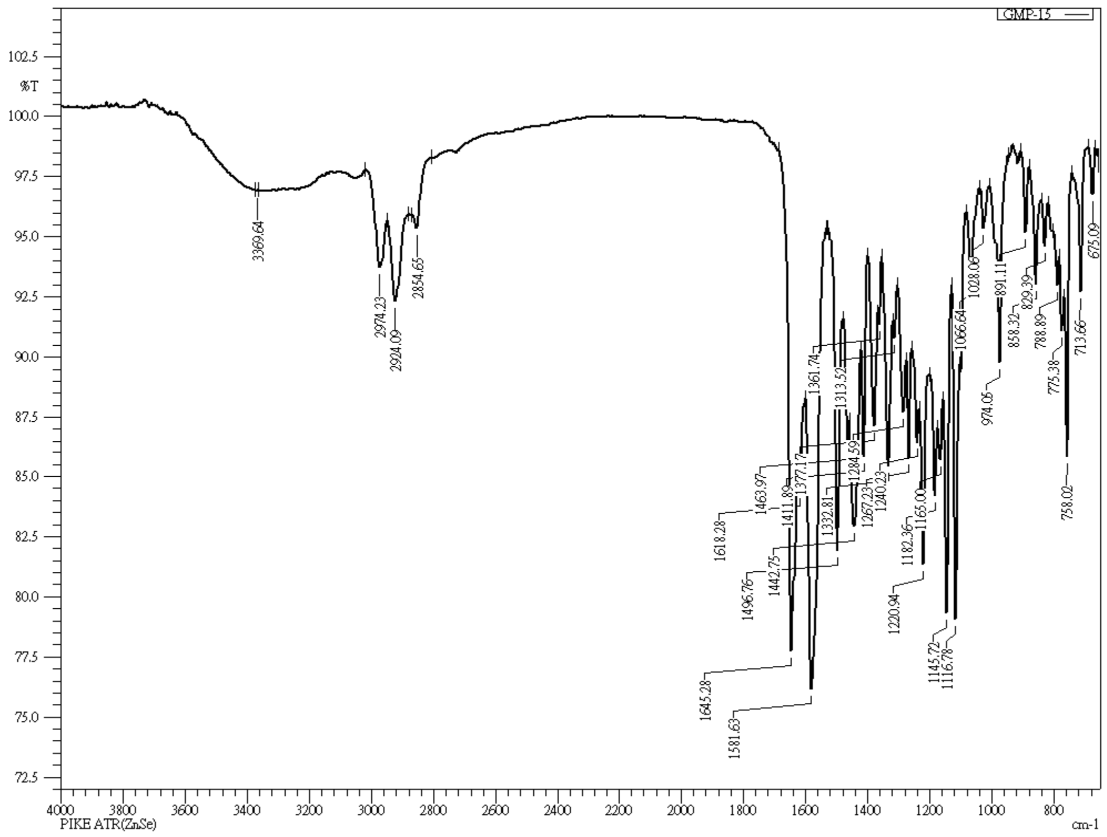

Figure S62. IR spectrum of compound **14**.

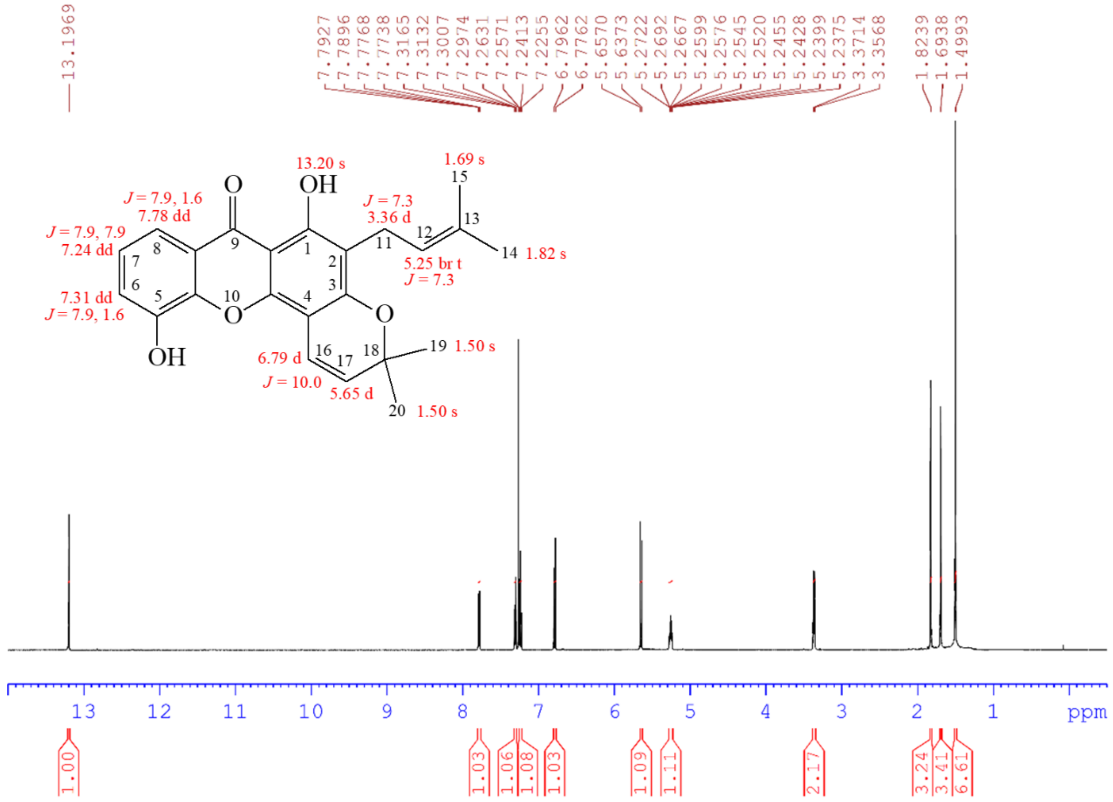

Figure S63. ^{1}H-NMR spectrum of compound **14**.

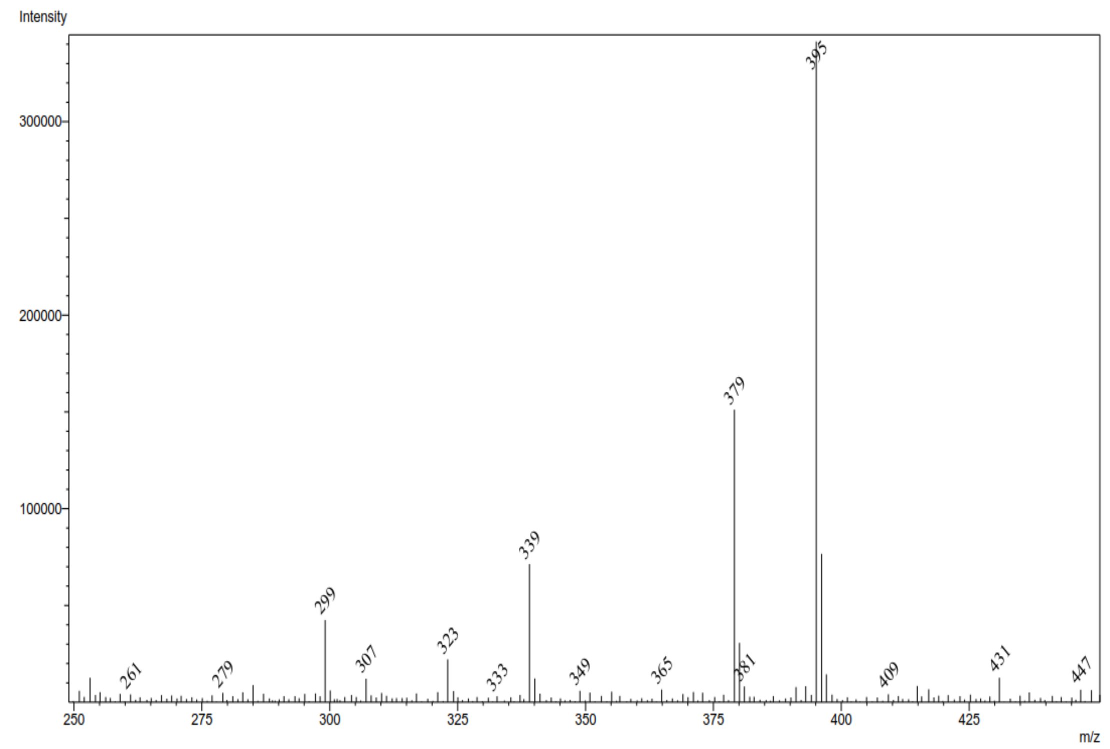

Figure S64. ESI-MS spectrum of compound **15**.

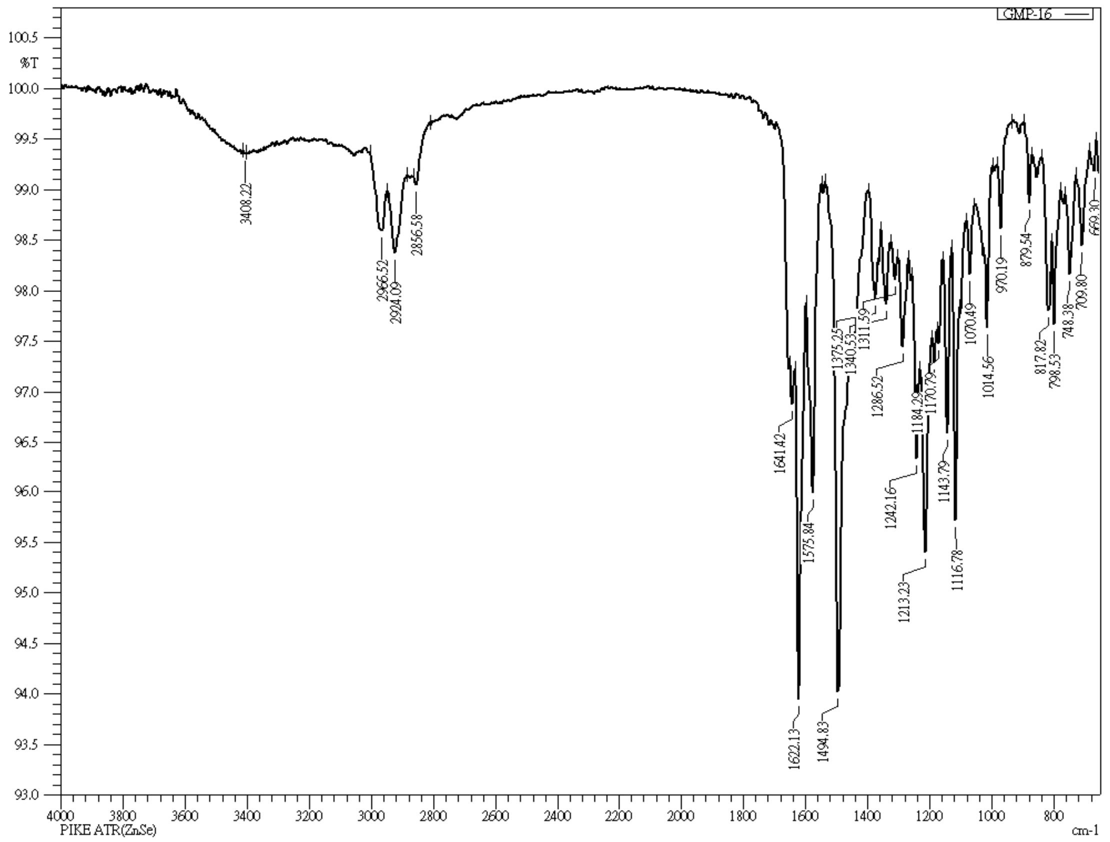

Figure S65. IR spectrum of compound **15**.

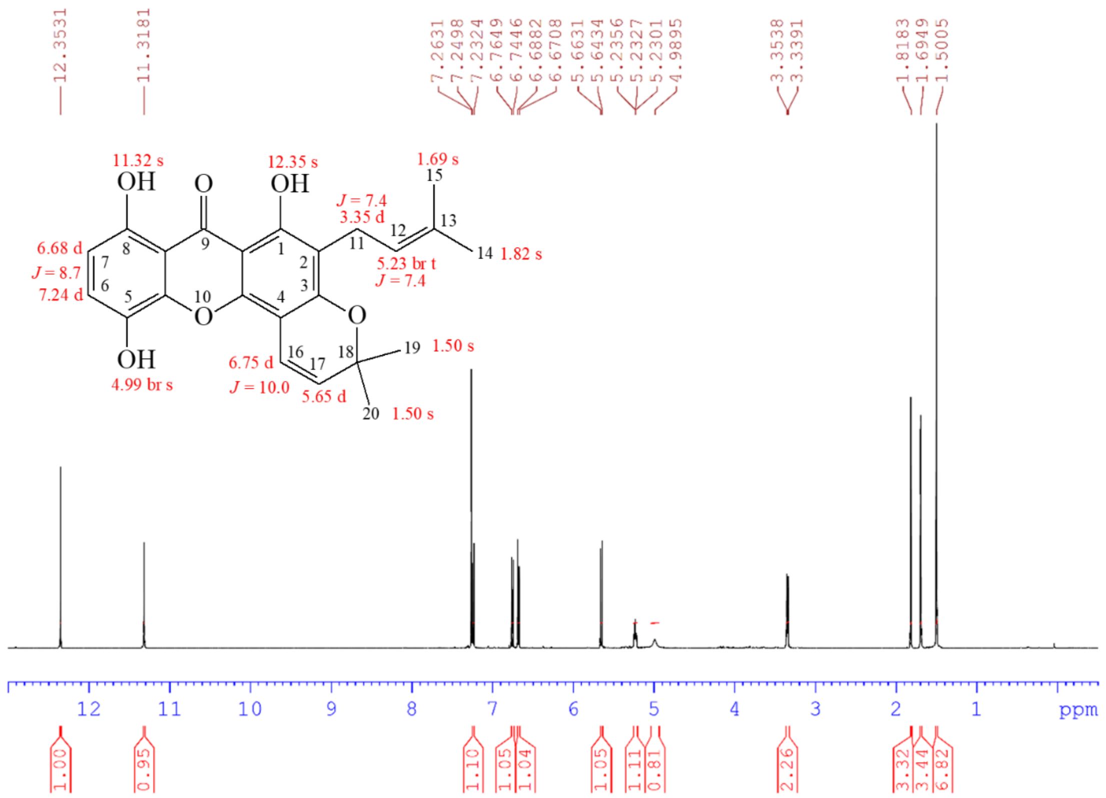

Figure S66. ^{1}H-NMR spectrum of compound **15**.

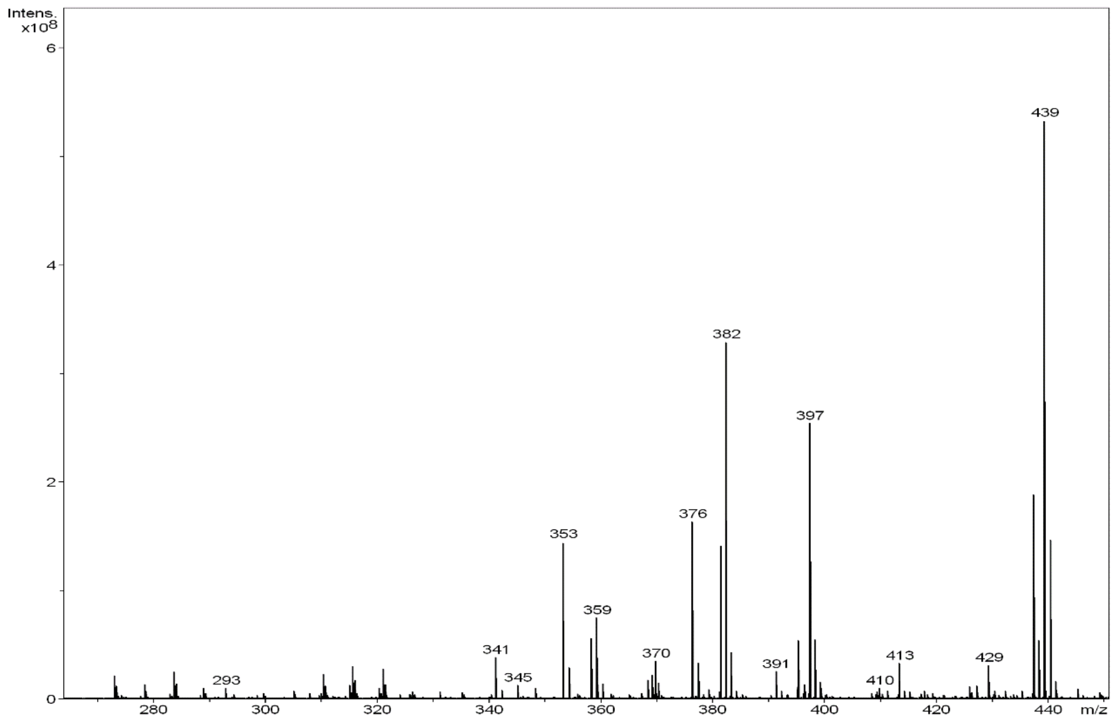

Figure S67. ESI-MS spectrum of compound **16**.

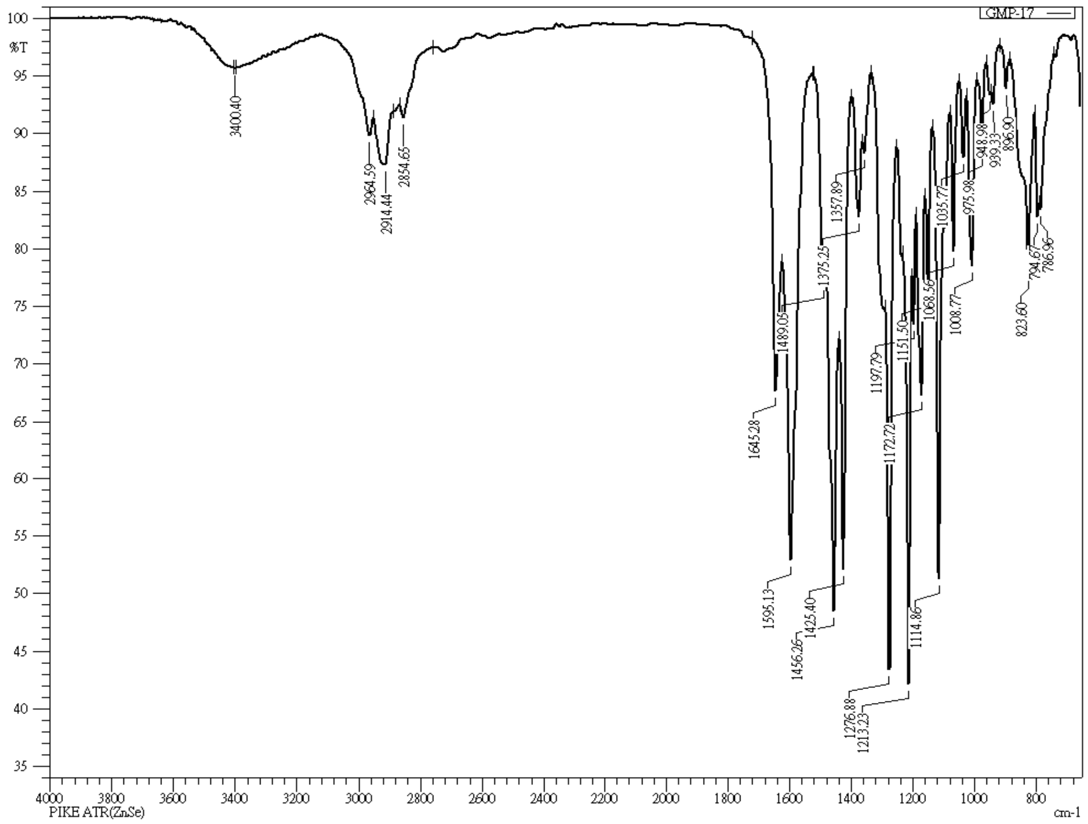

Figure S68. IR spectrum of compound **16**.

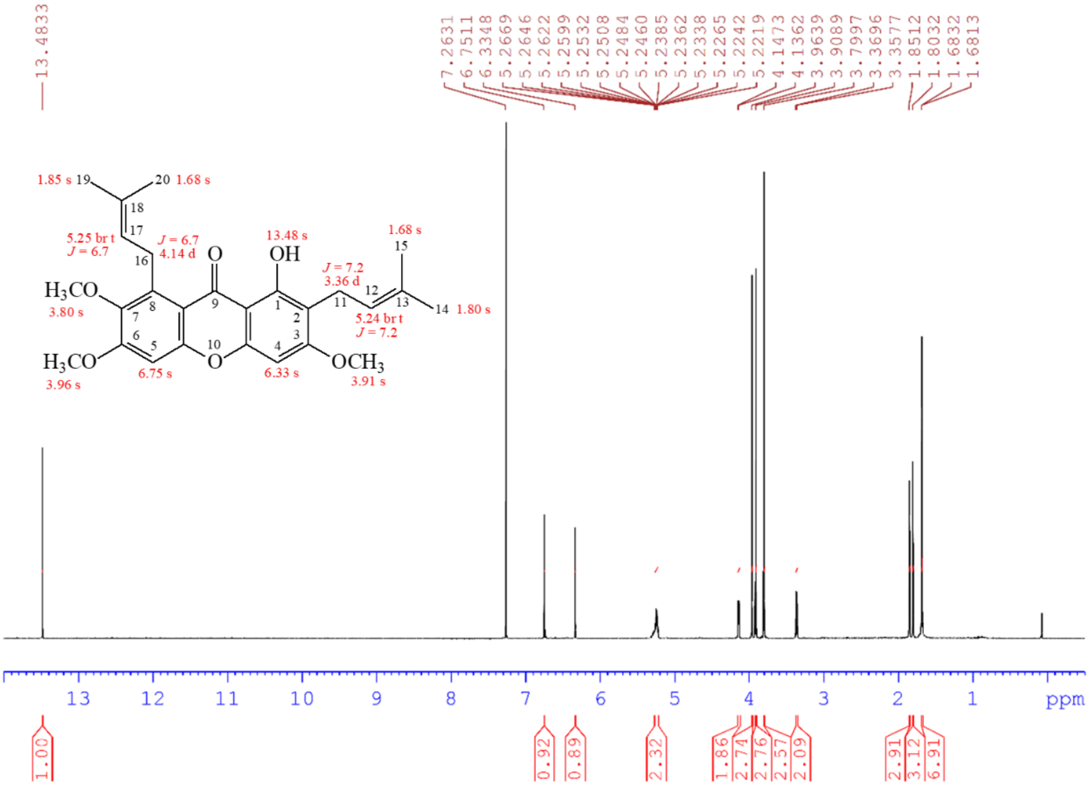

Figure S69. ^{1}H-NMR spectrum of compound **16**.

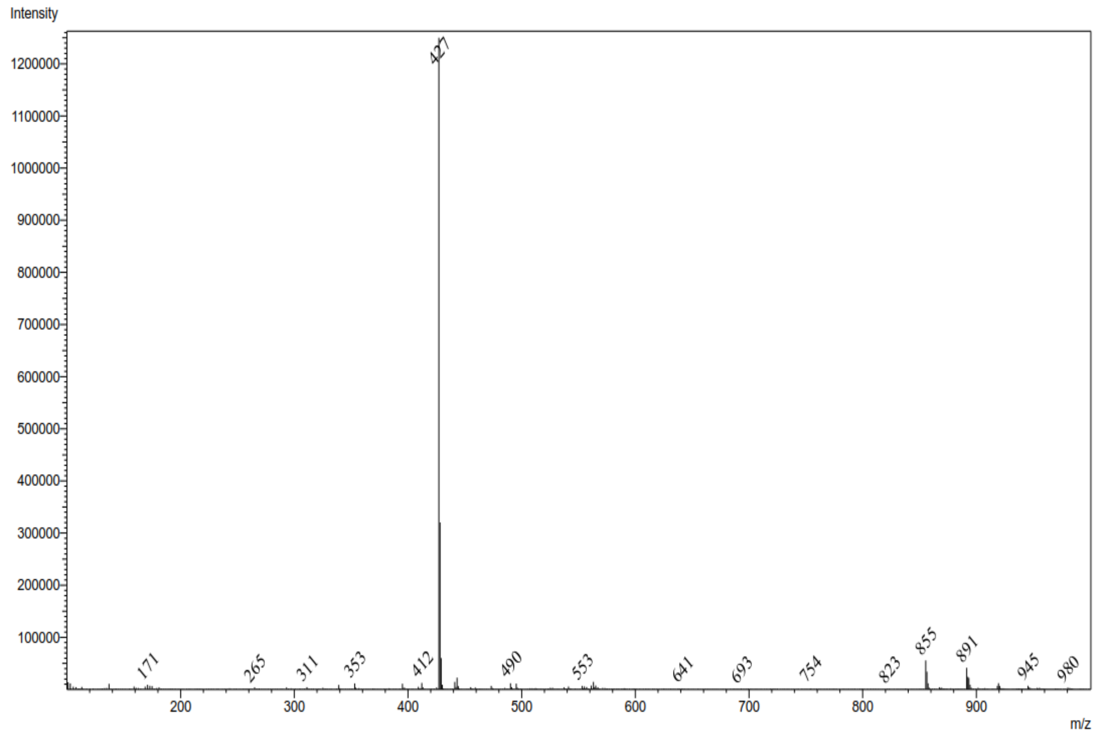

Figure S70. ESI-MS spectrum of compound **17**.

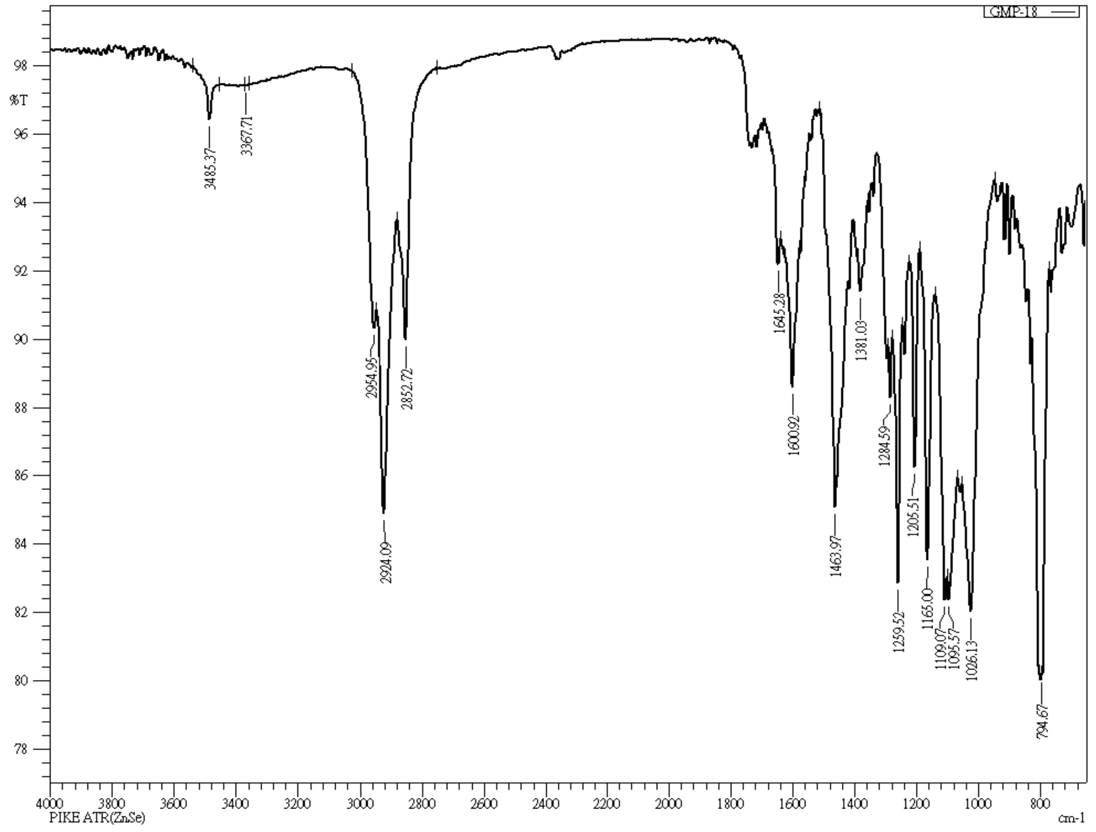

Figure S71. IR spectrum of compound **17**.

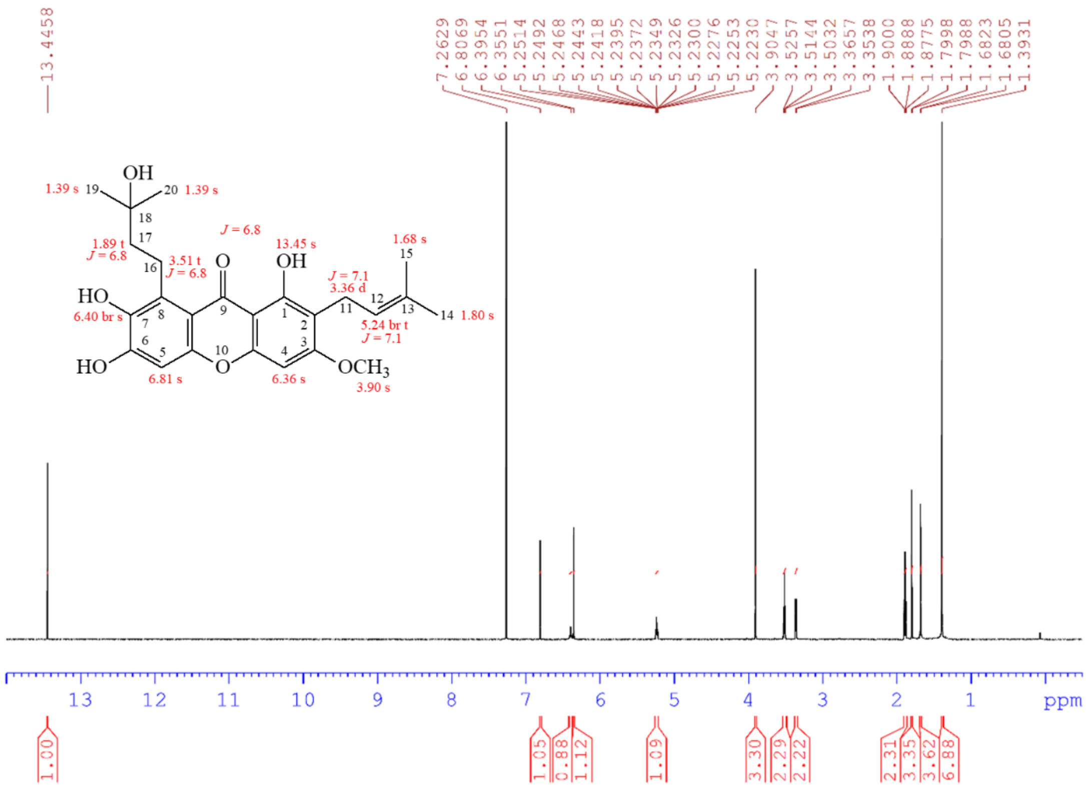

Figure S72. ^{1}H-NMR spectrum of compound **17**.

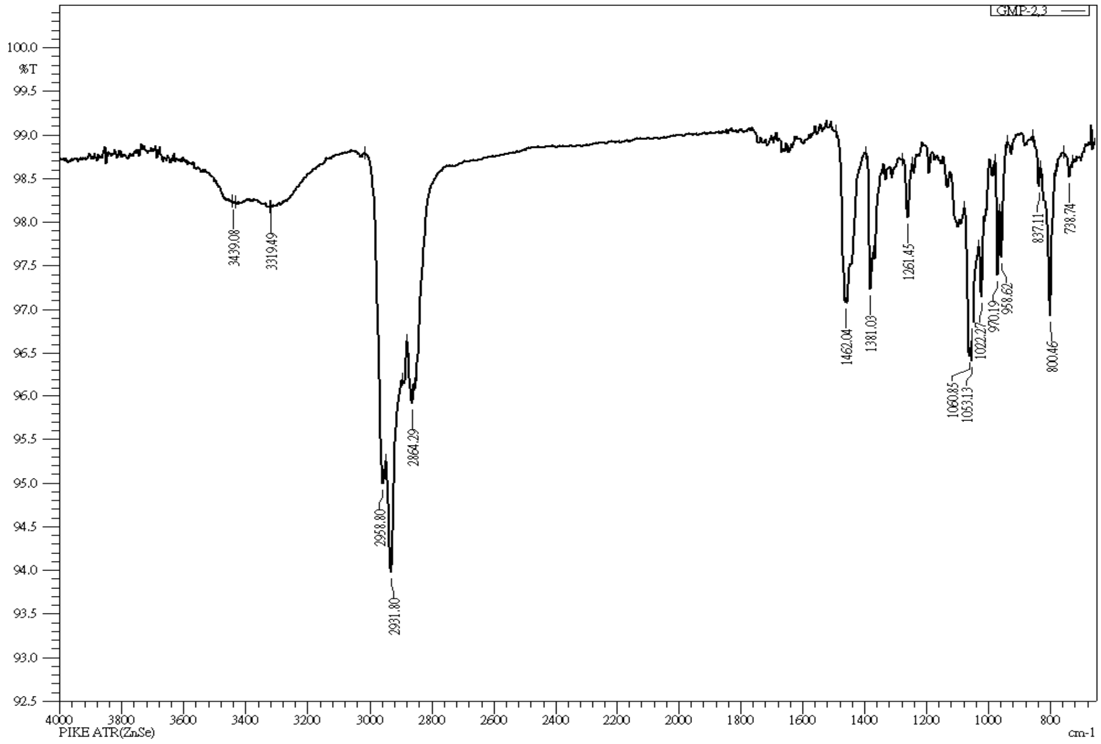

Figure S73. IR spectrum of mixture of compounds **18** and **19**.

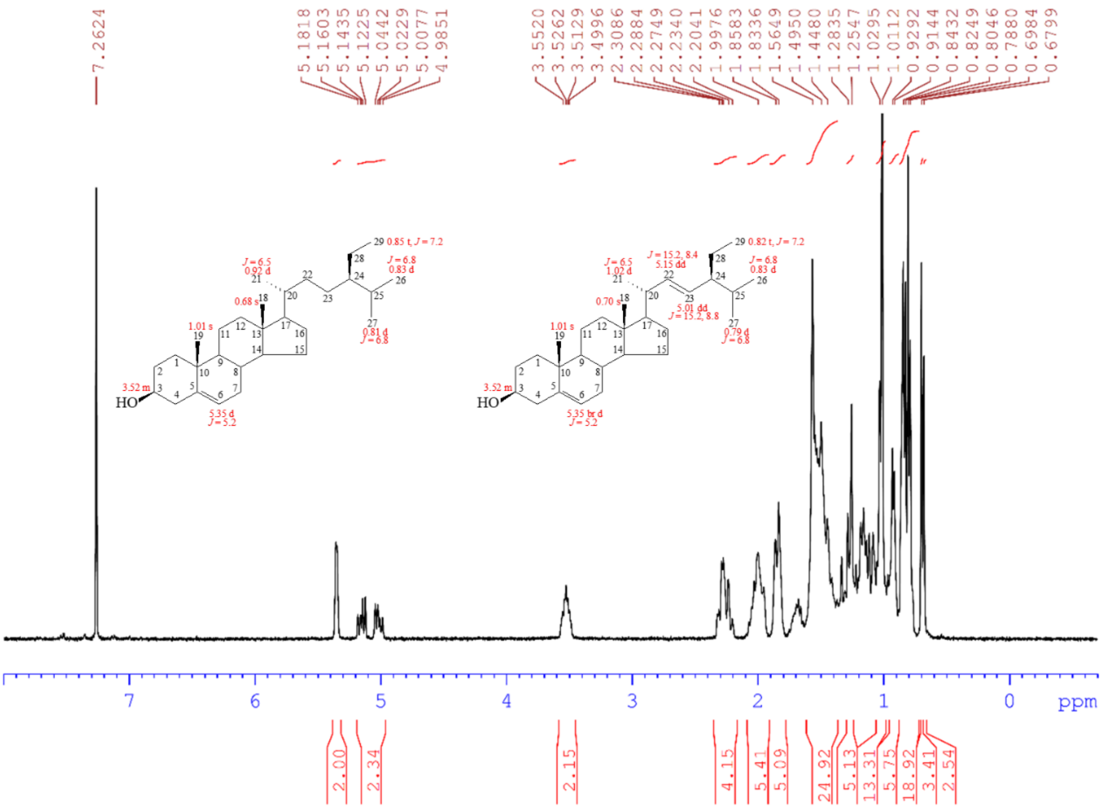

Figure S74. ^{1}H-NMR spectrum of mixture of compounds **18** and **19**.

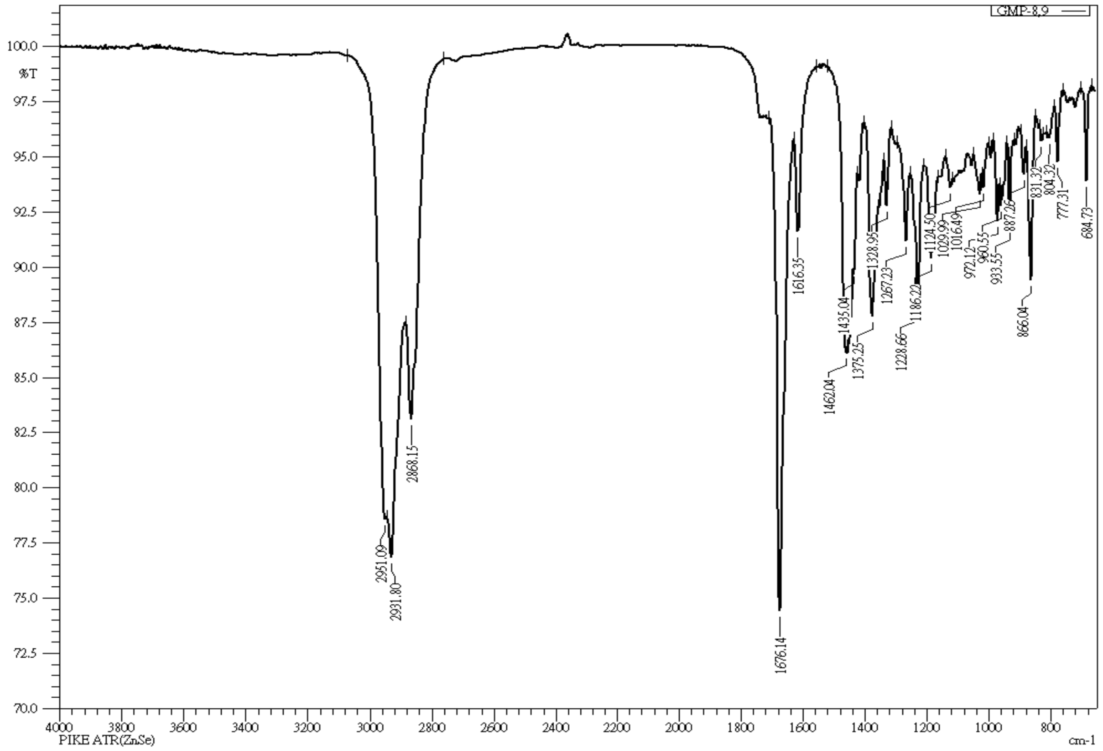

Figure S75. IR spectrum of mixture of compounds **20** and **21**.

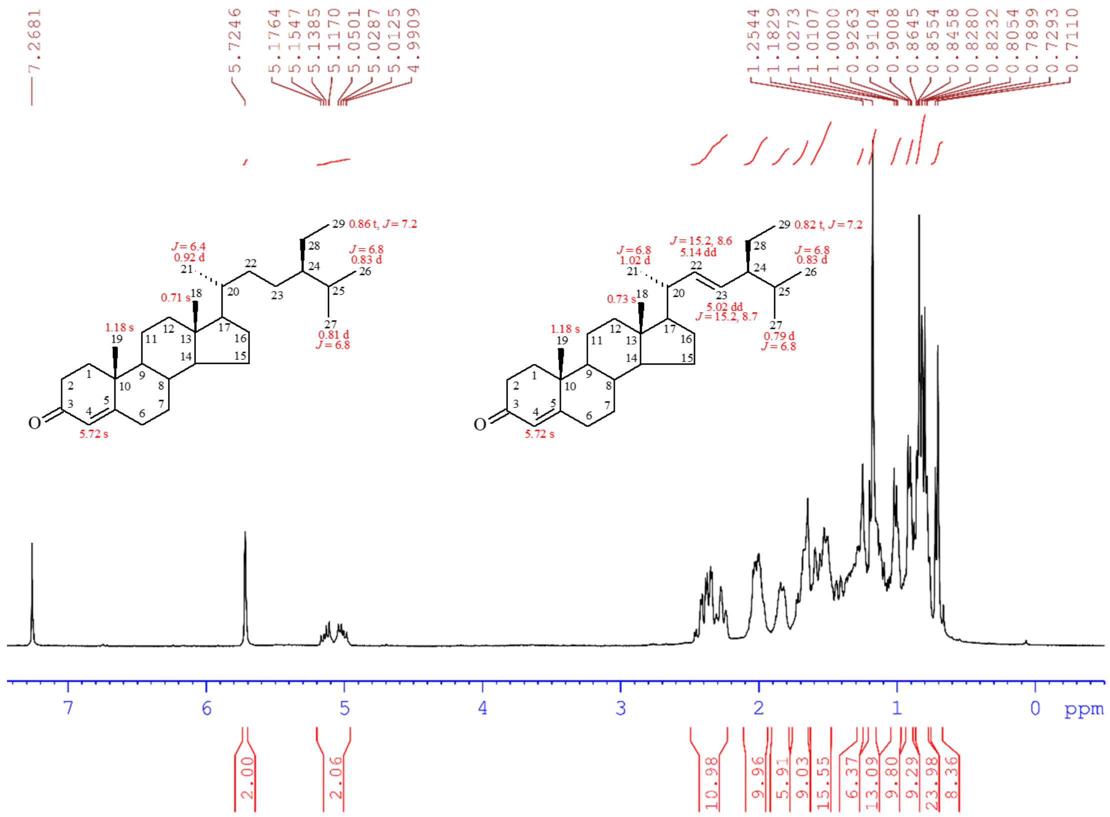

Figure S76. ^{1}H-NMR spectrum of mixture of compounds **20** and **21**.
